# Anion‐Controlled Structural Interconversion of Palladium Cages Enables Separations by Selective Guest Capture and Release

**DOI:** 10.1002/anie.202525696

**Published:** 2026-03-05

**Authors:** Zhe Li, Tanya K. Ronson, Charlie T. McTernan

**Affiliations:** ^1^ Artificial Molecular Machinery Laboratory The Francis Crick Institute London UK; ^2^ Department of Chemistry Kings College London London UK; ^3^ Department of Chemistry University of Cambridge Cambridge UK

**Keywords:** metal–organic cages, purification, selective guest capture, structural interconversion, supramolecular chemistry

## Abstract

Self‐assembled host–guest systems provide a powerful platform for molecular recognition and binding. Achieving the controlled and selective release of bound guests remains challenging, typically relying on destructive stimuli. Herein, we report that asymmetric ligand **L** assembles to form an interconverting pair of palladium(II)‐based metal–organic cages, Pd_4_L_8_(BF_4_)_8_ and Pd_6_L_12_(NTf_2_)_12_, capable of undergoing clean, reversible, and quantitative structural interconversion triggered by specific counter‐anions. The two cages exhibit distinct guest recognition profiles, with each cage binding a different subset of guests. We use anion‐mediated structural transformations to achieve orthogonal, multi‐cycle, selective binding and release of different guest molecules under mild conditions. To showcase the power of this supramolecular catch‐and‐release purification, we designed and validated a closed‐loop purification process, successfully isolating Darunavir from a complex mixture of pharmaceutical molecules with exceptional selectivity and efficiency. This work highlights a broadly applicable strategy for advanced molecular separations and selective pharmaceutical purification.

## Graphical abstract

We report a pair of interconvertible palladium(II) cages assembled from an asymmetric ligand, whose switching is triggered by specific counter‐anions, tetrafluoroborate (BF_4_^−^) and bis(trifluoromethanesulfonyl)imide (NTf_2_^−^). Their complementary guest preferences enable orthogonal, multi‐cycle catch‐and‐release, exemplified by the selective purification of the antiviral drug Darunavir from complex pharmaceutical mixtures.

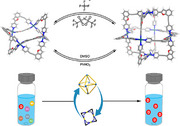

## 1 Introduction

Host–guest chemistry plays a fundamental role in a wide range of fields, including drug delivery [1, 2, 3, 4], molecular sensing [5, 6, 7, 8], and chromatographic purification [9, 10, 11]. Metal–organic cages assembled from metal ions and organic ligands have gained significant attention due to their precisely tunable cavity size, structural diversity, high stability, facile modular synthesis, and adaptability to different stimuli [7, 9, 12, 13, 14, 15, 16]. These properties endow metal–organic cages with the ability to selectively encapsulate a broad spectrum of guest molecules, from ions and small molecule drugs, to peptides and biomolecules [17, 18, 19], making them versatile platforms for targeted recognition, separation, and catalytic transformations [20, 21, 22, 23, 24, 25].

Despite recent advances in selective host–guest chemistry using lower symmetry architectures [26], achieving the controlled, efficient, and selective release of encapsulated guests, particularly in a manner compatible with recyclable use, remains a key challenge [27, 28]. Common strategies for guest release include thermal decomposition, pH‐induced rearrangement [29, 30, 31, 32], and competitive guest exchange [33, 34, 35]. While these approaches can be effective in specific cases, they often lack quantitative control over guest release, and may suffer from limited release efficiency. Moreover, external triggers such as heat or acid/base conditions can be incompatible with sensitive guests, or operationally complex in continuous processes, thereby limiting practical applications involving repeated cycles [2, 36, 37]. To overcome these challenges, researchers have developed a range of stimuli‐responsive cages capable of externally triggered structural modulation. Light‐induced systems offer spatial and temporal control, but typically require high‐energy UV irradiation that risks degrading guests and subcomponents [38, 39, 40, 41]. Redox‐responsive architectures, such as the tetrathiafulvalene‐based cages developed by Sallé and Goeb [42, 43, 44, 45, 46], have demonstrated elegant control of guest binding. However, they often face narrow redox windows and limited compatibility with redox‐active guests [47, 48, 49]. Similarly, while pH‐driven transformations have been successfully employed by Crowley and others to modulate guest release [50, 51, 52], they may sometimes lack precision and reversibility [53, 54]. As such, the development of orthogonal, robust, and fully reversible guest binding and release strategies remains a significant challenge in supramolecular chemistry [55, 56]. In this context, recent studies have demonstrated that solvent polarity and anion templation can direct structural interconversion between discrete metal–organic cages. This opens a promising alternative pathway for implementing stimuli‐gated modulation of cavity environments [35, 57, 58, 59, 60, 61, 62]. Solvent‐ or anion‐triggered transformations could enable a new class of responsive host systems with potential for controlled multi‐cycle guest binding and release.

**FIGURE 1:**
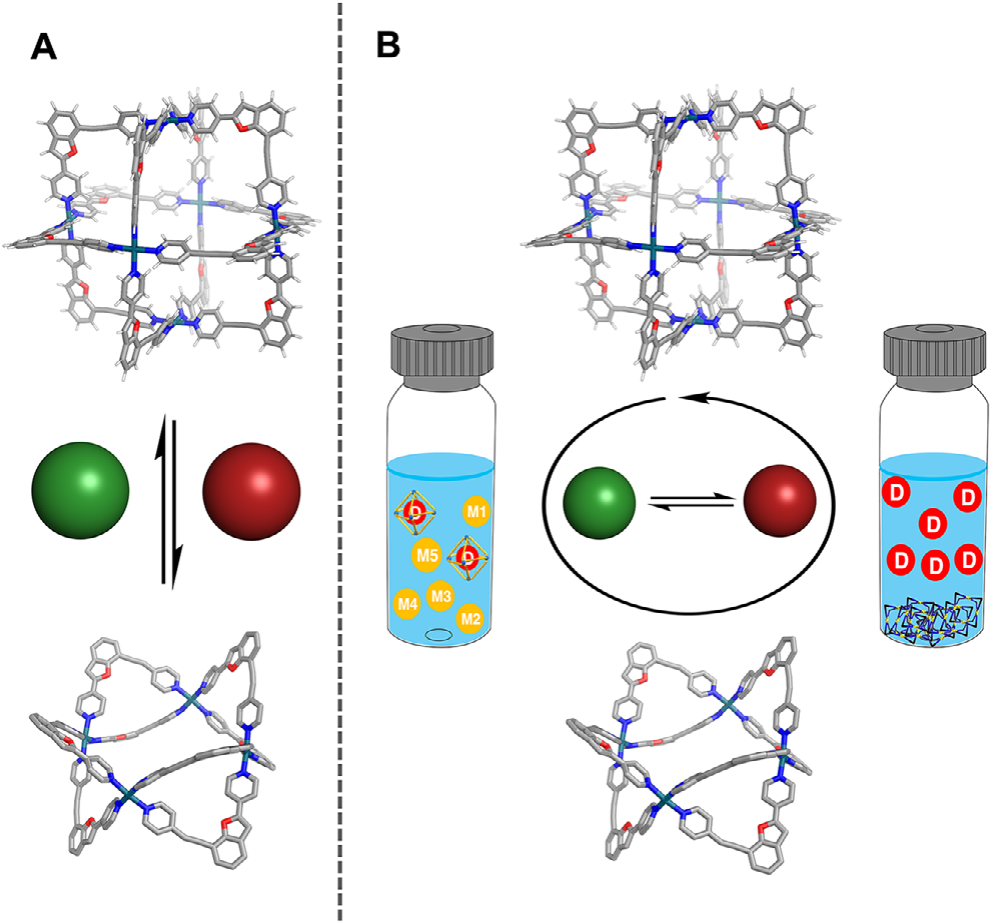
Graphical summary of our work. (A) Reversible interconversion of cages by addition of anions, switching between two structures whilst using the same ligand. (B) Graphical summary of the purification of Darunavir from a complex mixture of pharmaceuticals, triggered by cage interconversion. The green (NTf_2_^−^) and red (BF_4_^−^) spheres represent the anions used to trigger cage interconversion.

Herein, we report a dual‐responsive pair of palladium(II)‐based cages, Pd_4_L_8_(BF_4_)_8_, and Pd_6_L_12_(NTf_2_)_12_, that undergo reversible interconversion in response to both specific anions, and solvent polarity (Figure 1A). These two species differ significantly in geometry, cavity size, and binding selectivity, enabling orthogonal control over the uptake and release of guest molecules *via* structural transformation. Using this mechanism, we achieve multi‐cycle, selective binding and anion‐triggered release of targeted guest species (Figure 1B). Furthermore, we design a purification cycle using this transformation to enable the selective extraction and recovery of a clinically relevant drug molecule, Darunavir, from complex mixtures of pharmaceuticals, demonstrating how this reversible host system can be used for drug recovery and molecular separation.

## 2 Results and Discussion

**FIGURE 2:**
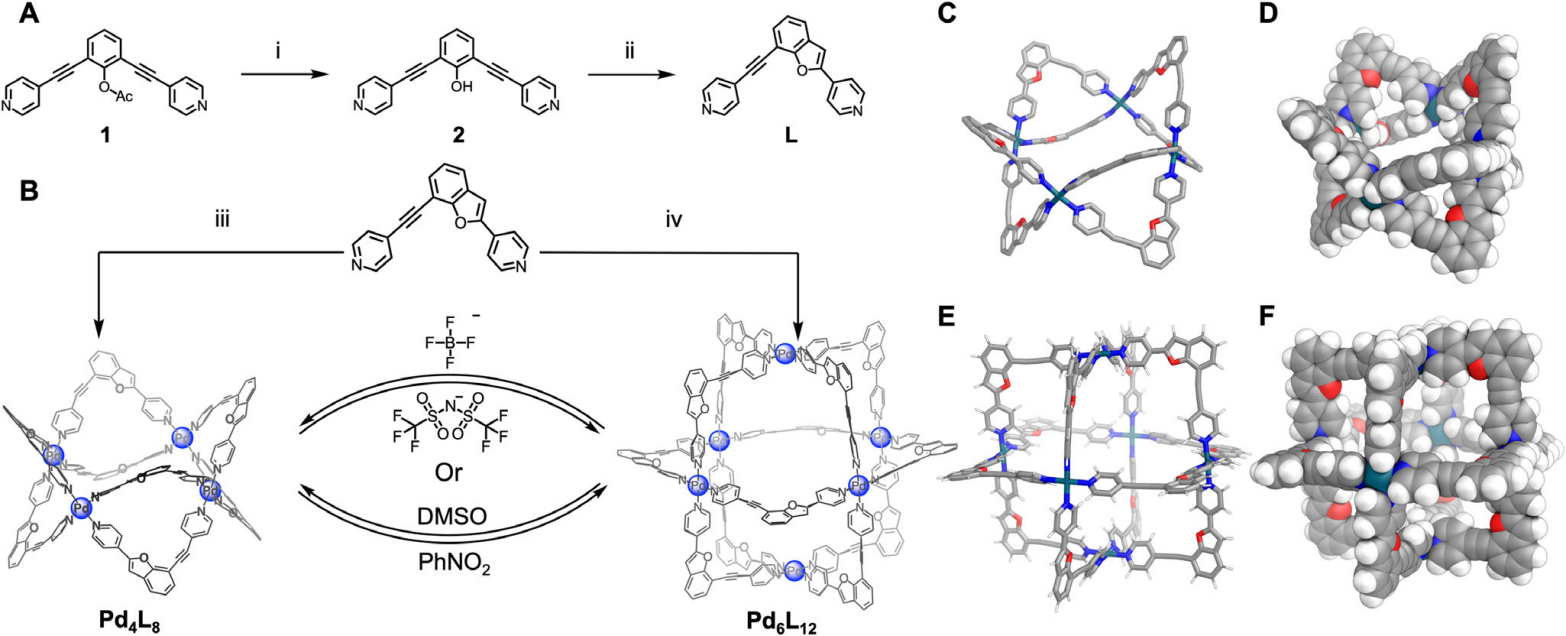
(A) Synthetic route to the benzofuran‐based ligand. Conditions i: NaOH, MeOH; ii: TFA, MeOH. (B) Schematic illustration of the assembly and reversible structural interconversion between the flattened square Cage Pd_4_L_8_(BF_4_)_8_ and the octahedral Cage Pd_6_L_12_(NTf_2_)_12_ triggered by specific anions or solvents. Condition iii: Pd(BF_4_)_2_, MeCN, 50°C; or Pd(CH_3_CN)_4_(OTf)_2_, DMSO, 50°C; iv: PdCl_2_, AgNTf_2_, MeCN, 50°C; or Pd(CH_3_CN)_4_(OTf)_2_, PhNO_2_, 50°C. Cages can be interconverted either by addition of the appropriate anion (top) *or* heating to 50°C in the appropriate solvent. (C and D) X‐ray crystal structure of Cage Pd_4_L_8_(BF_4_)_8_ shown in stick (C) and space‐filling (D) representations. (E and F) DFT‐optimised computational model of structure of Cage Pd_6_L_12_(NTf_2_)_12_ in stick (E) and space‐filling (F) representations.

Cages Pd_4_L_8_(BF_4_)_8_ and Pd_6_L_12_(NTf_2_)_12_ were constructed from a benzofuran‐based ligand (**L**) synthesised *via* an acid‐induced intramolecular cyclisation of a linear precursor (Figure 2A), under different conditions. We initially observed product **L** as a minor byproduct during purification of **2** on silica; likely catalysed by residual acidity. We optimised this process to generate **L** in high yield (Section S2). Ligand **L** provides an unusual coordination system for metal–organic cages, with two pyridine vectors held at c. 90**°**, with two very similar, but not identical, length ‘arms’. Coordination of this ligand with Pd(II) salts under different solvent and counterion conditions led to the formation of two distinct metal–organic cage structures. Specifically, Cage Pd_4_L_8_(BF_4_)_8_ was selectively obtained when BF_4_^−^ was used as the counterion (Pd(BF_4_)_2_, MeCN, 50°C) or when DMSO was used as the solvent (Pd(OTf)_2_, DMSO, 50°C) (Figure 2B). Cage Pd_6_L_12_(NTf_2_)_12_ was preferentially formed either in the presence of NTf_2_^−^ as the counterion (PdCl_2_, AgNTf_2_, MeCN, 50°C) or using PhNO_2_ as the solvent (Pd(OTf)_2_, PhNO_2_, 50°C).

**FIGURE 3:**
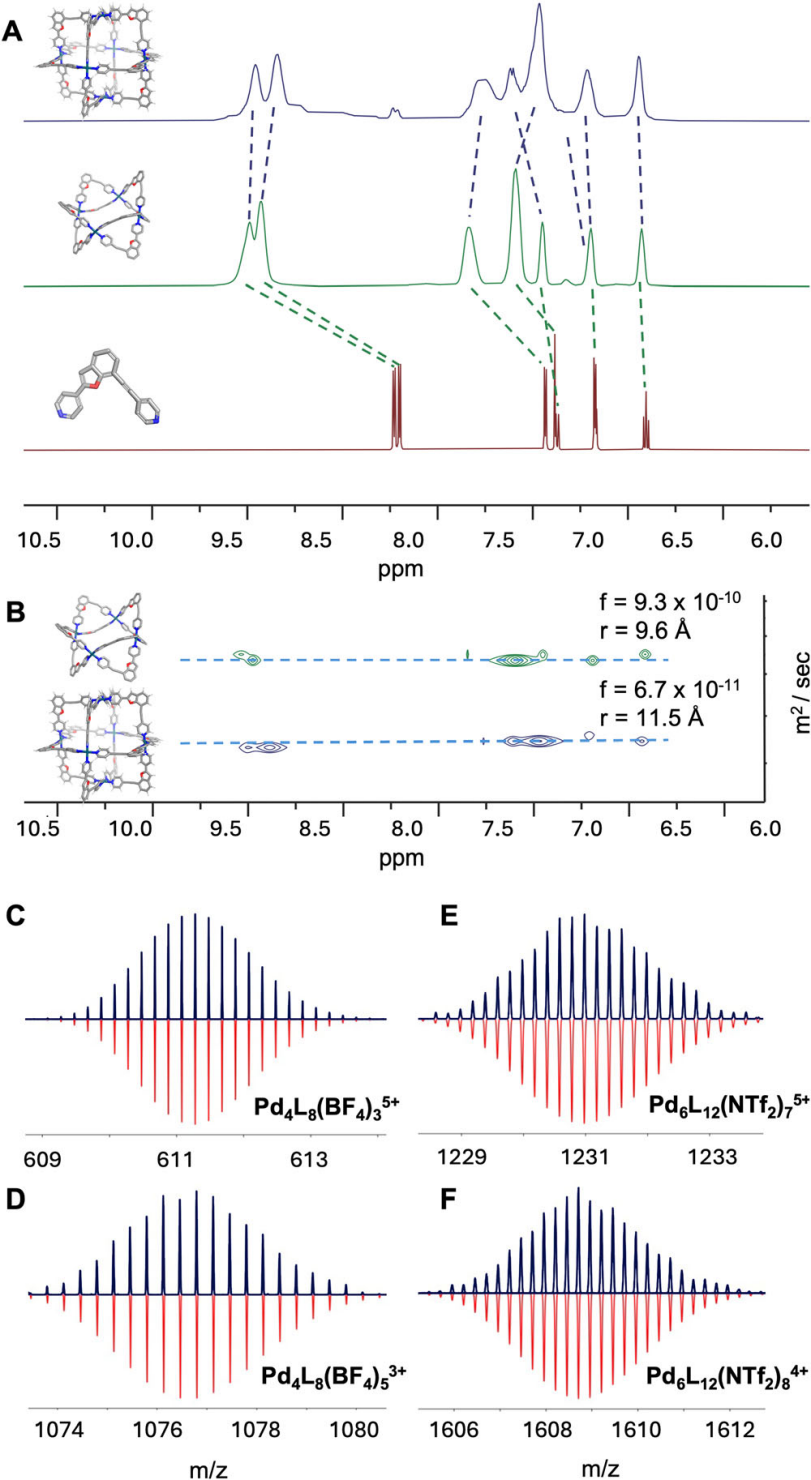
Characterisation of the Pd_4_L_8_(BF_4_)_8_ and Pd_6_L_12_(NTf_2_)_12_. (A) Stacked ^1^H NMR spectra of **L** (bottom), Pd_4_L_8_(BF_4_)_8_ (middle), and Pd_6_L_12_(NTf_2_)_12_ (top) (DMSO‐*d*_6_, 600 MHz, 298K). (B) DOSY NMR spectra showing distinct diffusion coefficients for Pd_4_L_8_(BF_4_)_8_ and Pd_6_L_12_(NTf_2_)_12_ (DMSO‐*d*_6_, 600 MHz, 298K)_,_ correlating to hydrodynamic radii of 9.6 Å for Pd_4_L_8_(BF_4_)_8_, and 11.5 Å for Pd_6_L_12_(NTf_2_)_12_. (C–F) High‐resolution ESI‐MS spectra of Cages Pd_4_L_8_(BF_4_)_8_ and Pd_6_L_12_(NTf_2_)_12_, with excellent agreement between experimental (top, blue) and calculated (bottom, red) isotopic patterns.

The formation, identity, and structural integrity of Cages Pd_4_L_8_(BF_4_)_8_ and Pd_6_L_12_(NTf_2_)_12_ were confirmed by NMR and mass spectrometry. Coordination of the ligand to Pd(II) centres induced significant downfield shifts relative to the free ligand in ^1^H NMR spectra of both Cages Pd_4_L_8_(BF_4_)_8_ + Pd_6_L_12_(NTf_2_)_12_ (Figures 3A, S12, and S32). The peak broadening after assembly may be related to both metal coordination and the coexistence of multiple geometric isomers. This reflects a desymmetrisation and likely dynamic exchange between a range of potential cage isomers within the assemblies, as the two ligand ‘arms’ are insufficiently differentiated to drive assembly of a single cage isomer. Diffusion‐ordered spectroscopy (DOSY) further distinguished the two assemblies. Cage Pd_4_L_8_(BF_4_)_8_ exhibited a diffusion coefficient of 9.3 × 10^−10^ m^2^/s (DMSO*‐d_6_*, 298 K), correlating to a hydrodynamic radius of 9.6 Å (Figure S17), while Cage Pd_6_L_12_(NTf_2_)_12_ diffused at 6.7 × 10^−11^ m^2^/s (DMSO*‐d_6_*, 298 K), consistent with a larger hydrodynamic radius of 11.5 Å (Figures 3B and S36). The estimated molecular weights derived from these values were in close agreement with the theoretical molecular masses of the respective cages, supporting the formation of two discrete architectures of different sizes. High‐resolution ESI mass spectrometry (HRMS) provided confirmation of cage composition. The observed *m/z* values and isotopic distributions for [Pd_4_L_8_] (Figures 2C+D and S22–S26), and [Pd_6_L_12_] (Figures 3E+F and S36–S41) closely matched simulated patterns, validating proposed Pd‐to‐Ligand **L** stoichiometries. Notably, under all conditions investigated, both cages can exist as mixtures of geometric isomers, without strong preferences for a specific configuration under equilibrium conditions. This is supported by the ^1^H NMR behaviour of Pd_4_L_8_ in CD_3_CN, where the spectra display high complexity with multiple unresolvable peaks (see Figure S22), indicative of a complex isomeric mixture. The statistical head‐to‐tail disorder of the ligands in the x‐ray crystal structure also confirms the lack of a strong preference for a single geometric configuration. This is likely due to the structural similarity of the two different ligand arms.

Crystals suitable for single‐crystal x‐ray diffraction analysis were grown from vapour diffusion of diisopropyl ether into a solution of cage Pd_4_L_8_(BF_4_)_8_ in nitrobenzene. Analysis revealed that Cage Pd_4_L_8_(BF_4_)_8_ adopts a flattened square geometry, with four edges bridged by eight ligands aligned in an alternating up–down configuration (Figures 2C,D and Section S7) in a cyclic array. Each ligand was disordered head‐to‐tail with equal occupancy; a single isomer of the cage is illustrated in Figure 2C+D for clarity. We extensively screened conditions to crystallise Cage Pd_6_L_12_(NTf_2_)_12_ (>200 attempts) but no suitable crystals were formed, with issues with poor diffraction and rapid crystal decomposition on removal from solvent proving insurmountable. Although crystallographic characterisation of Cage Pd_6_L_12_(NTf_2_)_12_ was not successful, we employed DFT calculations (Section S8) to investigate the two most likely structures for a Pd_6_L_12_‐type cage: a hexagonal ring, corresponding to an enlarged version of the structure of Cage Pd_4_L_8_(BF_4_)_8_, and an octahedral configuration, more commonly observed for Pd_6_L_12_ cages in the literature [63, 64]. In each case, a handful of arbitrarily chosen isomers were modelled. Geometry optimisations use the advanced r2SCAN‐3c method. Representative sampling of the conformational space revealed that the octahedral topology is consistently lower in energy than the hexagonal macrocycle (by > 113 kJ/mol), regardless of the specific ligand head‐to‐tail arrangement (Figures 2E,F). This is consistent with the significant differences seen in the ^1^H NMR spectra, particularly around 7.5 ppm (Figure 3A), suggesting structural rearrangement between the two species. This, along with the preponderance of octahedral Pd_6_L_12_ structures reported in the literature leads us to tentatively assign Cage Pd_6_L_12_(NTf_2_)_12_ as a distorted octahedral cage [65, 66, 67, 68]. The different isomers of the octahedral topology were much more similar in energy (<15 kJ/mol), consistent with our assignment of a lack of isomer control in the assembly (Section S8).

With the structures of the discrete Pd_4_L_8_ and Pd_6_L_12_ assemblies assigned, we investigated mechanisms to enable their interconversion. The transformation between the two geometries can be triggered by modulating either the counter‐anion or the solvent environment (Figure 2B), with distinct efficiencies. The anion‐driven transformation proceeds with quantitative conversion. Addition of excess tetrafluoroborate salts (BF_4_^−^) to a solution of Cage Pd_6_L_12_(NTf_2_)_12_ triggers a rapid and complete reorganisation to form Cage Pd_4_L_8_(BF_4_)_8_ (Figures S98+S99). Conversely, treating the Pd_4_L_8_ species with NTf_2_^−^ anions regenerates the Pd_6_L_12_(NTf_2_)_12_ architecture (Figures S95+S96). In both directions, the conversion is complete, with no residual signals of the starting cage observed by HRMS. The interconversion can also be driven by heating in certain solvents, while exhibiting significant kinetic stability at room temperature. Cage Pd_4_L_8_ remains stable in nitrobenzene (which favours Pd_6_L_12_) at room temperature, enabling the growth of single crystals of Pd_4_L_8_ without conversion. Solvent‐mediated transformation requires thermal activation (50°C) and shifts the distribution to favour the target cage as the major species, but minor amounts of the starting architecture often persist (Figures S97, S100). Dissolution of Pd_6_L_12_ in DMSO, followed by heating, causes predominant formation of Pd_4_L_8_ architectures (Figure S100). As the efficiency of switching was higher in the anion‐mediated pathway, achieving quantitative and residue‐free switching, we chose to use this method in subsequent studies. The clean switching highlights the structural responsiveness of the system (Figure 2B, Section S4).

**FIGURE 4:**
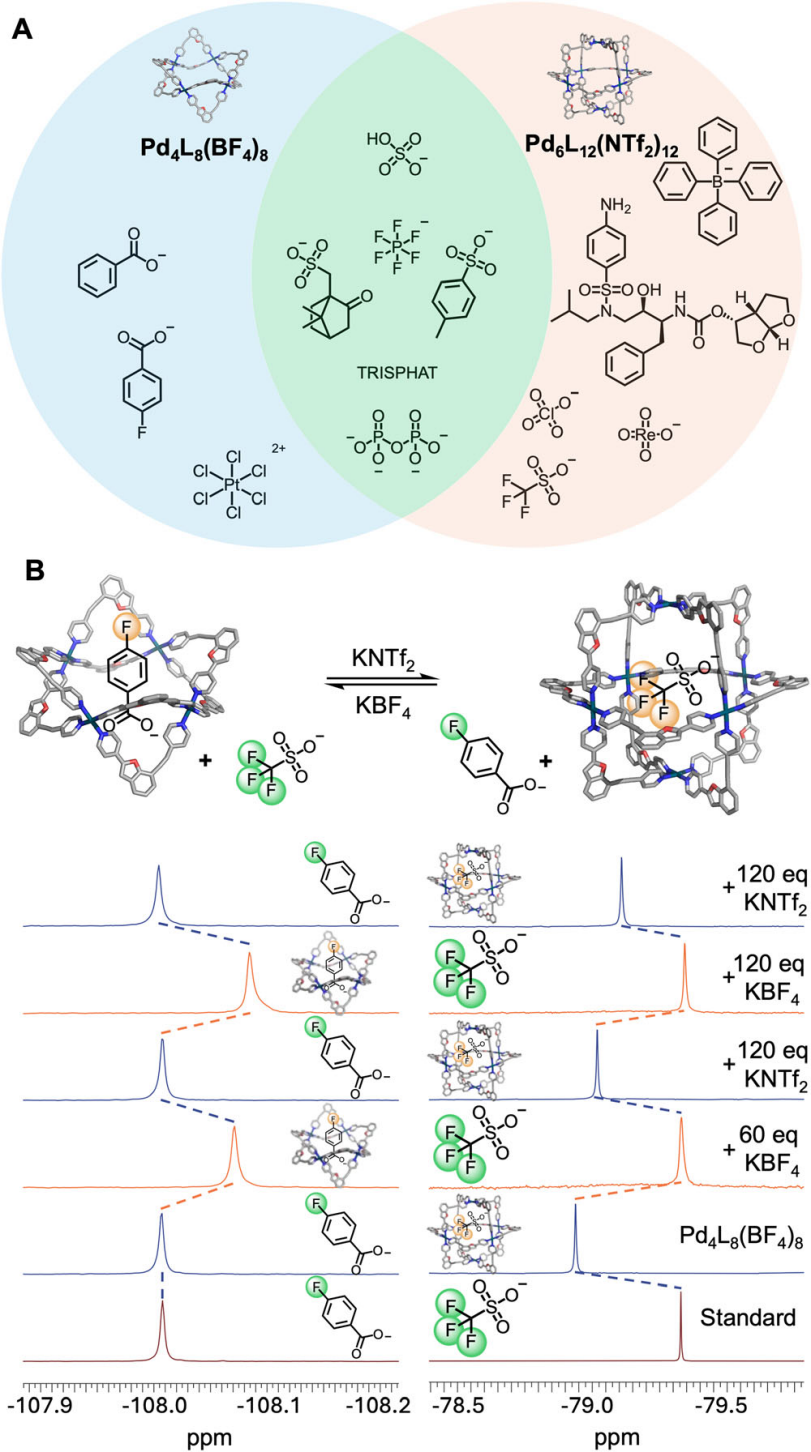
(A) Guest binding behaviour of Cages Pd_4_L_8_(BF_4_)_8_ and Pd_6_L_12_(NTf_2_)_12_ with selected targets. Guests are categorised into three types based on their binding selectivity: preferential binding to Cage Pd_4_L_8_(BF_4_)_8_ (blue), to Cage Pd_6_L_12_(NTf_2_)_12_ (orange), or to both (overlapped, green). (B) Reversible and selective guest uptake and release using structural interconversion of Cages (MeCN‐*d*_3_, 376 MHz, 298K). Cage Pd_6_L_12_(NTf_2_)_12_ was mixed with equimolar 4F‐benzoate (4F‐BA^−^) and triflate (OTf^−^), then subjected to repeated cycles of cage interconversion triggered by addition of BF_4_^−^ and NTf_2_^−^. Stacked ^19^F NMR spectra showing the guest binding behaviour through two cycles.

​We next investigated the guest binding behaviour of the interconvertible cage pair. Host–guest experiments were carried out by adding 1.0 equiv. of each guest to solutions of Cage Pd_4_L_8_(BF_4_)_8_ and Cage Pd_6_L_12_(NTf_2_)_12_, with all guests which bound showing fast‐exchange binding. A screen of various anionic and neutral guests revealed that Cages Pd_4_L_8_(BF_4_)_8_ and Pd_6_L_12_(NTf_2_)_12_ exhibit distinct, yet partially overlapping, binding preferences (Figure 4A). To quantify these interactions, ^1^H NMR titrations were performed with a library of representative guests. The binding data fitted well to 1:1 or 1:2 binding models, allowing the determination of association constants (K_a_) (Section S3).

Certain guests were exclusively encapsulated by one cage, with Cage Pd_4_L_8_(BF_4_)_8_ preferring smaller compounds such as benzoic acids and PtCl_6_^2−^, and Cage Pd_6_L_12_(NTf_2_)_12_ showing wider affinity for larger structures such as Darunavir and BPh_4_^−^, consistent with Cage Pd_6_L_12_(NTf_2_)_12_’s greater size. Other guests were bound by both Cages Pd_4_L_8_(BF_4_)_8_ and Pd_6_L_12_(NTf_2_)_12_, such as hydrogen sulfate, pyrophosphate, and PF_6_^−^. This divergence in guest selectivity enables orthogonal uptake and release pathways upon cage interconversion, forming the basis for switchable and selective host–guest modulation.

To effect this, we first designed a guest‐exchange experiment using 4‐fluorobenzoate (4F‐BA^−^, which binds exclusively to Cage Pd_4_L_8_(BF_4_)_8_) and triflate (OTf ^−^, which binds exclusively to Cage Pd_6_L_12_(NTf_2_)_12_) as representative orthogonal guests to demonstrate efficacy. These guests were simultaneously added to a solution of Cage Pd_6_L_12_(NTf_2_)_12_, where NMR spectroscopy revealed characteristic binding features: the ^19^F NMR resonance of OTf ^−^ underwent a marked downfield shift, indicative of encapsulation within Pd_6_L_12_(NTf_2_)_12_, while the ^19^F NMR signals of 4F‐BA^−^ remained largely unchanged, suggesting minimal interactions with this cage. Subsequently, addition of 60 equivalents (relative to cage; 5 equivalents relative to NTf_2_^−^) of BF_4_^−^, followed by heating at 50°C, triggered a complete transformation of Cage Pd_6_L_12_(NTf_2_)_12_ into Cage Pd_4_L_8_(BF_4_)_8_ (Figure S101+S102). This structural switch led to the release of OTf ^−^, and concurrent encapsulation of 4F‐BA^−^, evidenced by the chemical shifts of their ^19^F NMR peaks. Reversing the transformation by adding 120 equivalents of NTf_2_^−^ relative to cage regenerated Cage Pd_6_L_12_(NTf_2_)_12_, with corresponding exchange of guests, such that 4F‐BA^−^ was released, and OTf ^−^ rebound.

This guest exchange cycle was repeated four times, with consistent and reversible spectral changes observed in ^19^F NMR (Figure 4B, first 2 cycles; full cycle stack Figure S101). Each cycle was conducted continuously without purification, by cumulatively adding respective anions. At each step, a sufficient excess was used to favour conversion to the target cage isomer. Notably, no degradation or diminished efficiency was observed over these cycles, confirming recyclability and efficacy. These results illustrate how orthogonal guest affinities, coupled with anion‐gated isomerisation, can be synergistically harnessed for precise control over guest binding and release in supramolecular host–guest systems.

**FIGURE 5:**
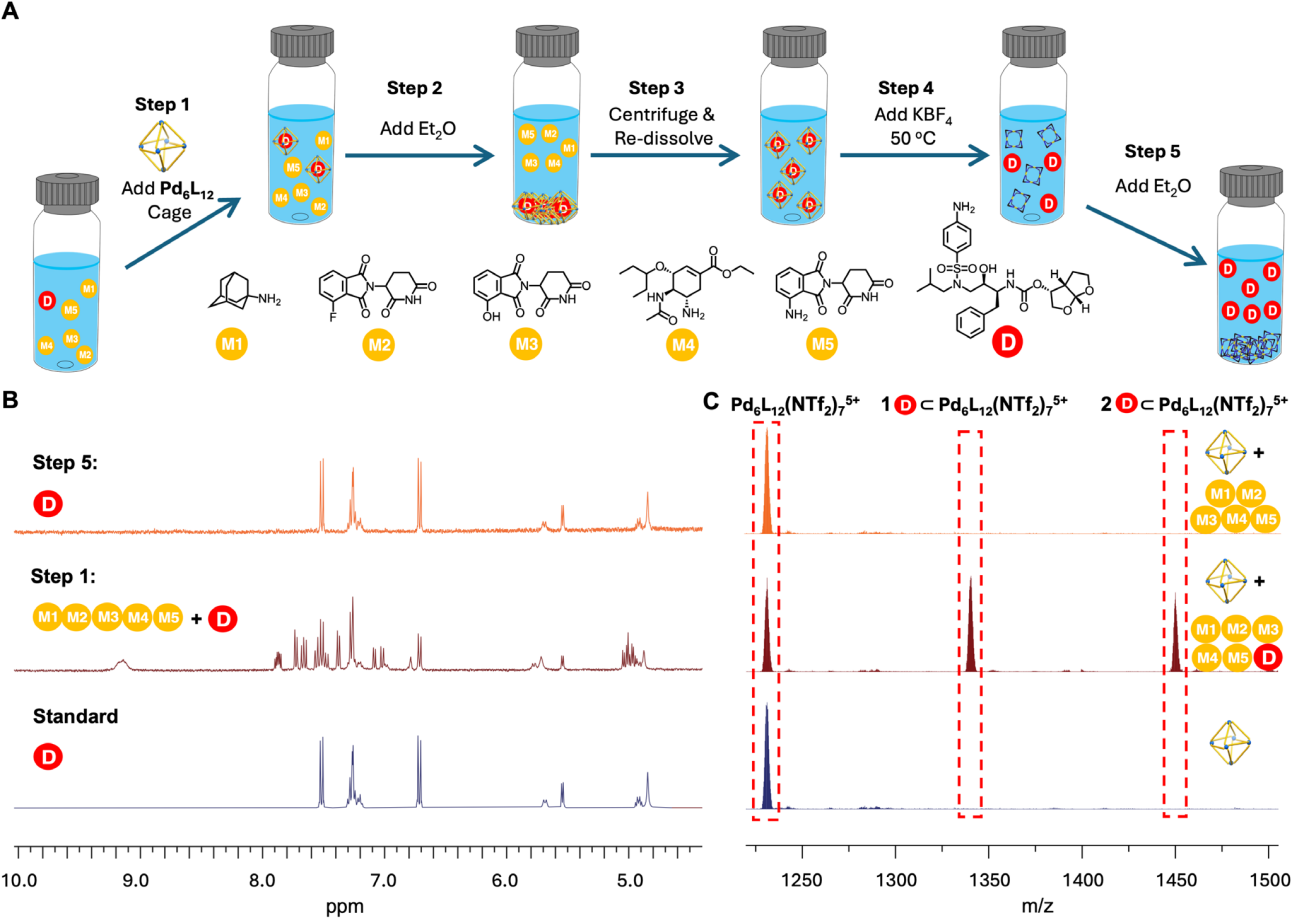
Guest‐selective cage transformation for purification of Darunavir from a mixed pharmaceutical matrix. (A) Schematic illustration of the Cage Pd_6_L_12_(NTf_2_)_12_‐to‐Cage Pd_4_L_8_(BF_4_)_8_ transformation strategy used for selective extraction and purification of Darunavir (D) from a mixture of six structurally differentiated pharmaceutical molecules (**D** and **M1–M5**). **Step 1**: 2 equiv. of Cage Pd_6_L_12_(NTf_2_)_12_ is added to a CH_3_CN solution containing a 1:1:1:1:1:1 mixture of **D** and **M1–M5**; **Step 2**: Excess Et_2_O is added, precipitating the **D**‐loaded Cage Pd_6_L_12_(NTf_2_)_12_; **Step 3**: Precipitate is collected by centrifugation, washed and re‐dissolved in CH_3_CN; **Step 4**: BF_4_^−^ (10 eq) is added with heating to induce Cage Pd_6_L_12_(NTf_2_)_12_‐to‐Pd_4_L_8_(BF_4_)_8_ transformation and release of **D** into solution; **Step 5**: Addition of Et_2_O leads to precipitation of Cage Pd_4_L_8_(BF_4_)_8_, leaving **D** in solution. **Step 6**: Centrifugation to recover Cage Pd_4_L_8_(BF_4_)_8_ and purified **D**. Addition KNTf_2_/MeCN to convert Cage Pd_4_L_8_(BF_4_)_8_ to Cage Pd_6_L_12_(NTf_2_)_12_ enables use for next cycle. (B) ^1^H NMR (MeCN‐*d*_3_, 400 MHz, 298K) spectra of i) isolated Darunavir as a standard; ii) **Step 1**: mixture of **D** and **M1–5**; iii) **Step 5**: pure **D** isolated after purification cycle. (C) HRMS spectra of i) Cage Pd_6_L_12_(NTf_2_)_12_; ii) Cage Pd_6_L_12_(NTf_2_)_12_ with mixture of **D** and **M1–5**, and iii) Cage Pd_6_L_12_(NTf_2_)_12_ with just **D**.

To further showcase the functional utility of our anion‐mediated interconvertible cage system, we devised a five‐step extraction‐release‐purification cycle aimed at isolating the antiretroviral drug Darunavir from a complex pharmaceutical mixture of six compounds: **M1**: Amantadine; **M2**: 4‐Fluoro‐Thalidomide; **M3**: 4‐OH‐Thalidomide; **M4**: Oseltamivir; **M5**: Thalidomide; and **D**: Darunavir (Figure 5). Darunavir is selectively bound by Cage Pd_6_L_12_(NTf_2_)_12_, but not by Cage Pd_4_L_8_(BF_4_)_8_. No other compound in our mixture was bound by either cage (Figure S103). As such, we hypothesised that Cage Pd_6_L_12_(NTf_2_)_12_ could selectively isolate Darunavir in its internal cavity. Precipitation would allow collection of the target, before ion exchange triggers release of Darunavir from the non‐binding Cage Pd_4_L_8_(BF_4_)_8_ (Figure 5).

Precipitation of Cage Pd_4_L_8_(BF_4_)_8_ would then deliver pure Darunavir in solution, and isolated cage ready for transformation to Cage Pd_6_L_12_(NTf_2_)_12_ ready for another round of purification (Figure 5). As illustrated in Figure 5A, the initial acetonitrile solution contained six pharmaceutically relevant compounds (**M1–M5** and Darunavir, **D**) in an equimolar ratio (Figure S103). Upon addition of Cage Pd_6_L_12_(NTf_2_)_12_ (Step 1), Darunavir selectively bound to the cage, while the other molecules remained unbound, as assayed by ^1^H NMR and HRMS (Figure 5C). Subsequent addition of excess diethyl ether (Step 2) induced precipitation of the Darunavir‐loaded Cage **Pd_6_L_12_(NTf_2_)_12_**, enabling facile separation from the free **M1–M5** which remain in the supernatant. After centrifugation and re‐dissolution of the precipitate (Step 3), the system now contains only **D**⊂Cage Pd_6_L_12_(NTf_2_)_12_ host–guest complexes in solution. To release Darunavir, we then added 10 equiv alents of BF_4_^−^ followed by heating (Step 4), triggering Cage Pd_6_L_12_(NTf_2_)_12_‐to‐Pd_4_L_8_(BF_4_)_8_ structural transformation. This process disrupted the favourable host–guest interactions, releasing Darunavir into solution. A final diethyl ether‐induced precipitation (Step 5) removed Cage Pd_4_L_8_(BF_4_)_8_, affording a Darunavir‐purified supernatant, free of all other pharmaceutical components and the metal–organic host (Figure 5B). Crucially, ICP‐MS analysis revealed that the purified Darunavir contained negligible residual palladium (< 1 ppb), well below pharmaceutical safety limits and comparable to commercial standards. Furthermore, the recovery yield was determined to be >95%, underscoring the efficiency of the process. Notably, the Cage Pd_4_L_8_(BF_4_)_8_ precipitate could be reused to initiate another capture‐release cycle, showing the recyclability of our system.

## 3 Conclusions

This remarkable efficiency in separation is especially surprising, given our use of a cage system consisting of an unresolved mixture of isomers. However, we believe that this isomeric complexity could be an advantage in this system. Whilst supramolecular design often prioritises high‐symmetry, discrete architectures, such rigid hosts can struggle to accommodate low‐symmetry, conformationally flexible guests, including most pharmaceutical targets. By utilising an asymmetric ligand, our system accesses a library of isomeric binding pockets, which are more likely to be able to bind to pharmaceutically relevant scaffolds. Further, the intrinsic strain implicit in structures containing non‐idealised ligands, might promote relatively accessible interconversion pathways, such as our anion‐mediated transformations. It is also important to note that whilst single isomer assemblies are often aesthetically pleasing, they might not be optimal or optimised for real‐world applications. As such, we believe that the use of simple, asymmetric ligands, to generate complex isomeric mixtures might provide an accessible, and underexplored, route to complex function in self‐assembled systems, providing optimal solutions to intractable challenges.

In summary, we report an anion‐mediated, reversible, structural interconversion between two discrete palladium‐based Cages, Pd_4_L_8_(BF_4_)_8_ and Pd_6_L_12_(NTf_2_)_12_, which exhibit orthogonal guest recognition behaviours. The interconversion is precisely regulated by specific anionic stimuli and solvent conditions. The two cage states to selectively encapsulate geometrically and chemically distinct guests. Our switchable system enables repeated and controlled guest exchange, exemplified by the selective capture and release of Darunavir from complex mixtures of pharmaceuticals through a cage‐mediated separation cycle. Our results demonstrate a generalisable strategy for molecular purification using structurally responsive metal–organic cage partners, offering new design principles for supramolecular separation, targeted delivery, and adaptive host–guest systems.

## Author Contributions

Z.L. conceived and designed the project with input from C.T.M. All experiments, characterisation, and DFT calculations were performed by Z.L. The data were analyzed by Z.L. and C.T.M. T.K.R. solved the crystal structure of Pd_4_L_8_(BF_4_)_8_ and analysed arising data. The manuscript was drafted by Z.L. and C.T.M. C.T.M. supervised the project and secured funding. All authors contributed to the revision of the final manuscript.

## Conflicts of Interest

The authors declare no conflicts of interest.

## Associated Content

CCDC 2499075 contains the supplementary crystallographic data for this paper. These data can be obtained free of charge via www.ccdc.cam.ac.uk/data_request/cif, or by emailing data_request@ccdc.cam.ac.uk, or by contacting The Cambridge Crystallographic Data Centre, 12 Union Road, Cambridge CB2 1EZ, U.K.; fax: +44 1223 336033.

## Supporting information




**Supporting File 1**: anie71642‐sup‐0001‐SuppMat.pdf.

**Supporting File 2**: anie71642‐sup‐0002‐DataFile.txt.

## Data Availability

The data that support the findings of this study are available in the supplementary material of this article.
