# Supplementary material for: Anion‐Controlled Structural Interconversion of Palladium Cages Enables Separations by Selective Guest Capture and Release
Source: Angew Chem Int Ed Engl. 2026 Mar 5;65(16):e25696. doi: 10.1002/anie.202525696 (PMC13080429; doi:10.1002/anie.202525696)
Supplement: Supplementary file 1 — Supporting File 1: anie71642‐sup‐0001‐SuppMat.pdf. [file ANIE-65-e25696-s001.pdf]

Anion-Controlled Structural Interconversion of Palladium Cages Enables Separations by Selective Guest Capture and Release

Zhe Li,[a,b] Tanya K. Ronson,[c] and Charlie T. McTernan[a,b,*]

[a]Artificial Molecular Machinery Laboratory, The Francis Crick Institute, 1 Midland Road, London NW1 1AT, UK.

[b]Department of Chemistry, King's College London, Britannia House, 7 Trinity Street, London SE1 1DB, UK.

[c]Department of Chemistry, University of Cambridge, Lensfield Road, Cambridge CB2 1EW, UK.

[*]Corresponding Author.

Table of Contents

1. **General Experimental** **3**
2. **Synthetic Experimental Procedures** **4**
3. **NMR Host-Guest Association Studies** **34**
4. **Cage Interconversion Tests** **62**
5. **Reversible Guest Binding** **66**
6. **Cage Transformation for Darunavir Purification** **69**
7. **X-Ray Crystallography** **74**
8. **DFT calculations** **76**
9. **References** **82**

1. General Experimental

***Synthesis*:** Unless otherwise stated, all reagents, including anhydrous solvents, were purchased from commercial sources and used without further purification. Reactions were carried out under ambient conditions unless otherwise stated. An SP Scientific Bench Top Pro Lyophilizer was used for drying the samples when required. Chromatographic separation was performed using a Biotage Isolera equipped with Biotage Sfar Duo column cartridges of appropriate phase and size. Unless otherwise stated, crude material was purified by dissolution in a minimum volume of solvent and loaded directly onto equilibrated cartridges.

***Analysis*:** NMR spectra were acquired on a Bruker Ascend 400 equipped with a BBO Smart Probe, an Avance III 600 equipped with a $^{1}H/^{13}C/^{15}N$ triple-resonance PFG cryoprobe, a Bruker Avance NEO 600 MHz equipped with a TCI Cryoprobe Prodigy $^{1}H/^{13}C/^{15}N$ a Bruker Avance III 700 MHz equipped with a QCI Cryoprobe $^{1}H/^{13}C/^{15}N/^{31}P$ or an Avance III HD 950 equipped with a $^{1}H/^{13}C/^{15}N$ triple-resonance PFG cryoprobe at a constant temperature of 298 K unless stated otherwise. Chemical shifts are reported in parts per million from low field to high field, and referenced to residual solvent or internal standard. Coupling constants (J) are reported in Hertz (Hz), and standard abbreviations indicating multiplicity were used as follows: m = multiplet, sext = sextet, quint = quintet, q = quartet, t= triplet, d = doublet, s = singlet, app = apparent and br = broad. Signal assignment was carried out using 2D NMR methods (HSQC, HMBC, COSY, NOESY, ROESY) where necessary.

Low resolution mass spectrometry and UPLC analysis was performed on a Waters Acquity H-class UPLC coupled with a Waters SQD2 mass spectrometer. Chromatographic separation was performed on an ACQUITY UPLC BEH C18 column (130 Å, 1.7 μm, 2.1 x 50 mm) with the following conditions: Solvent A: H_2O:MeCN (95:5) + 2 mM ammonium acetate; Solvent B: MeCN; gradient of 0 – 100% B over 2 min. The instrument was operated at a flow rate of 0.8 mL min^{-1} with a column temperature of 45 °C and an injection volume of 5 µL.

ESI-HRMS were recorded on an Orbitrap Exploris 120 mass spectrometer. The ESI source was operated in positive-negative switching mode at spray voltages of 3500 and -2500 V in the positive and negative modes, respectively. Sheath gas was set to 35, auxiliary gas to 7 and sweep gas to 1. The ion transfer tube temperature was set to 280 °C and the vaporizer temperature was set to 250 °C. A mass range of 400 – 3000 Da was scanned at a resolution of 120,000. Unless otherwise stated, all solutions used for ESI-HRMS analysis were diluted with MeCN to ~ 100 μM.

2. Synthetic Experimental Procedures

Synthesis of 2,6-bis(4-pyridylethynyl)phenol (2)

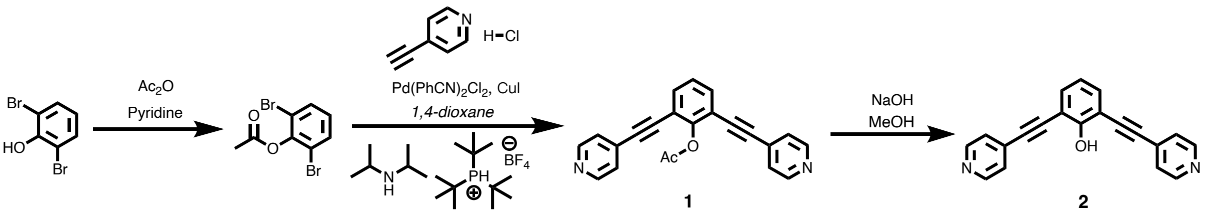

2,6-bis(4-pyridylethynyl)phenol (**2**) was synthesized according to the literature procedure without modification.[1] The product was obtained as a white powder in 72% yield. The characterization data are consistent with those previously reported.

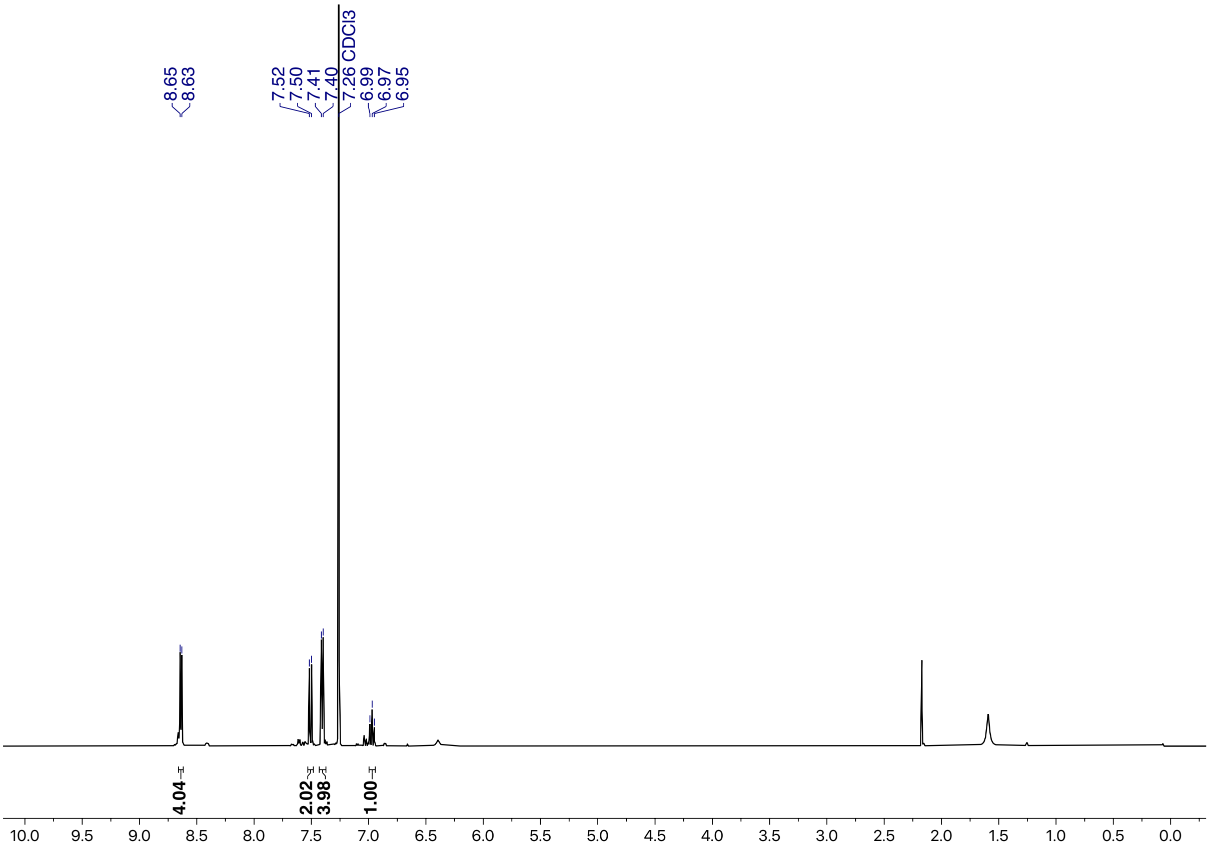

Figure S1. ^{1}H NMR ($CDCl_3$, 400 MHz, 298 K) of **2**.

Synthesis of 4-((2-(pyridin-4-yl)benzofuran-7-yl)ethynyl)pyridine ligand L

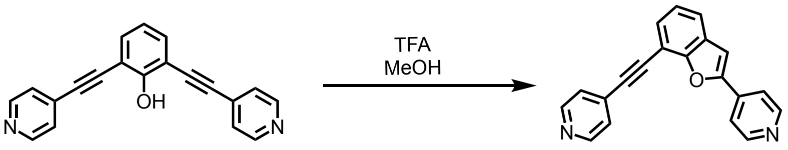

2 **L**

In a 50 mL round bottomed flask, compound S1 (100 mg, 0.34 mmol, 1 eq.,) was dissolved in methanol (15 mL). Trifluoroacetic acid (129 µL, 1.69 mmol, 5 eq.) was then added drop wise to this. The mixture solution was stirred at room temperature overnight. After the reaction, the mixture was concentrated under reduced pressure to remove MeOH and excess TFA. The resulting crude solid was washed with cold diethyl ether (3 × 5 mL) to remove residual TFA, and dried under high vacuum to afford ligand L as a white solid (97 mg, 97% yield).

^{1}H NMR (600 MHz, DMSO-d_6) δ 8.72 (ddd, J = 16.9, 4.4, 1.6 Hz, 4H), 7.95 – 7.91 (m, 2H), 7.88 (s, 2H), 7.87 (dd, J = 7.8, 1.2 Hz, 3H), 7.70 – 7.65 (m, 1H).

^{13}C NMR (151 MHz, DMSO-d_6) δ 155.63, 154.77, 151.52, 151.03, 137.52, 131.45, 130.27, 130.03, 126.36, 124.79, 124.45, 119.69, 118.26, 107.13, 106.42, 92.16, 88.34.

ESI-HRMS: m/z observed 610.1966 [$\mathbf{L}_2$ + NH_4]$^+$, calc. 610.2025. Mass Error: - 9.7 ppm.

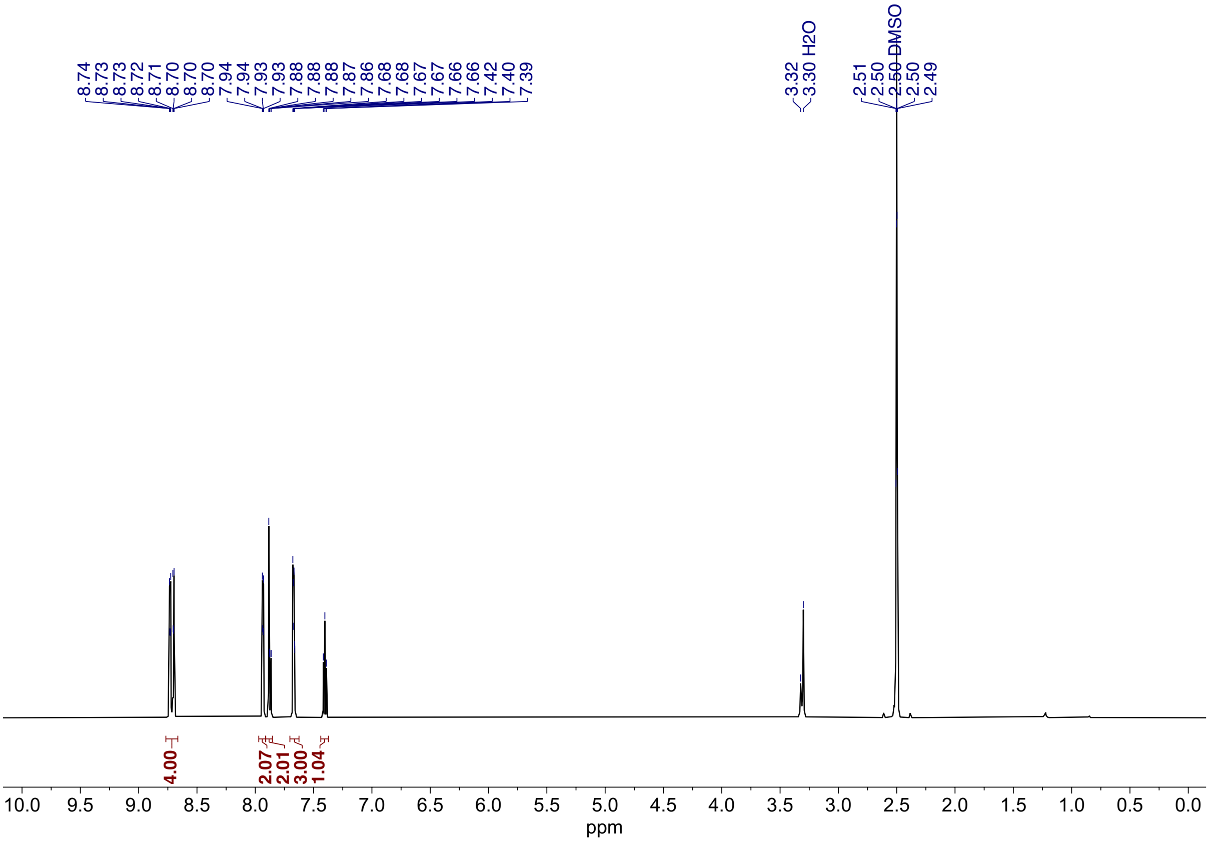

Figure S2. ^{1}H NMR (DMSO-d_6, 600 MHz, 298 K) of **L**.

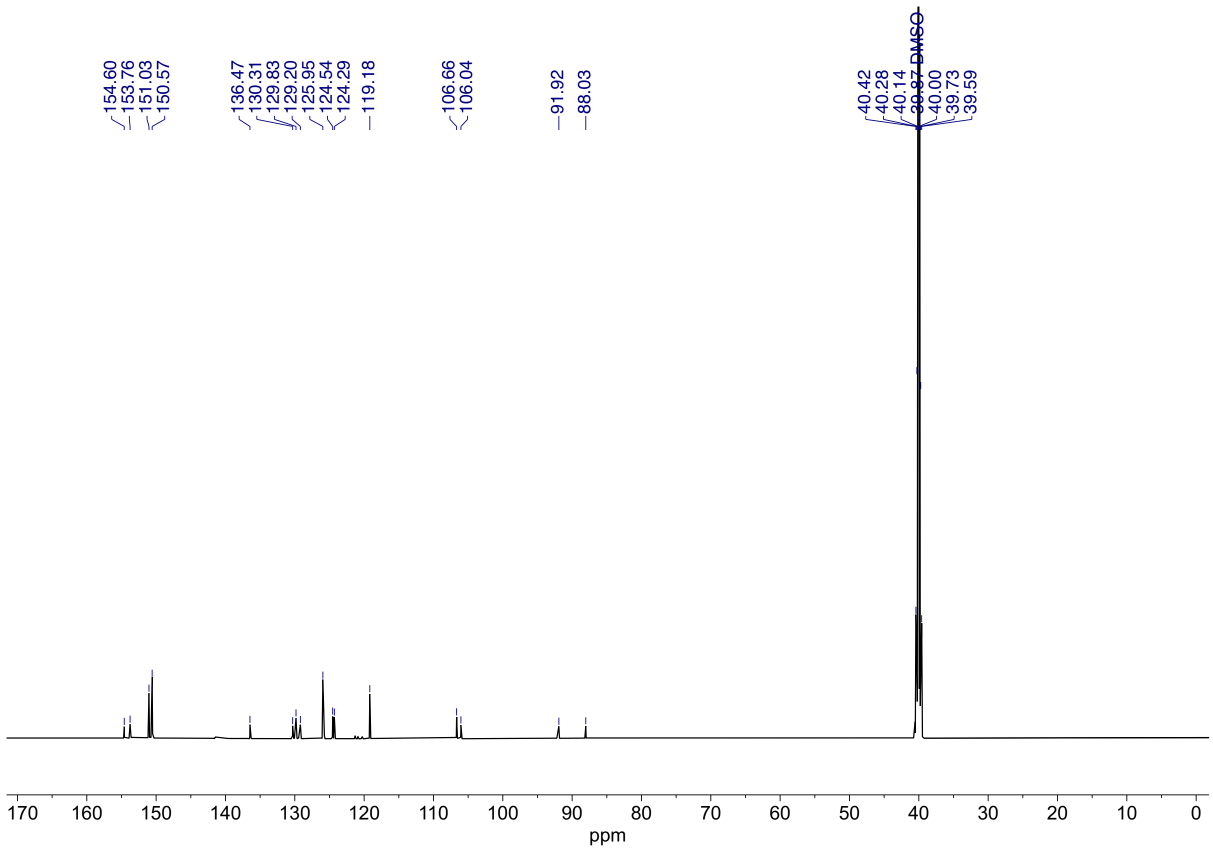

Figure S3. ^{13}C NMR (DMSO-d_6, 600 MHz, 298 K) of **L**.

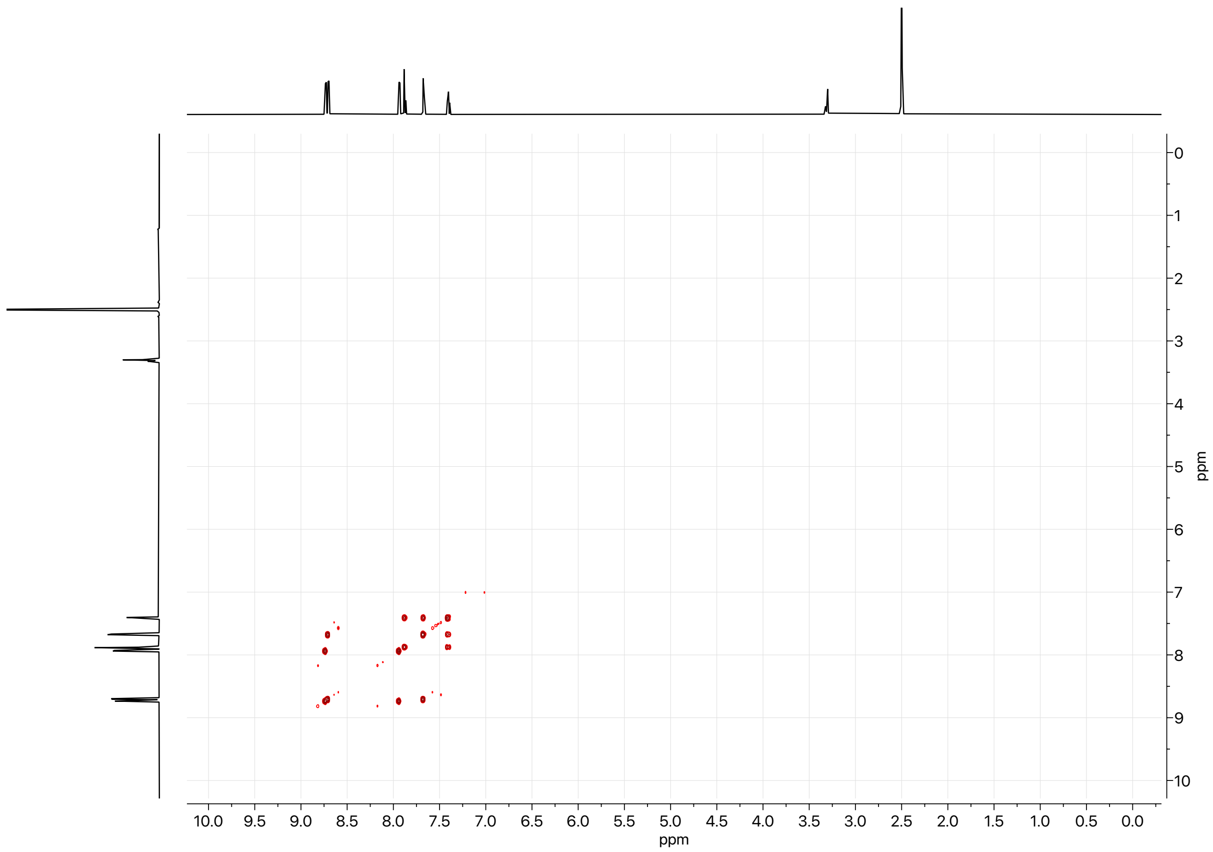

Figure S4. COSY NMR (DMSO-d_6, 600 MHz, 298 K) of **L**.

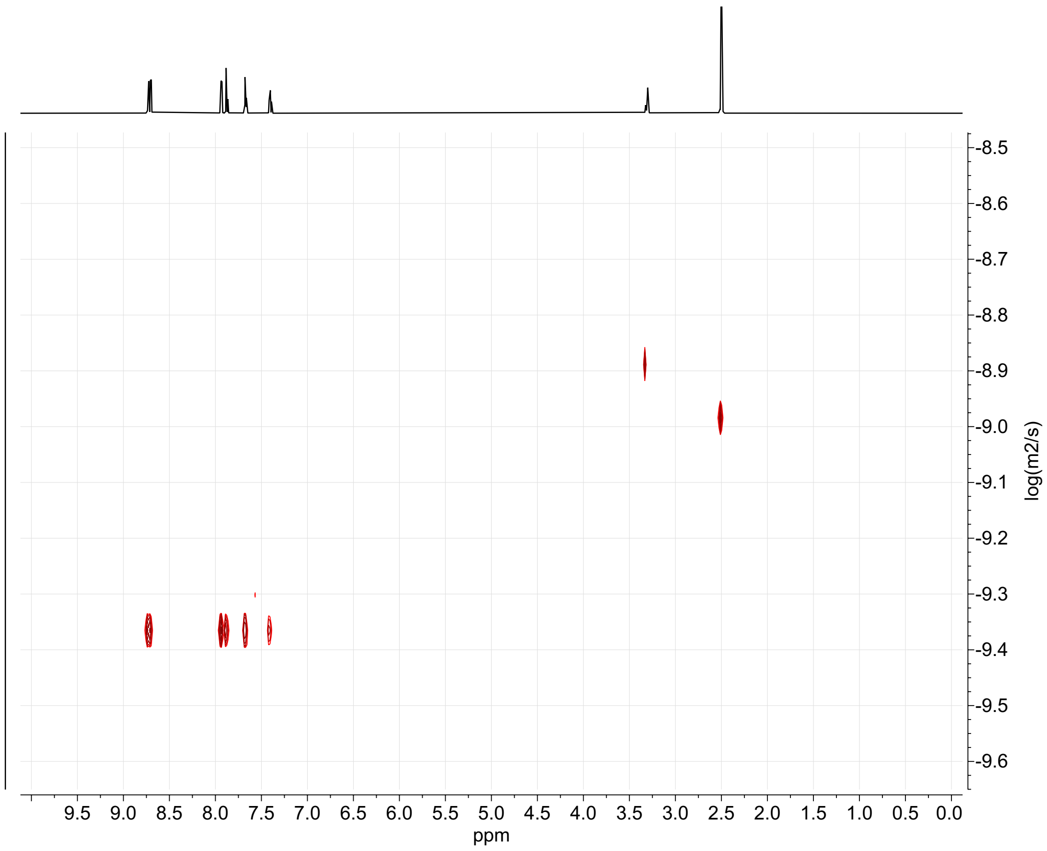

Figure S5. DOSY NMR (DMSO-d_6, 600 MHz, 298 K) of **L**. The diffusion coefficient of **L** was 4.3 × 10^{-10} m^2/s, giving a hydrodynamic radius of 2.3 Å.

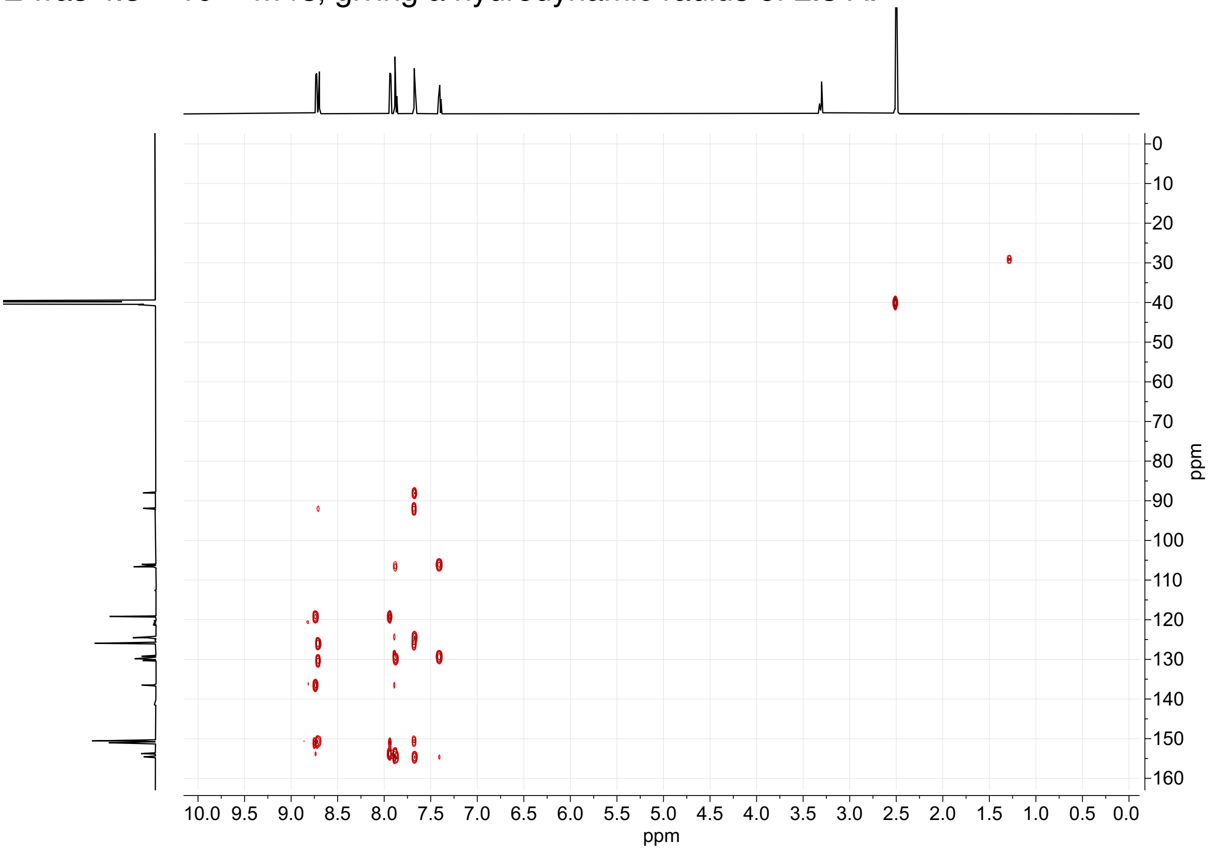

Figure S6. HMBC NMR (DMSO-d_6, 600 MHz, 298 K) of **L**.

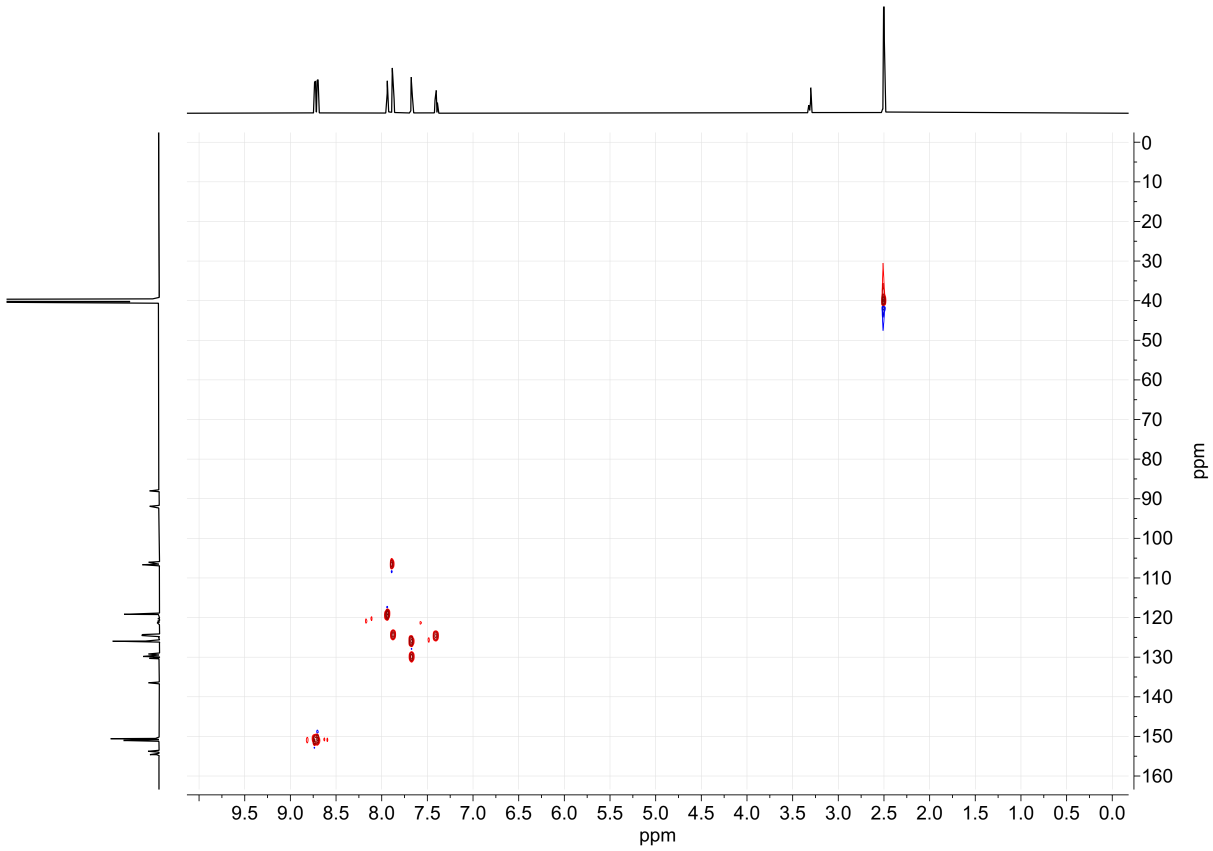

Figure S7. HSQC NMR (DMSO-d_6, 600 MHz, 298 K) of **L**.

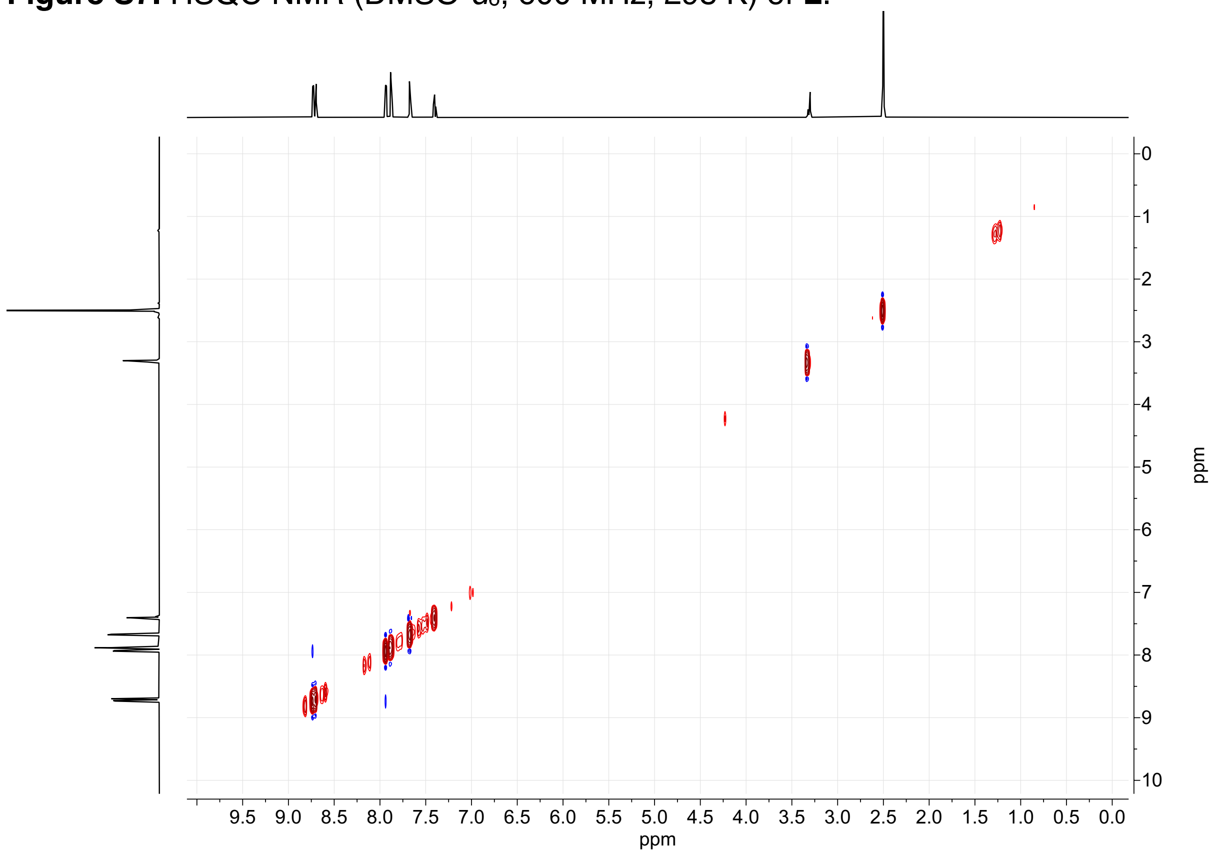

Figure S8. NOESY NMR (DMSO-d_6, 600 MHz, 298 K) of **L**.

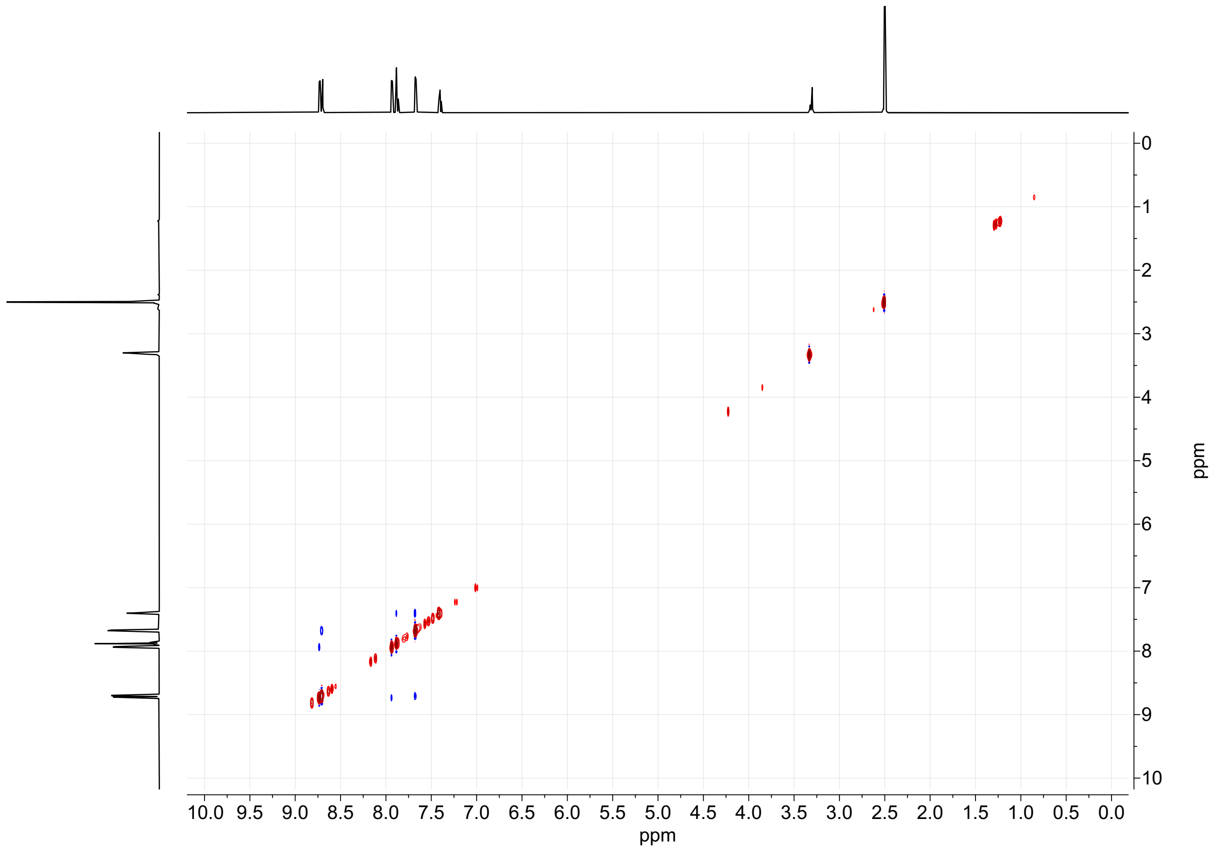

Figure S9. ROESY NMR (DMSO-d_6, 600 MHz, 298 K) of **L**.

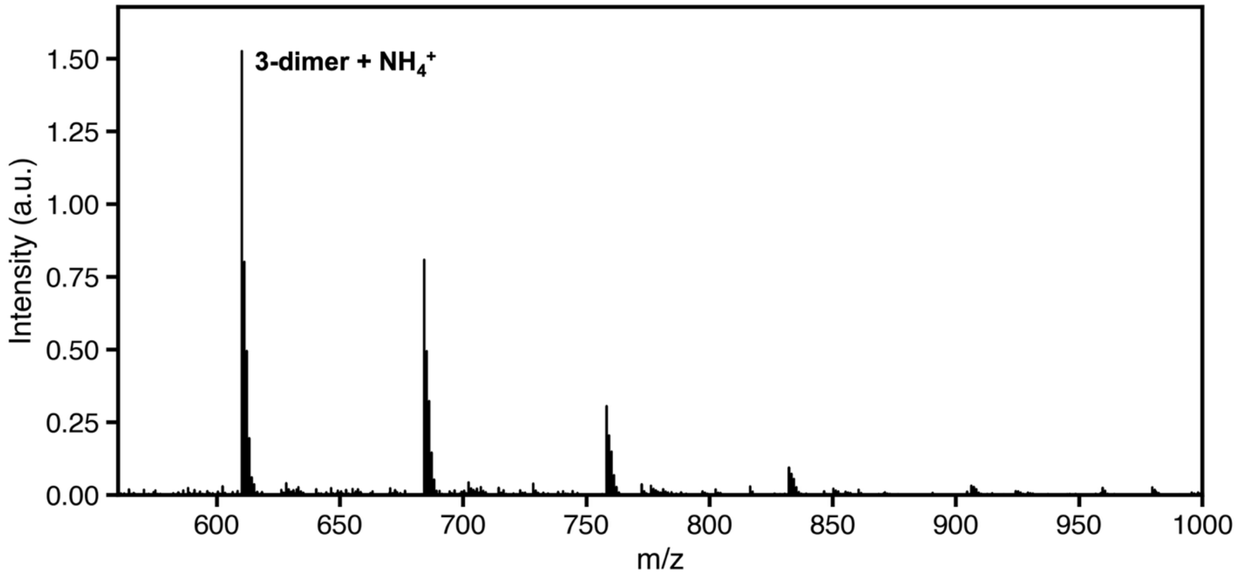

Figure S10. ESI-MS (positive-ion mode, CH_3CN) of ligand **L**.

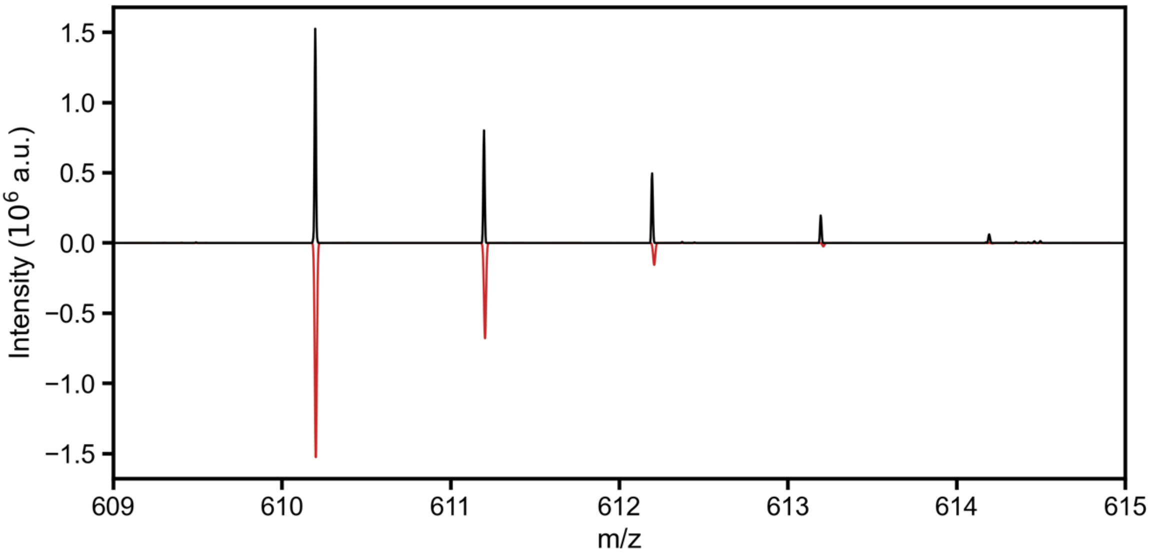

Figure S11. HRMS experimental (top) and calculated (bottom) isotopic distribution of [**L**$_2$ + NH_4]$^+$.

Synthesis of Cage $Pd_4L_8(BF_4)_8$ and $Pd_4L_8(OTf)_8$

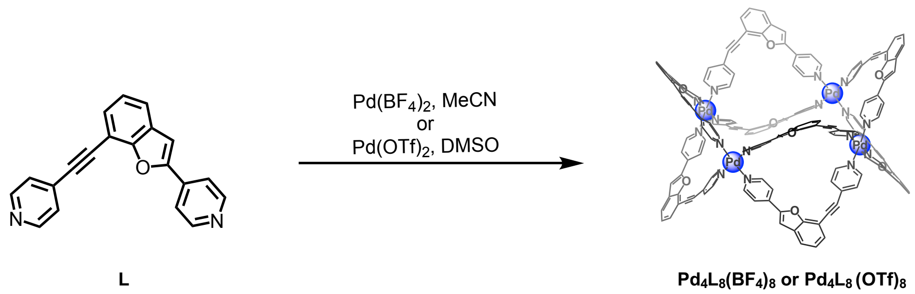

Condition 1 ($Pd_4L_8(BF_4)_8$): In a 4 mL vial, the solution of ligand **L** (1.00 mg, 3.37 µmol, 2 eq.) and $Pd(MeCN)_4(BF_4)_2$ (0.75 mg, 1.69 µmol, 1 eq.) was dissolved in 0.6 ml d_3-MeCN, sonicated briefly, then heated at 50 °C for 4 h.

Condition 2 ($Pd_4L_8(OTf)_8$): In a 4 mL vial, the solution of ligand **L** (1.00 mg, 3.37 µmol, 2 eq.) and $Pd(MeCN)_4(OTf)_2$ (0.96 mg, 1.69 µmol, 1 eq.) was dissolved in 0.6 mL DMS-d_6, sonicated briefly, then heated at 50 °C for 4 h.

The quantitative formation of $Pd_4L_8(BF_4)_8$ and $Pd_4L_8(OTf)_8$ was confirmed by ^{1}H NMR. In both cases, broad spectra indicated the likely presence of multiple isomeric species without a dominant isomer, and the mixtures were used without further purification.

^{1}H NMR (600 MHz, DMSO-d_6) δ 9.46 (d, J = 39.3 Hz, 4H), 8.33 (s, 2H), 8.16 – 8.02 (m, 3H), 7.95 (d, J = 6.7 Hz, 1H), 7.70 (s, 1H), 7.43 (dt, J = 8.4, 4.4 Hz, 1H).

^{13}C NMR (151 MHz, DMSO-d_6) δ 155.05, 151.90, 151.46, 139.87, 134.53, 131.90, 129.24, 128.94, 126.08, 125.09, 122.30, 118.53, 110.62, 110.51, 105.27, 92.67, 90.48.

^{11}B NMR (128 MHz, DMSO-d_6) δ -0.99.

^{19}F NMR (376 MHz, DMSO-d_6) δ -147.90, -147.95, -147.96.

ESI-HRMS: m/z observed 494.8964 [**4**$(BF_4)_2]^{6+}$, calc. 494.8955.

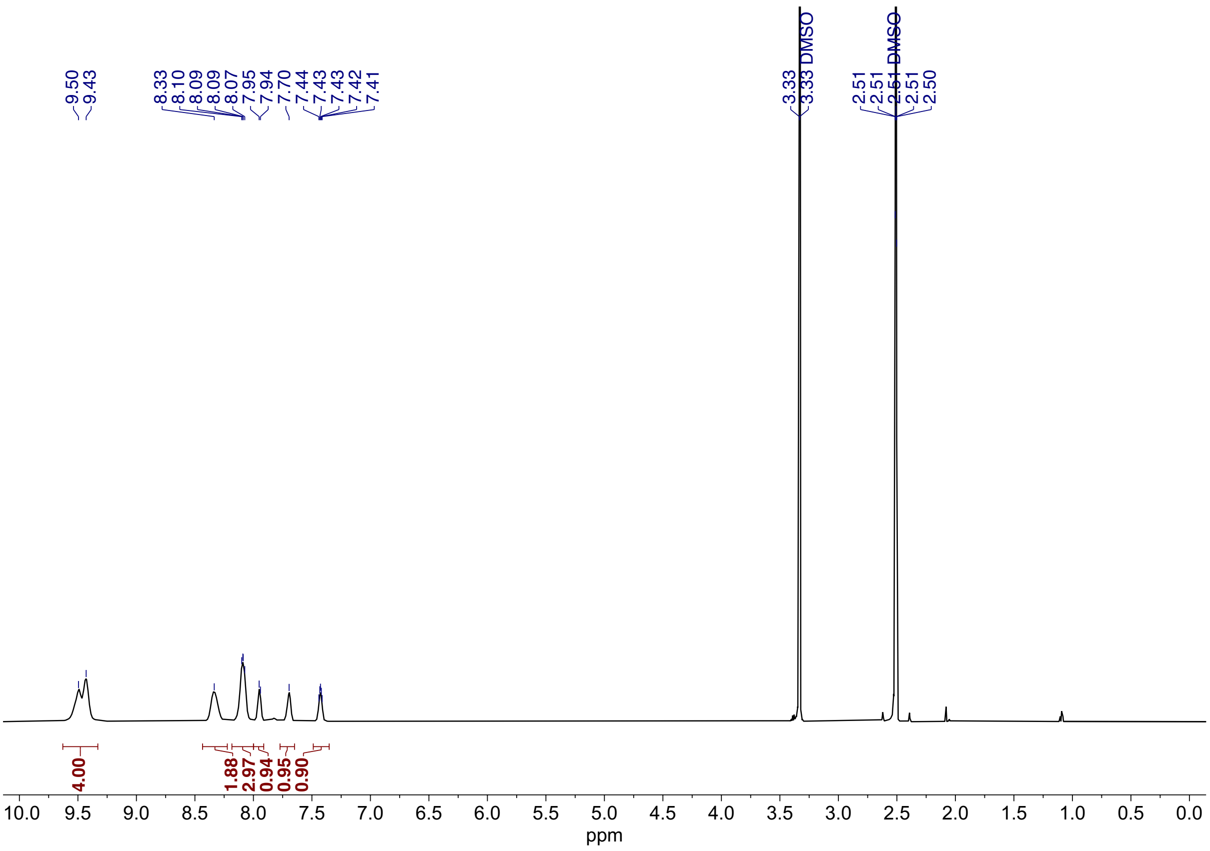

Figure S12. ^{1}H NMR (DMSO-*d*$_6$, 600 MHz, 298 K) of $Pd_4L_8(BF_4)_8$.

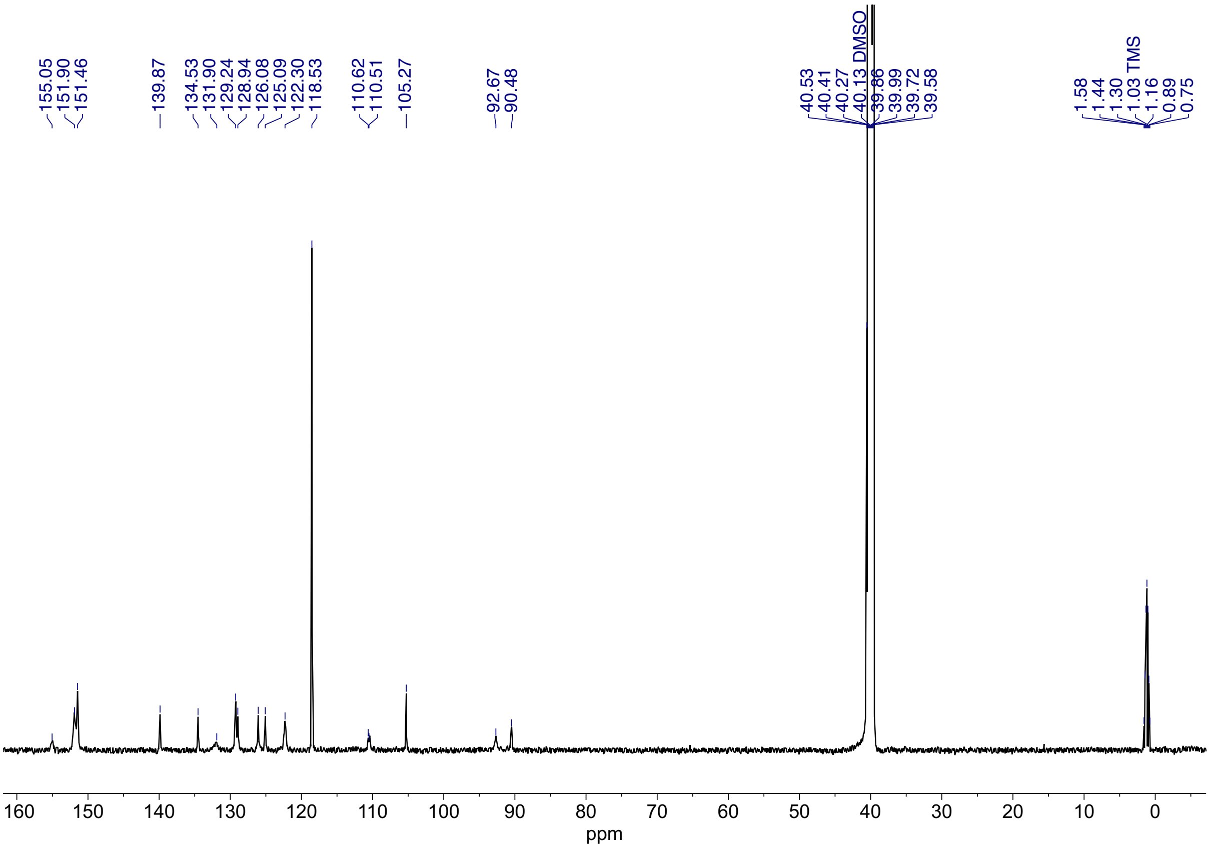

Figure S13. ^{13}C NMR (DMSO-*d*$_6$, 600 MHz, 298 K) of $Pd_4L_8(BF_4)_8$.

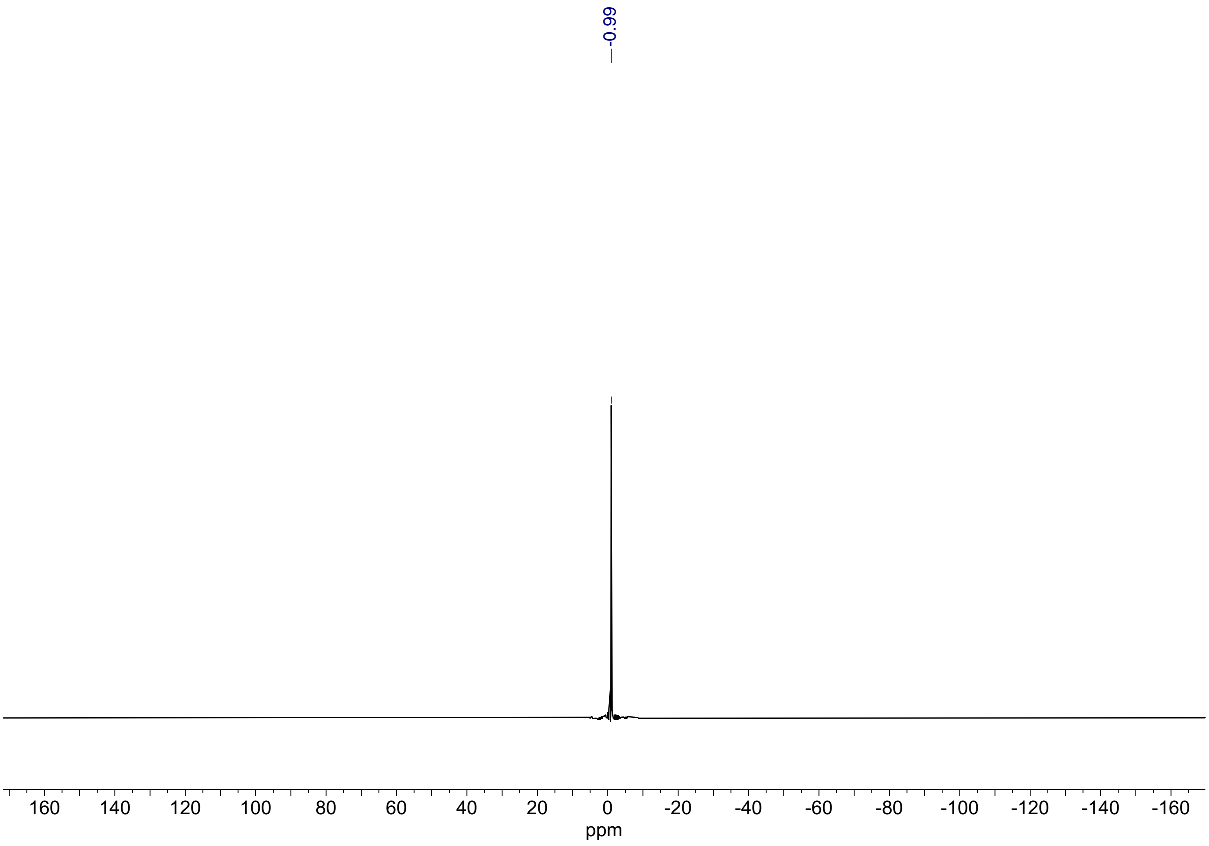

Figure S14. ^{11}B NMR (DMSO-d_6, 600 MHz, 298 K) of $Pd_4L_8(BF_4)_8$.

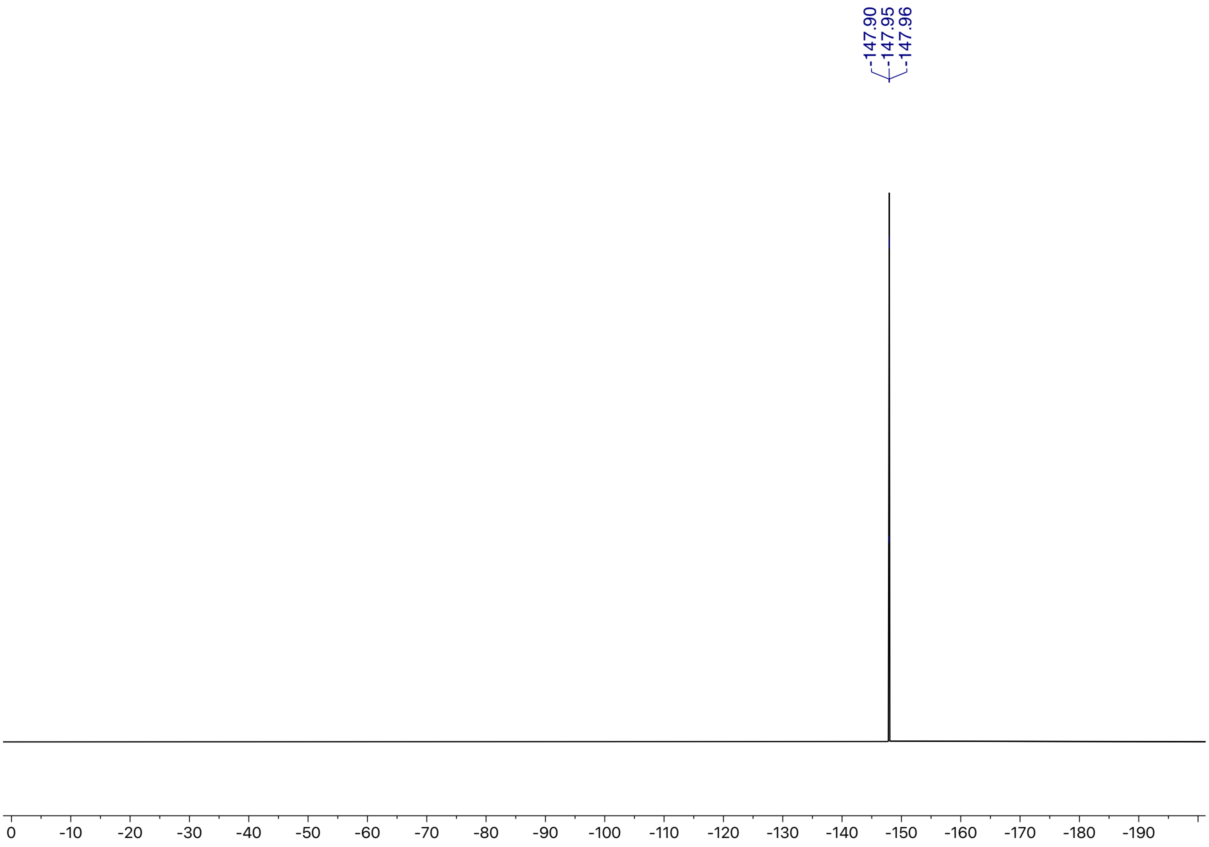

Figure S15. ^{19}F NMR (DMSO-d_6, 600 MHz, 298 K) of $Pd_4L_8(BF_4)_8$.

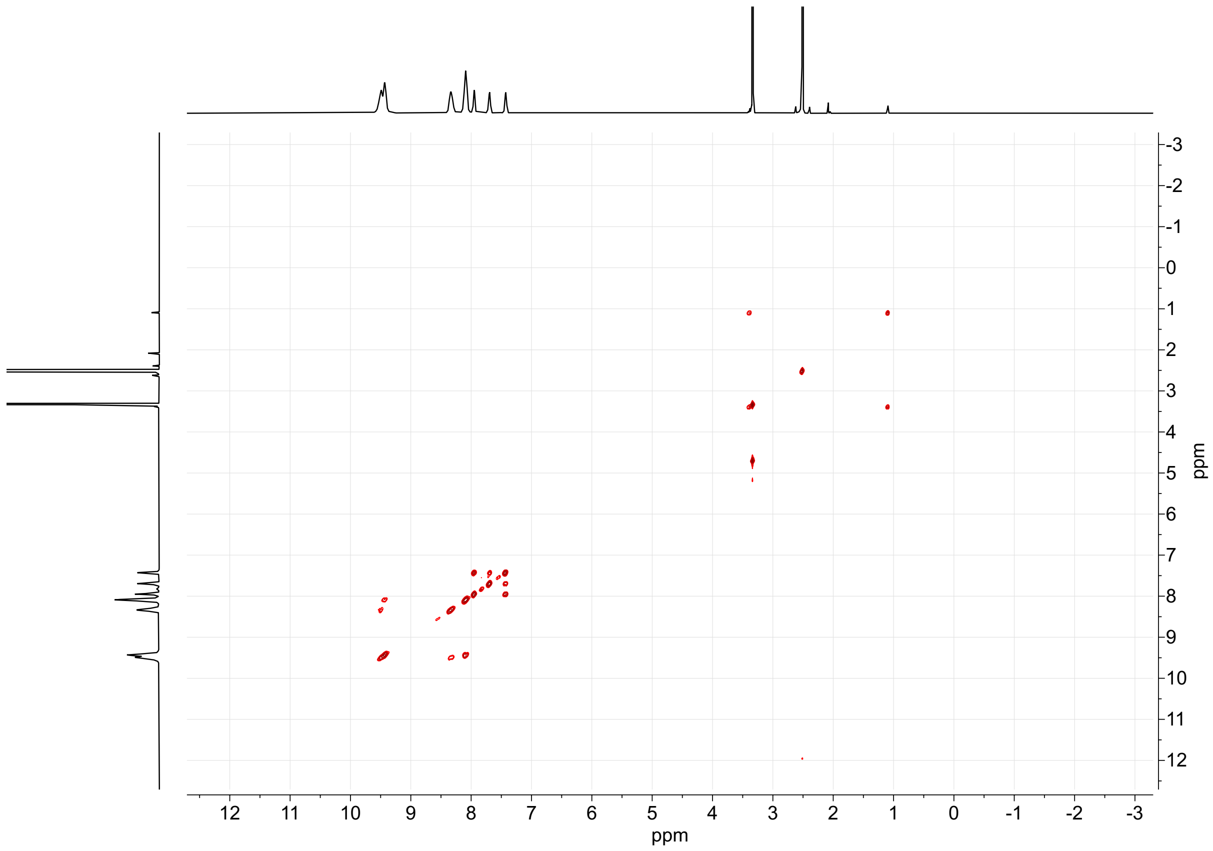

Figure S16. COSY NMR (DMSO-d_6, 600 MHz, 298 K) of $Pd_4L_8(BF_4)_8$.

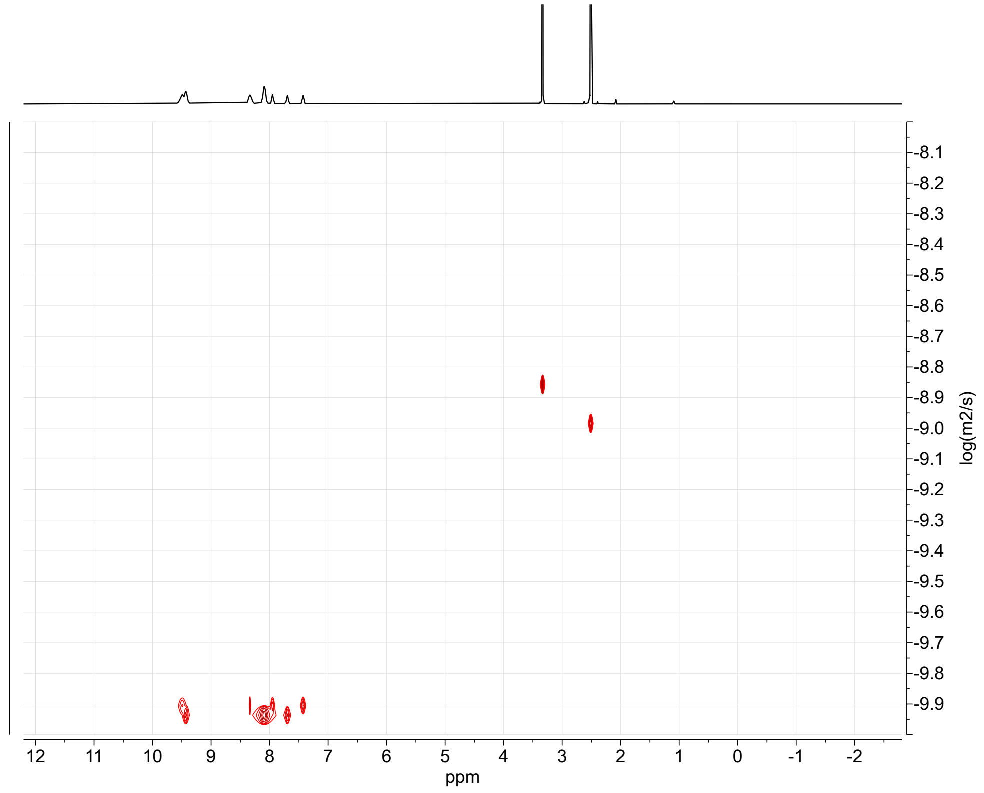

Figure S17. DOSY NMR (DMSO-d_6, 600 MHz, 298 K) of $Pd_4L_8(BF_4)_8$. The diffusion coefficient of $Pd_4L_8(BF_4)_8$ was 9.3 × 10^{-10} m2/s, giving a hydrodynamic radius of 9.6 Å.

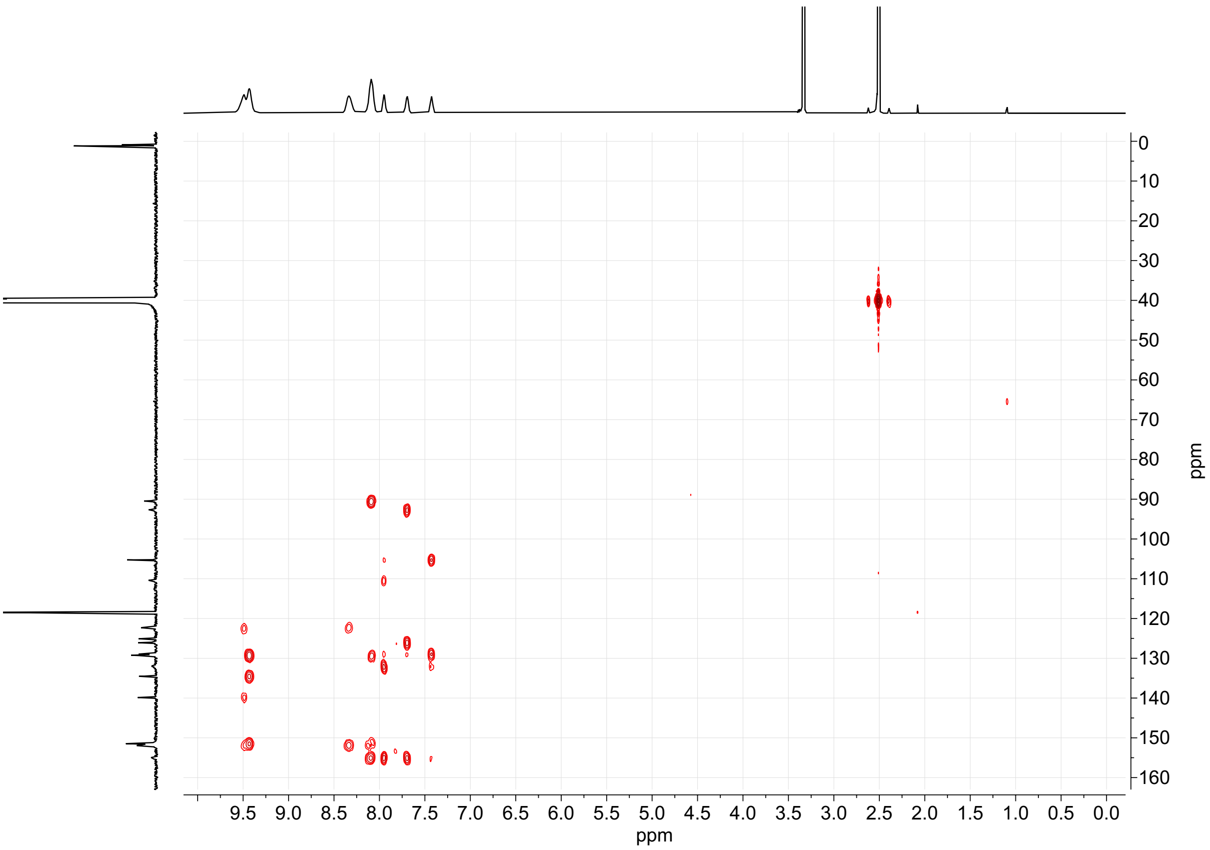

Figure S18. HMBC NMR (DMSO-*d*6, 600 MHz, 298 K) of $Pd_4L_8(BF_4)_8$.

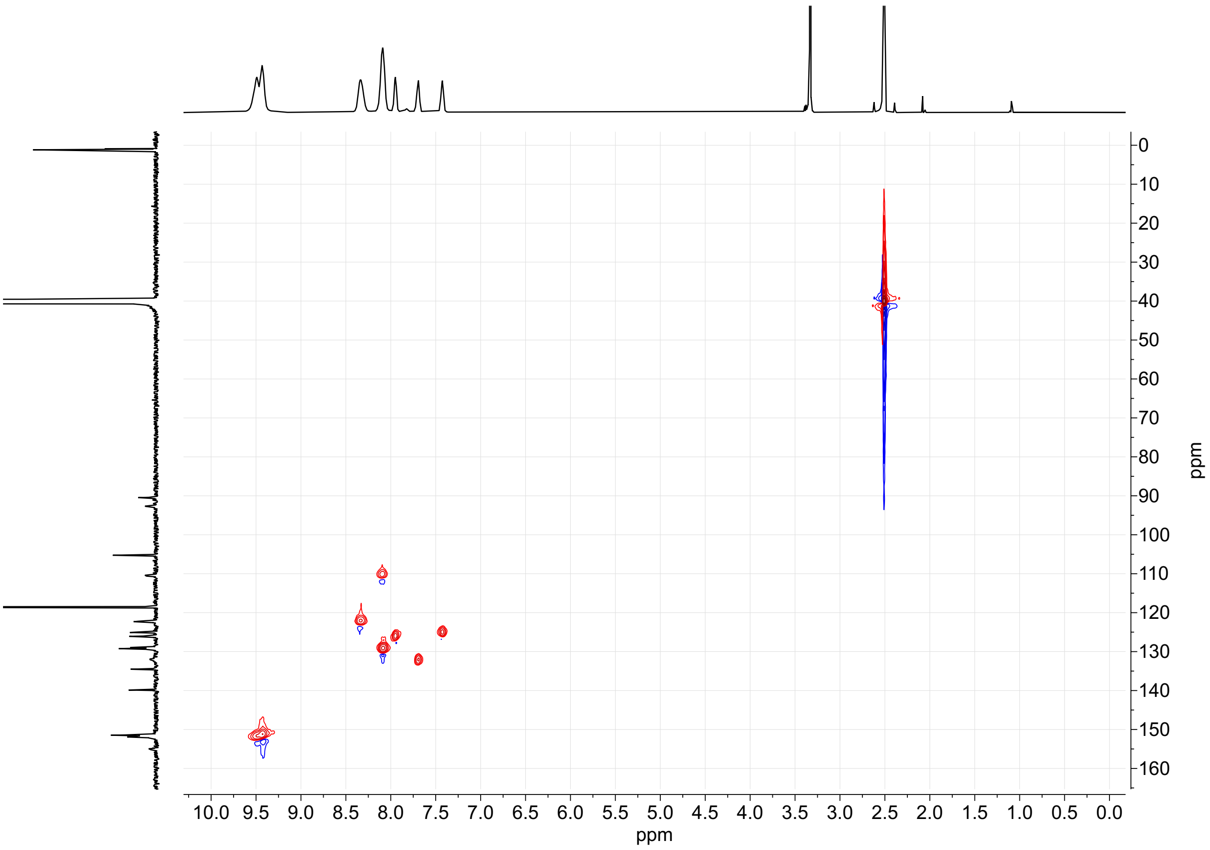

Figure S19. HSQC NMR (DMSO-*d*6, 600 MHz, 298 K) of $Pd_4L_8(BF_4)_8$.

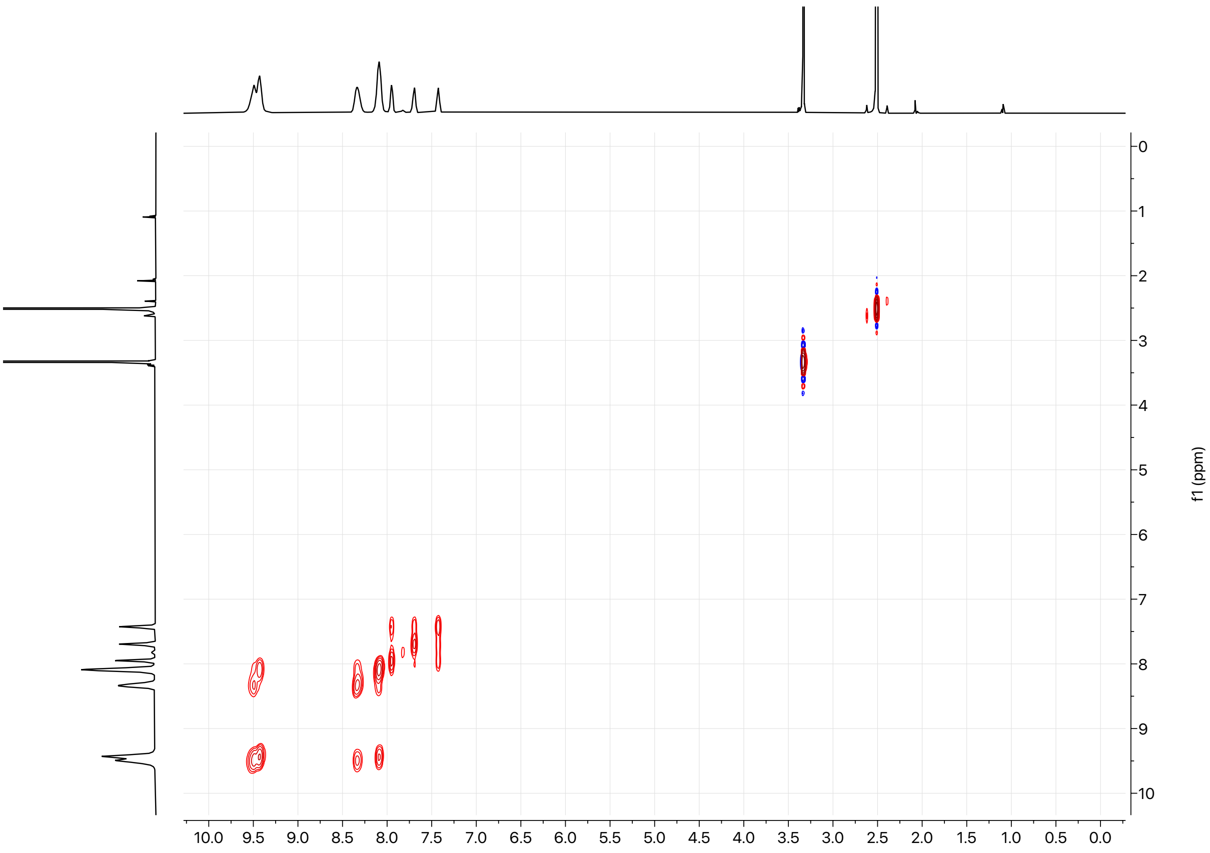

Figure S20. NOESY NMR (DMSO-d_6, 600 MHz, 298 K) of $Pd_4L_8(BF_4)_8$.

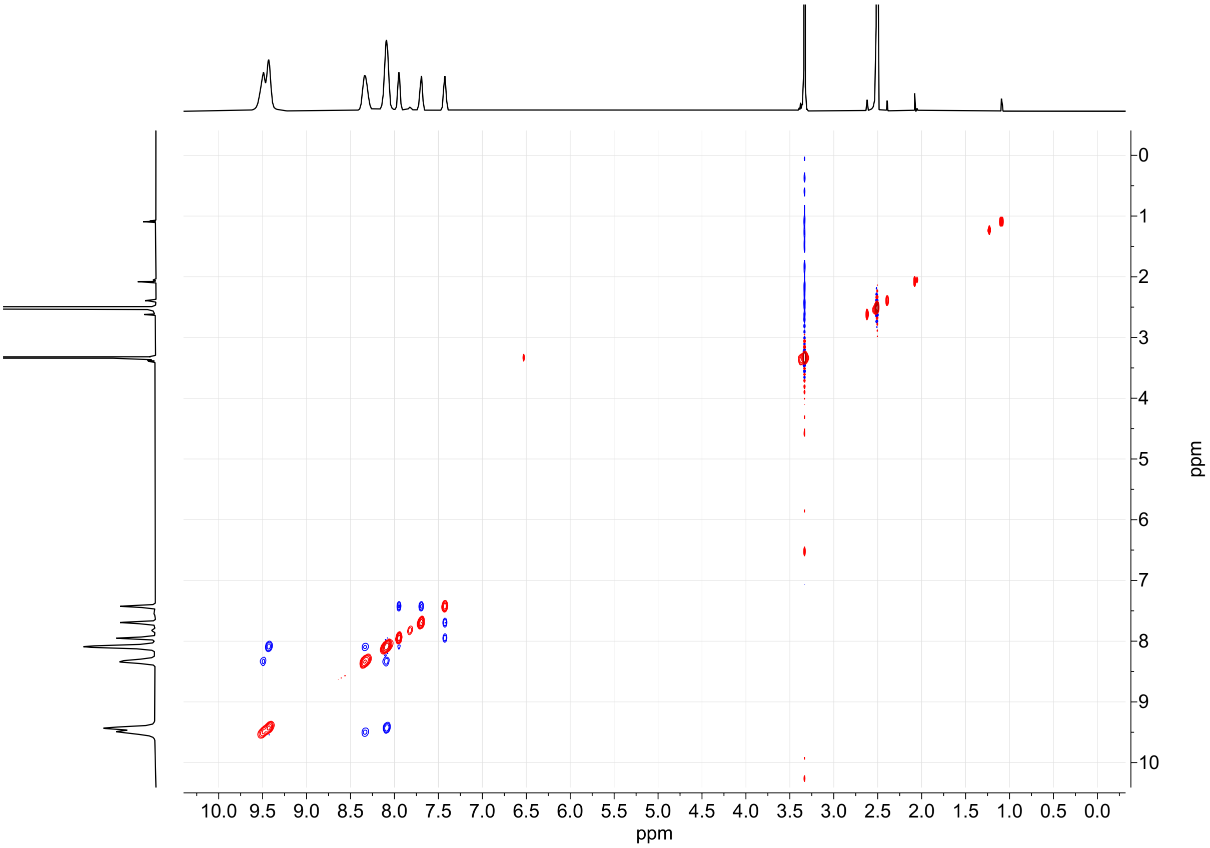

Figure S21. ROESY NMR (DMSO-d_6, 600 MHz, 298 K) of $Pd_4L_8(BF_4)_8$.

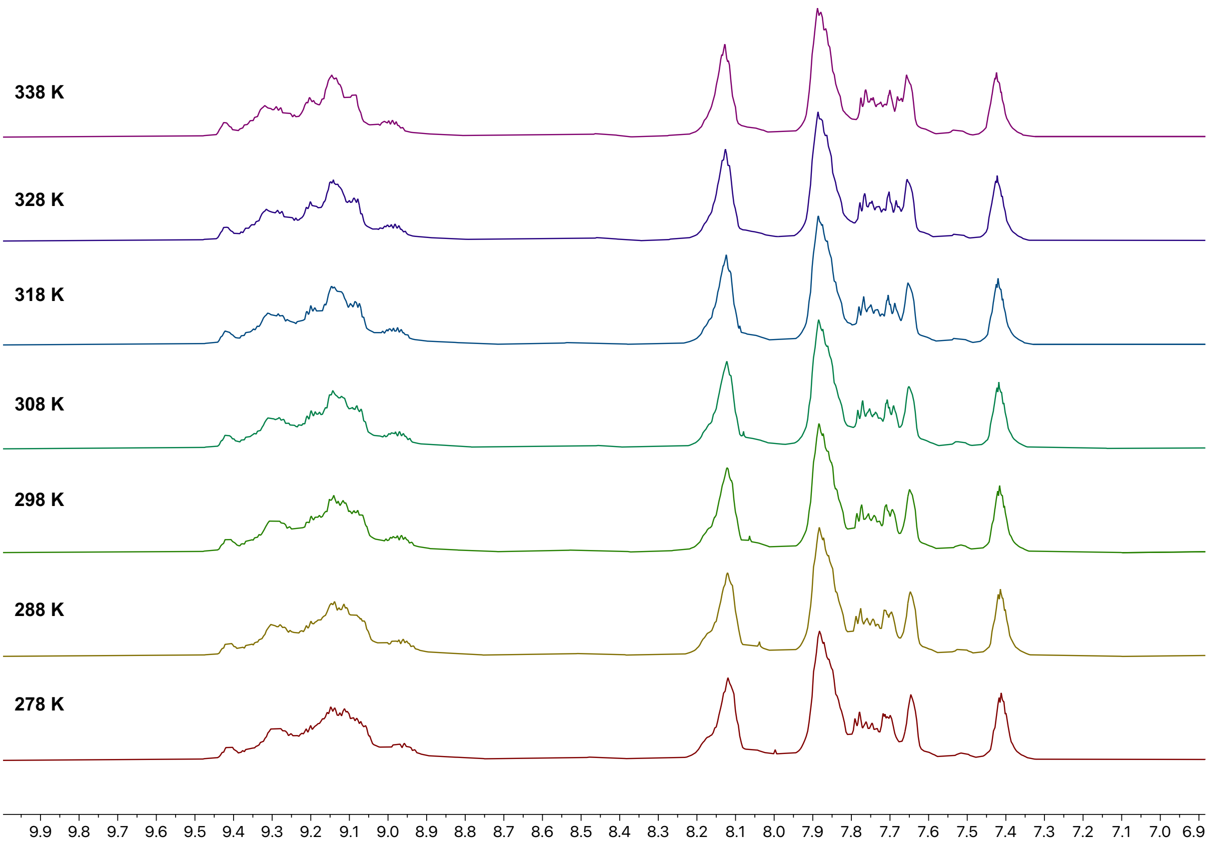

Figure S22. VT NMR (d_3-MeCN, 600 MHz, 298 K) of $Pd_4L_8(BF_4)_8$ from 278K to 338K.

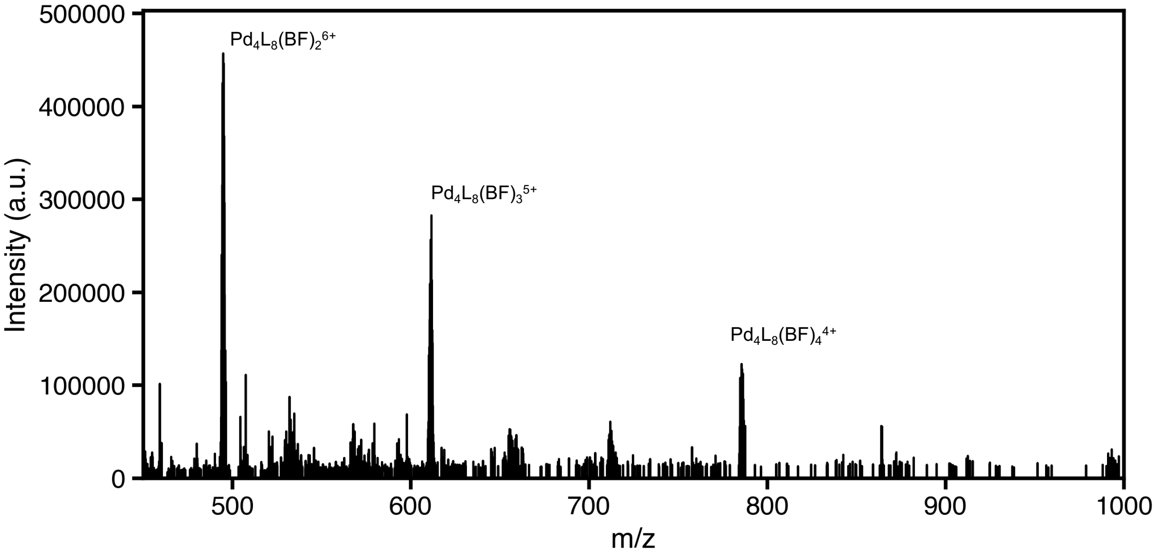

Figure S23. ESI-MS (positive-ion mode, CH_3CN) of $Pd_4L_8(BF_4)_8$.

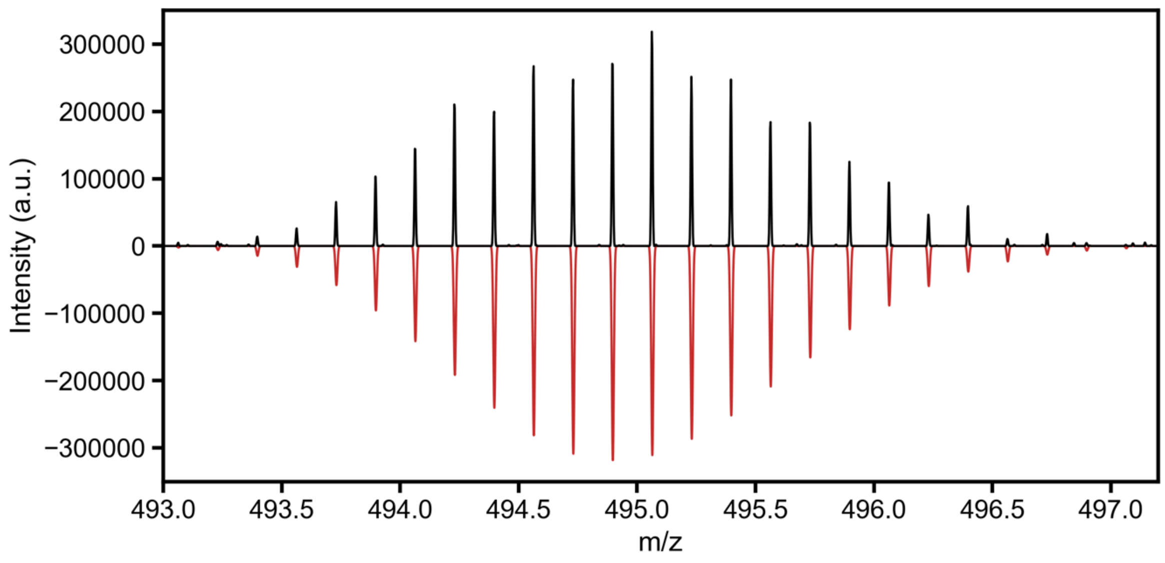

Figure S24. HRMS experimental (top) and calculated (bottom) isotopic distribution of $[Pd_4L_8(BF_4)_2]^{6+}$.

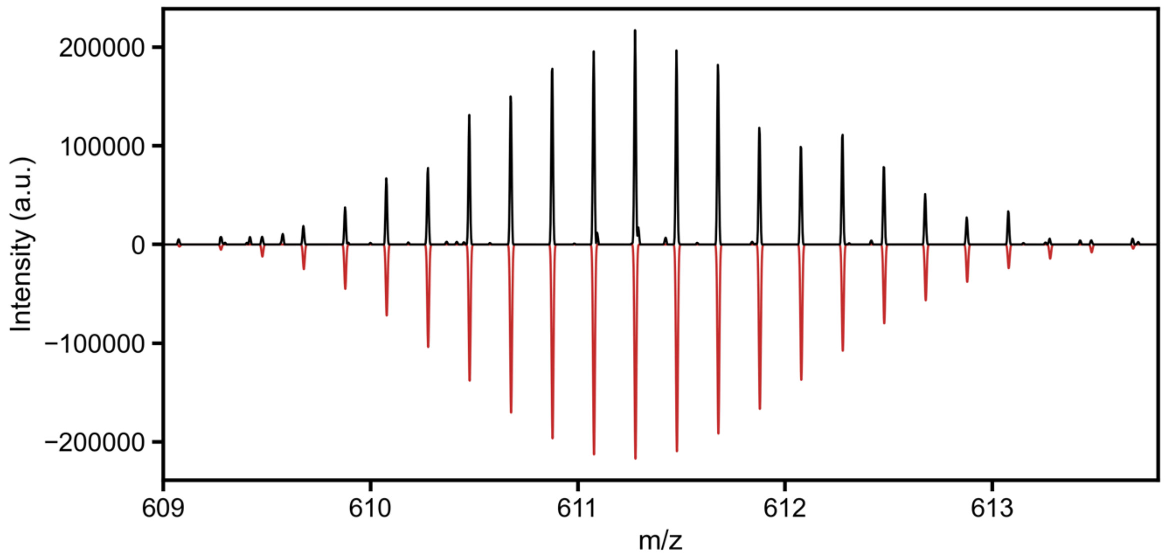

Figure S25. HRMS experimental (top) and calculated (bottom) isotopic distribution of $[Pd_4L_8(BF_4)_3]^{5+}$.

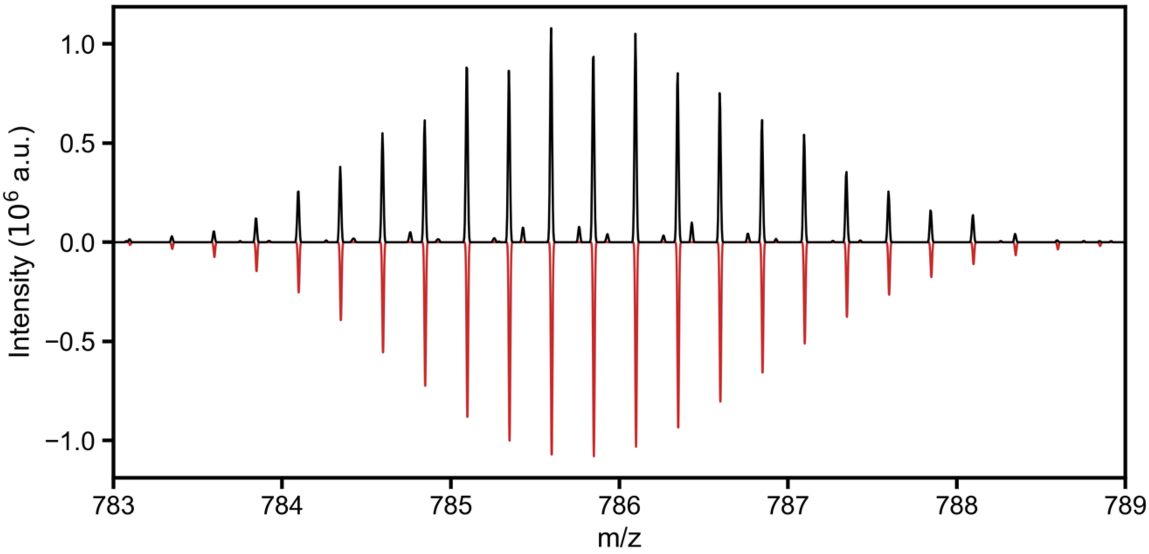

Figure S26. HRMS experimental (top) and calculated (bottom) isotopic distribution of $[Pd_4L_8(BF_4)_4]^{4+}$.

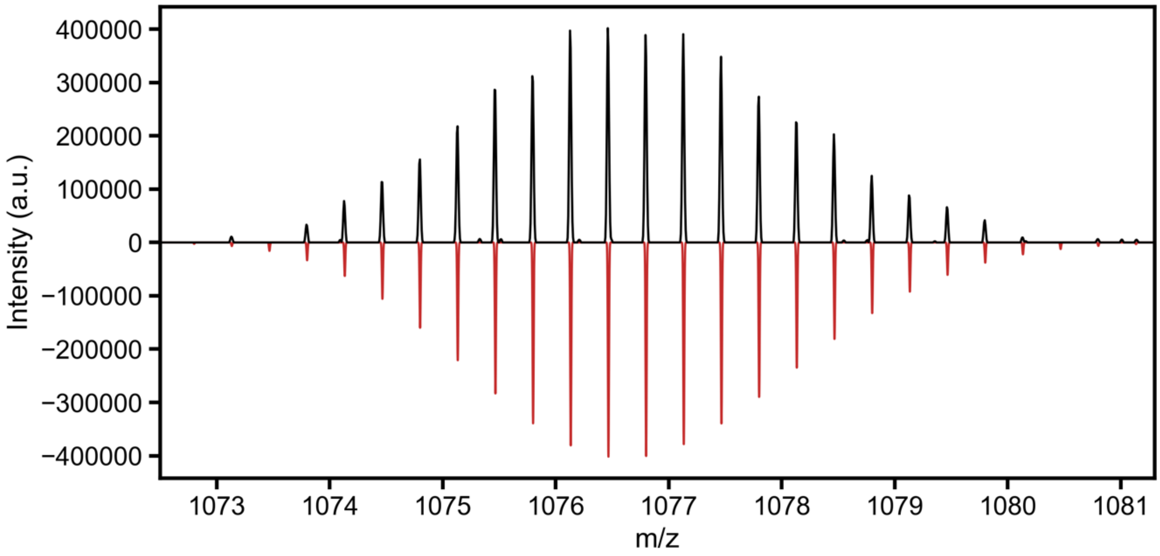

Figure S27. HRMS experimental (top) and calculated (bottom) isotopic distribution of $[Pd_4L_8(BF_4)_5]^{3+}$.

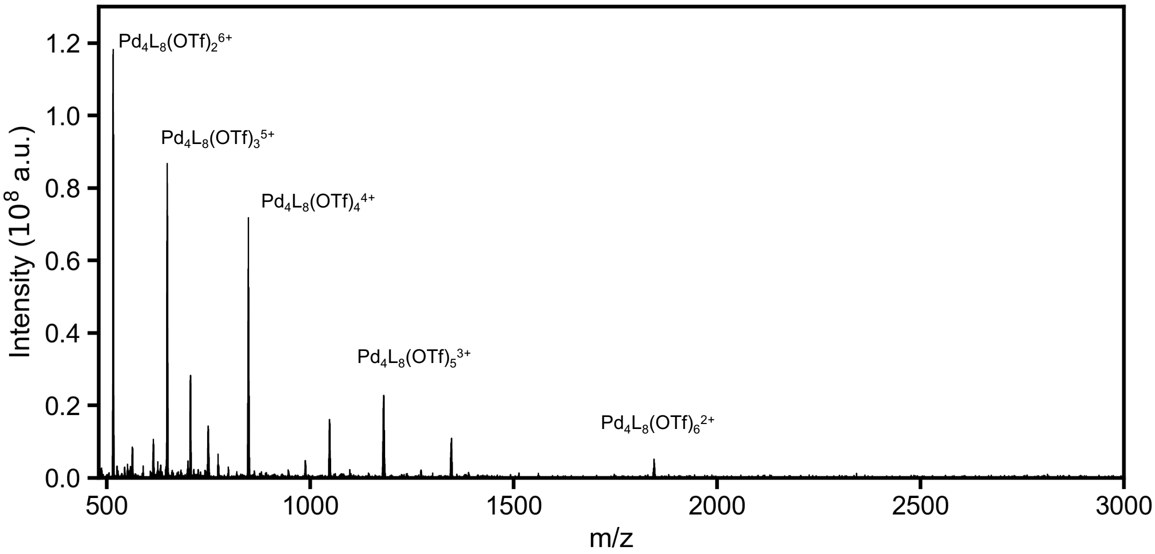

Figure S28. ESI-MS (positive-ion mode, CH_3CN) of $Pd_4L_8(OTf)_8$.

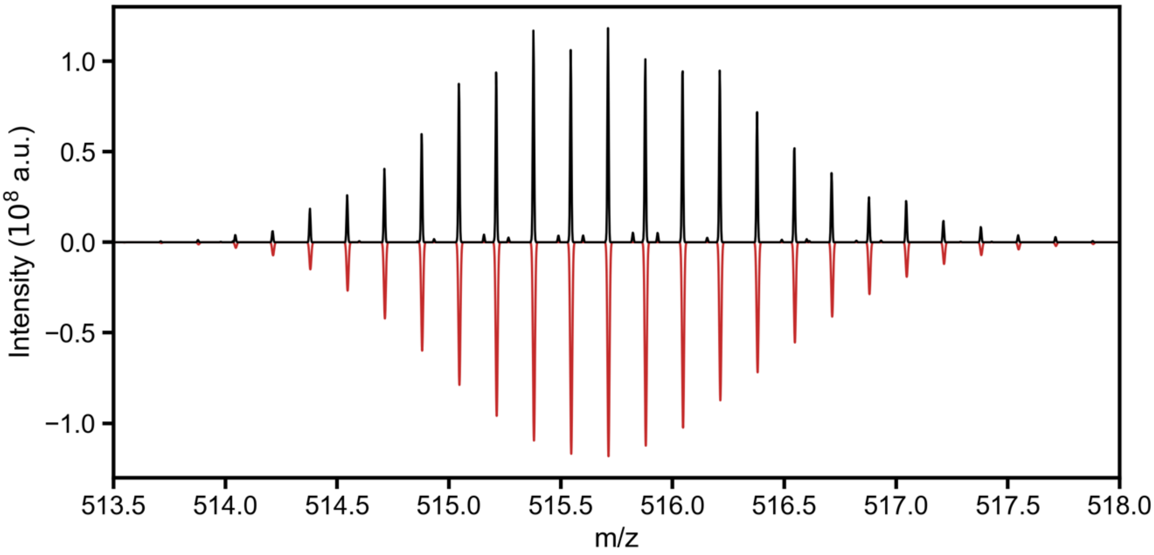

Figure S29. HRMS experimental (top) and calculated (bottom) isotopic distribution of $[Pd_4L_8(OTf)_2]^{6+}$.

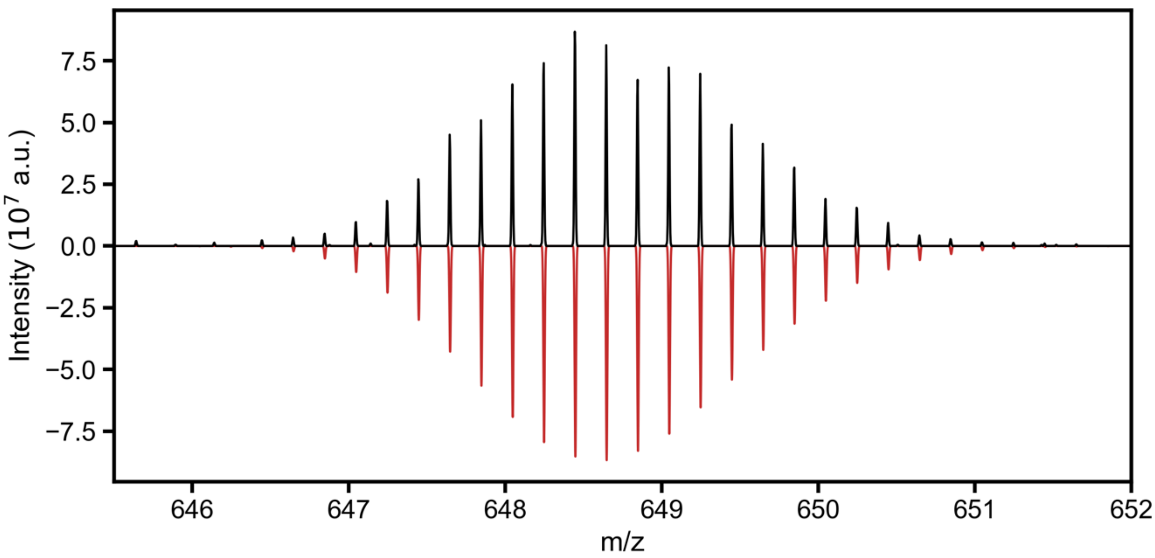

Figure S30. HRMS experimental (top) and calculated (bottom) isotopic distribution of $[Pd_4L_8(OTf)_3]^{5+}$.

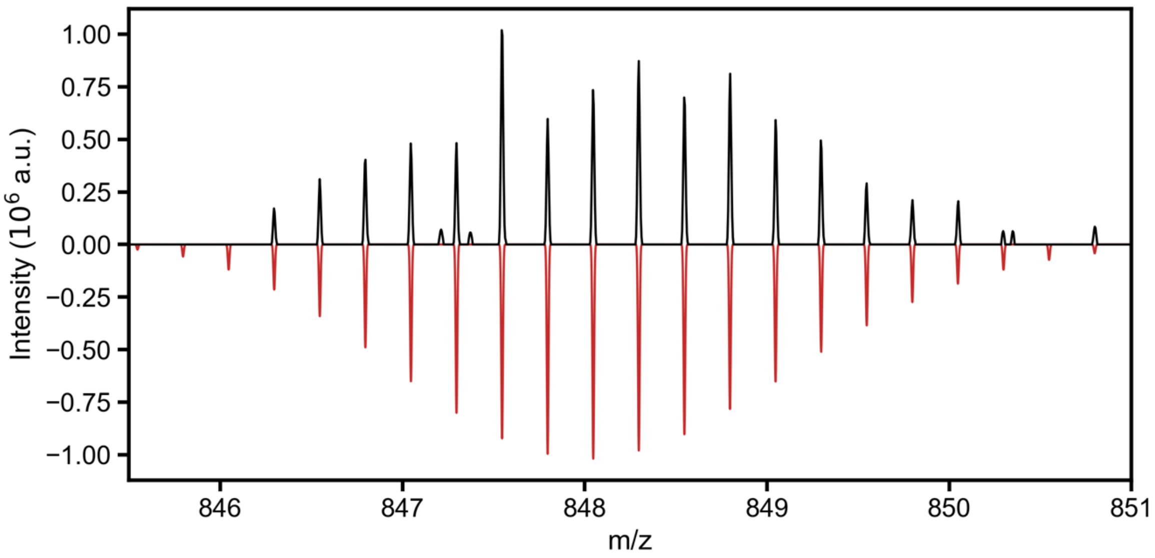

Figure S31. HRMS experimental (top) and calculated (bottom) isotopic distribution of $[Pd_4L_8(OTf)_4]^{4+}$.

Synthesis of Cage $Pd_6L_{12}(NTf_2)_{12}$ and $Pd_6L_{12}(OTf)_{12}$

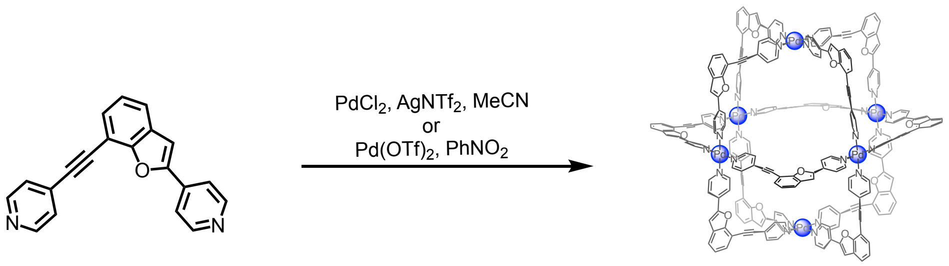

L

$Pd_6L_{12}(NTf_2)_{12}$ or $Pd_6L_{12}(OTf)_{12}$

Condition 1 ($Pd_6L_{12}(NTf_2)_{12}$): In a 4 mL vial, $AgNTf_2$ (1.31 mg, 3.37 µmol, 2 eq.) was dissolved in 0.3 mL *d*3-MeCN, and then added to the $PdCl_2$ (0.30 mg, 1.69 µmol, 1 eq.), the mixture was sonicated and stirred to mix well, and reacted at room temperature for 15 mins. Ligand **L** (1.00 mg, 3.37 µmol, 2 eq.) was dissolved in 0.3 mL *d*3-MeCN in another vial. Two solutions were then mixed and heated at 50 ^oC for 4 h.

Condition 2 ($Pd_6L_{12}(OTf)_{12}$): In a 4 mL vial, the solution of ligand **L** (1 mg, 3.37 µmol, 2 eq.) and $Pd(MeCN)_4(OTf)_2$ (0.96 mg, 1.69 µmol, 1 eq.) were dissolved in 0.6 mL nitrobenzene and well mixed, then heated at 50 ^oC for 4 h.

The quantitative formation of $Pd_6L_{12}(NTf_2)_{12}$ and $Pd_6L_{12}(OTf)_{12}$ was confirmed by ^1H NMR. In both cases, broad spectra indicated the likely presence of multiple isomeric species without a dominant isomer, and the mixtures were used without further purification.

^1H NMR (600 MHz, DMSO-*d*6) δ 9.23 – 8.96 (m, 4H), 8.10 (d, *J* = 7.5 Hz, 2H), 7.95 – 7.65 (m, 5H), 7.46 – 7.34 (m, 1H).

^{13}C NMR (151 MHz, DMSO-*d*6) δ 151.35, 150.91, 140.52, 135.52, 135.50, 132.35, 128.92, 125.50, 124.53, 123.25, 122.04, 121.12, 118.99, 117.34, 109.98, 105.36, 93.05, 89.51.

^{19}F NMR (376 MHz, DMSO-*d*6) δ -78.66.

ESI-HRMS: m/z observed 1230.9919 [**5**$(NTf_2)_7]^{5+}$, calc. 1230.9981.

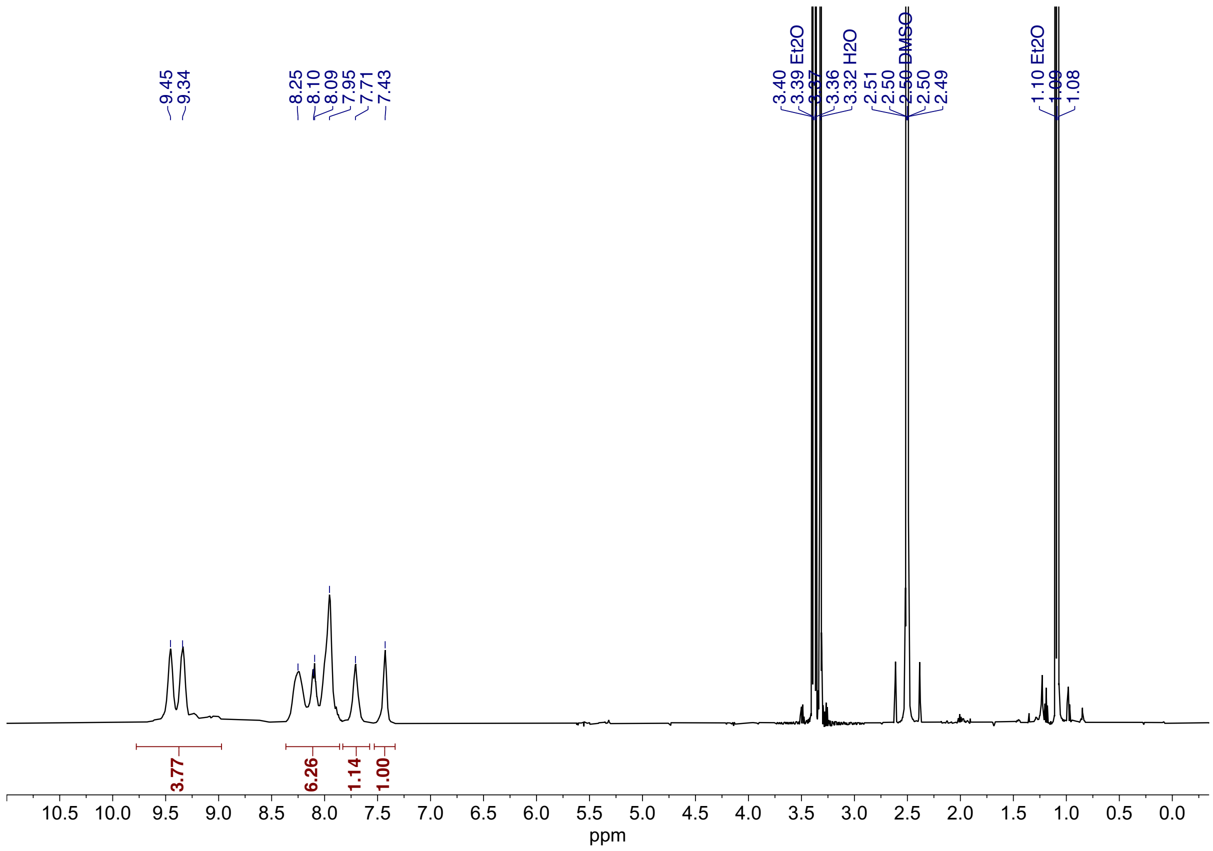

Figure S32. ^{1}H NMR (DMSO-d_6, 600 MHz, 298 K) of $Pd_6L_{12}(NTf_2)_{12}$.

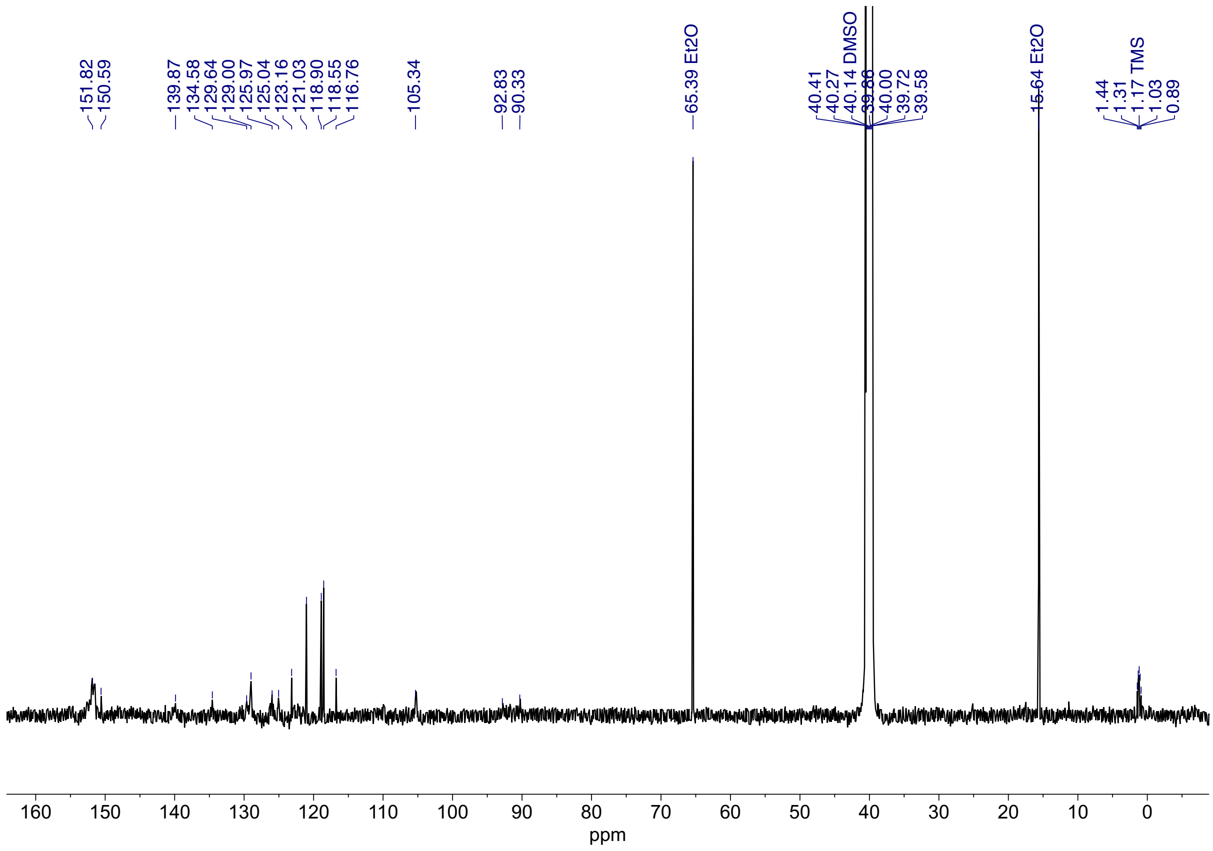

Figure S33. ^{13}C NMR (DMSO-d_6, 600 MHz, 298 K) of $Pd_6L_{12}(NTf_2)_{12}$.

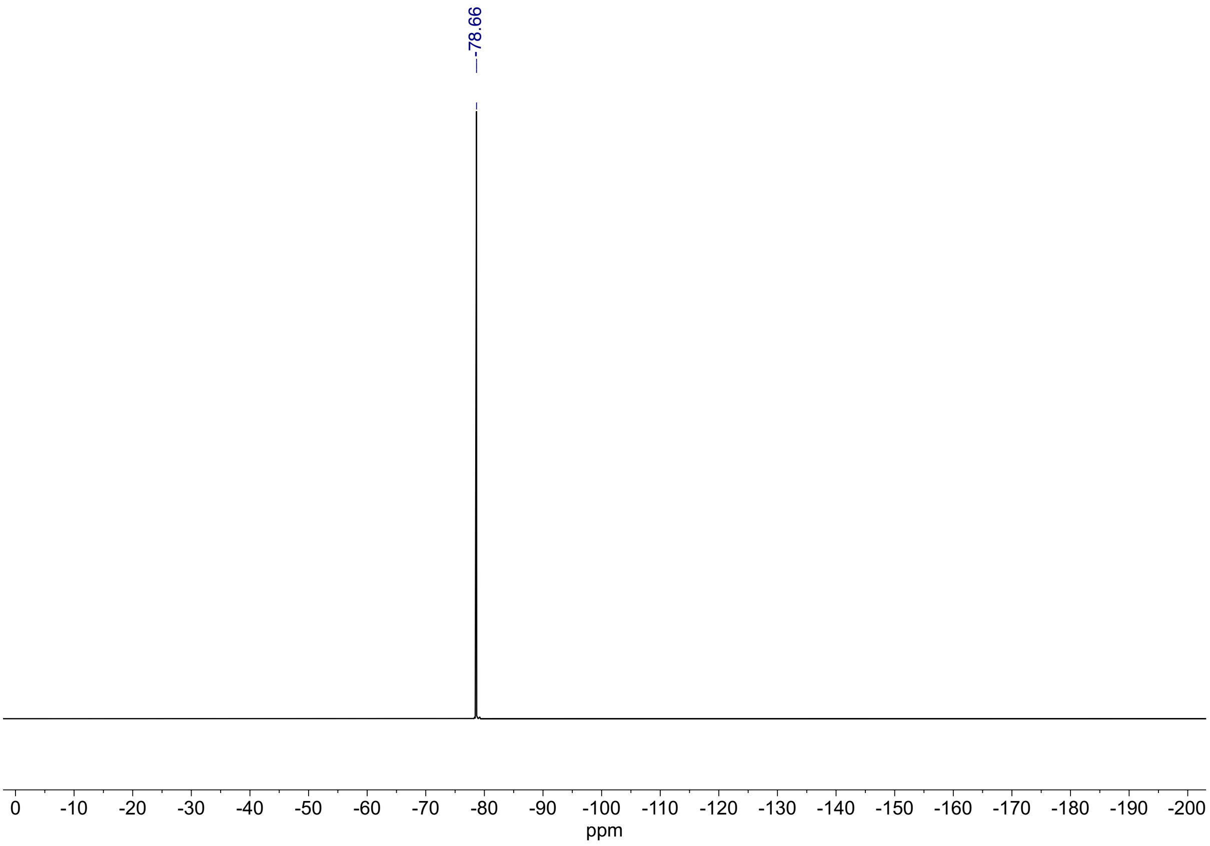

Figure S34. ^{19}F NMR (DMSO-d_6, 600 MHz, 298 K) of $Pd_6L_{12}(NTf_2)_{12}$.

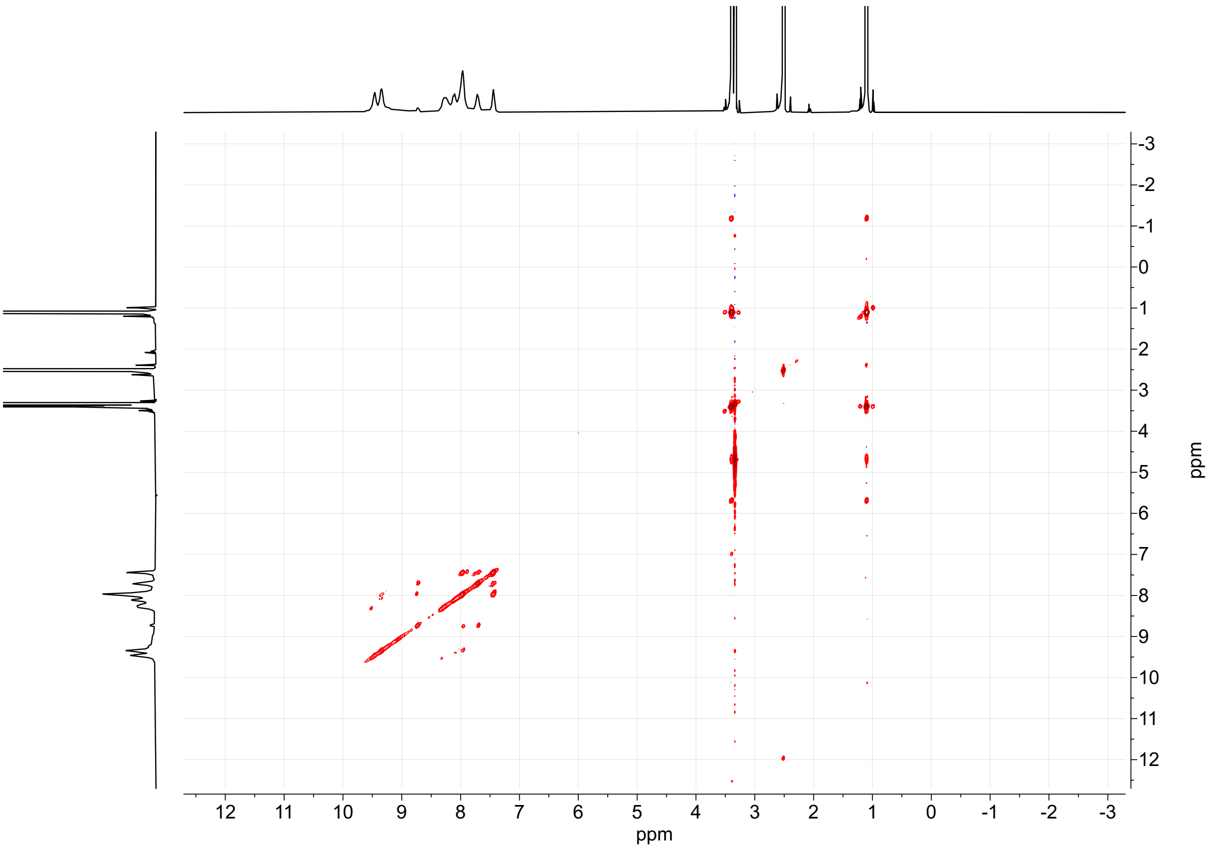

Figure S35. COSY NMR (DMSO-d_6, 600 MHz, 298 K) of $Pd_6L_{12}(NTf_2)_{12}$.

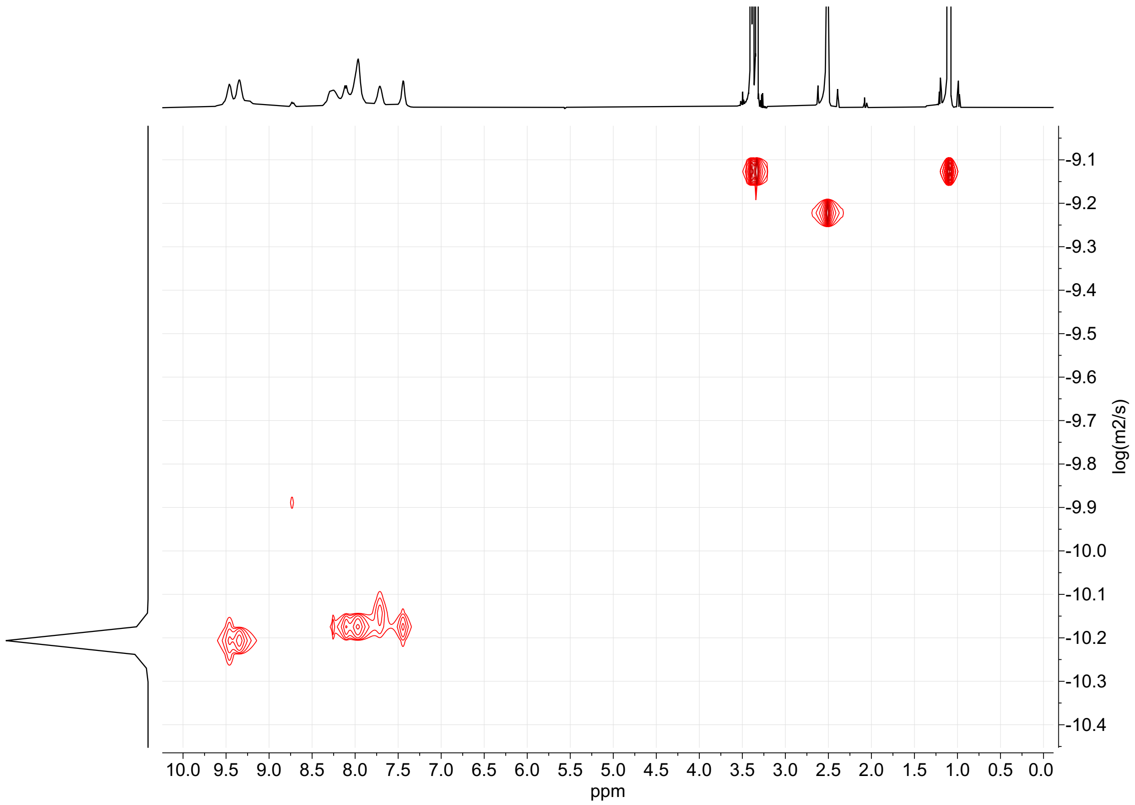

Figure S36. DOSY NMR (DMSO-*d*6, 600 MHz, 298 K) of $Pd_6L_{12}(NTf_2)_{12}$. The diffusion coefficient of $Pd_6L_{12}(NTf_2)_{12}$ was 6.7×10^{-11} m^2/s, giving a hydrodynamic radius of 11.5 Å.

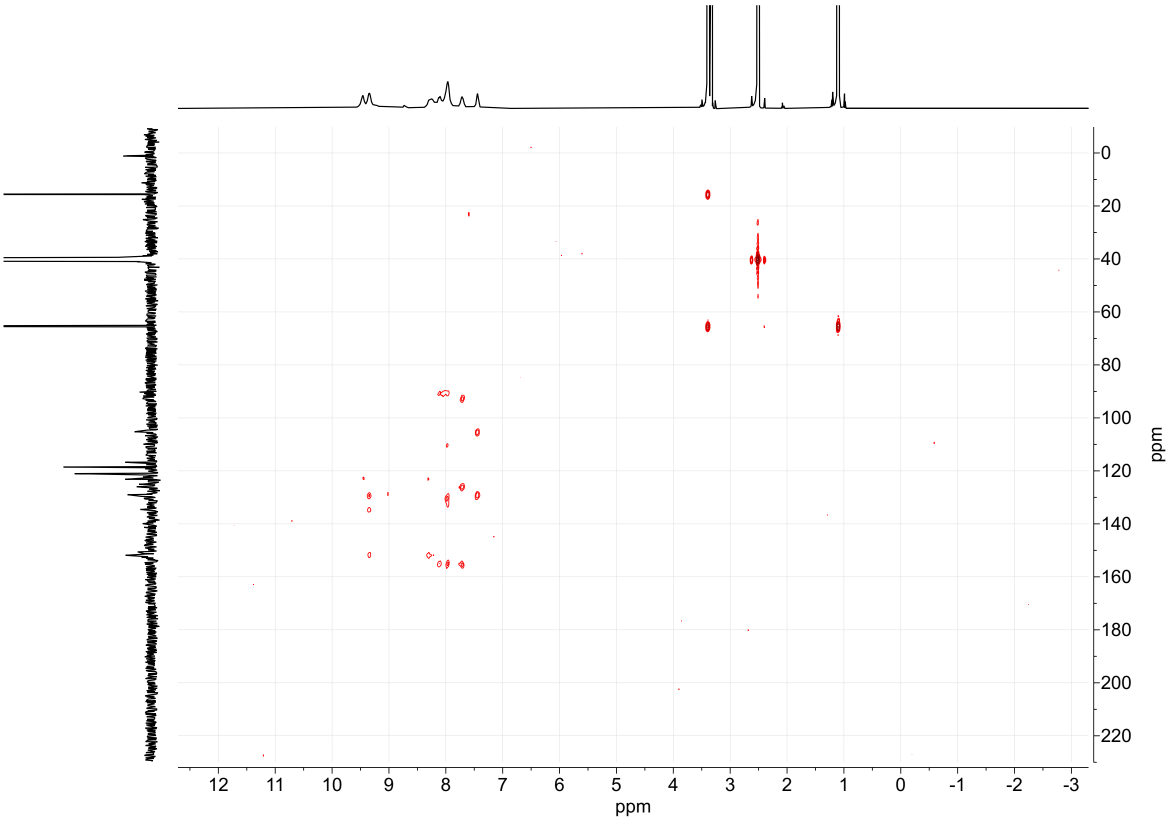

Figure S37. HMBC NMR (DMSO-*d*6, 600 MHz, 298 K) of $Pd_6L_{12}(NTf_2)_{12}$.

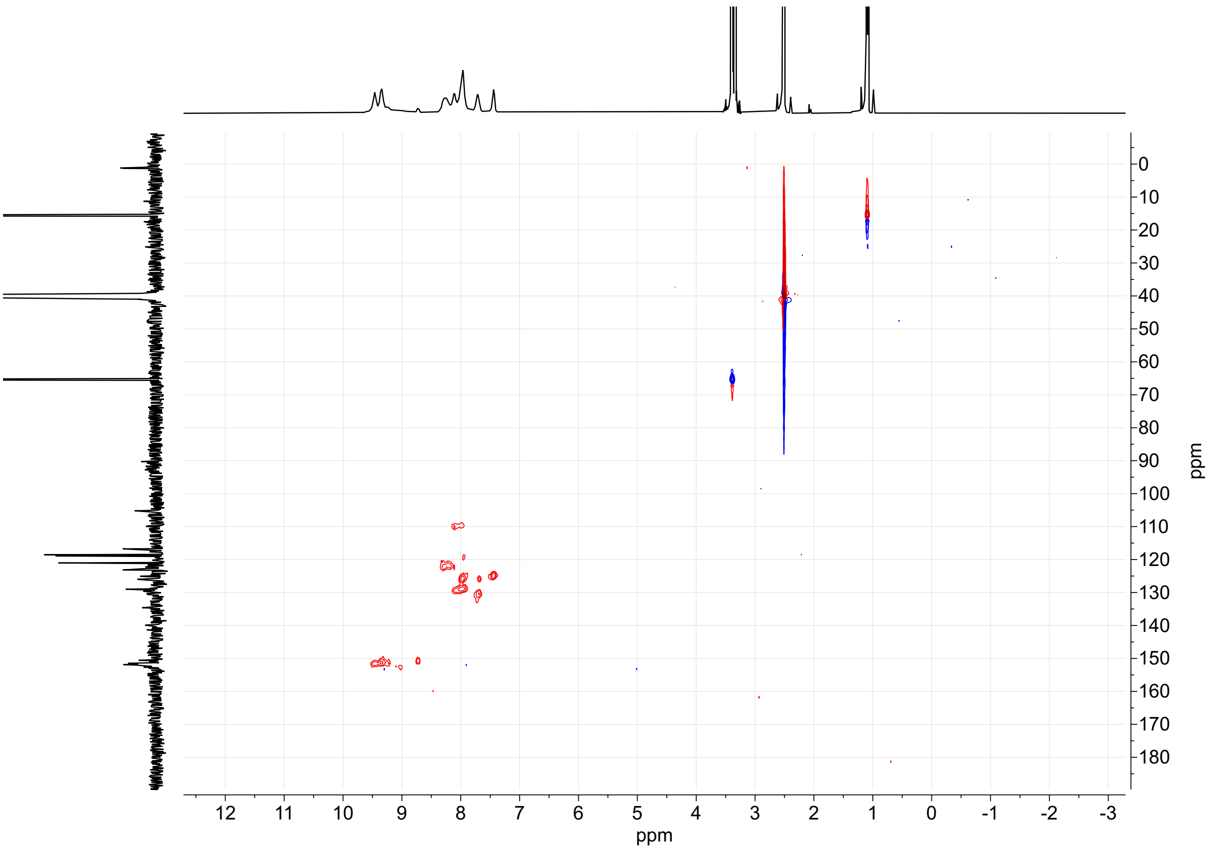

Figure S38. HSQC NMR (DMSO-*d*6, 600 MHz, 298 K) of $Pd_6L_{12}(NTf_2)_{12}$.

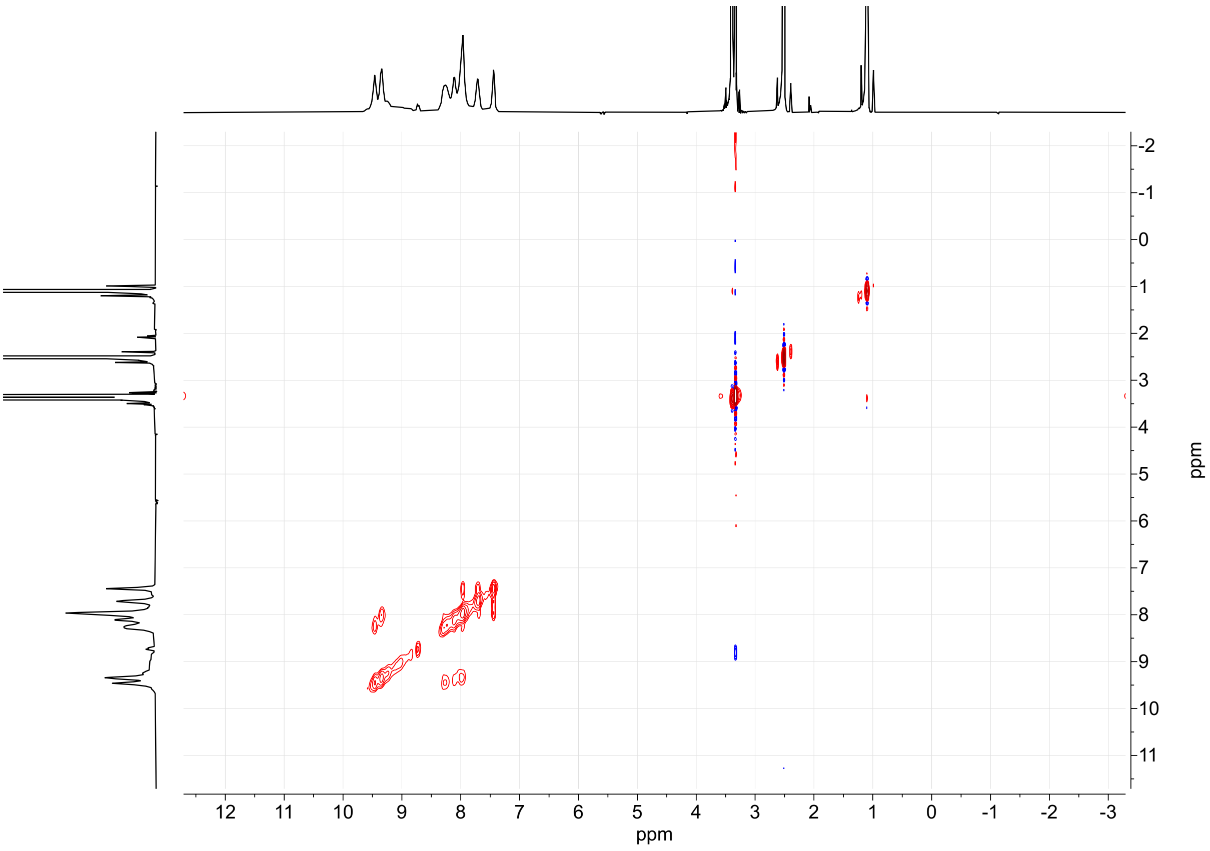

Figure S39. NOSEY NMR (DMSO-*d*6, 600 MHz, 298 K) of $Pd_6L_{12}(NTf_2)_{12}$.

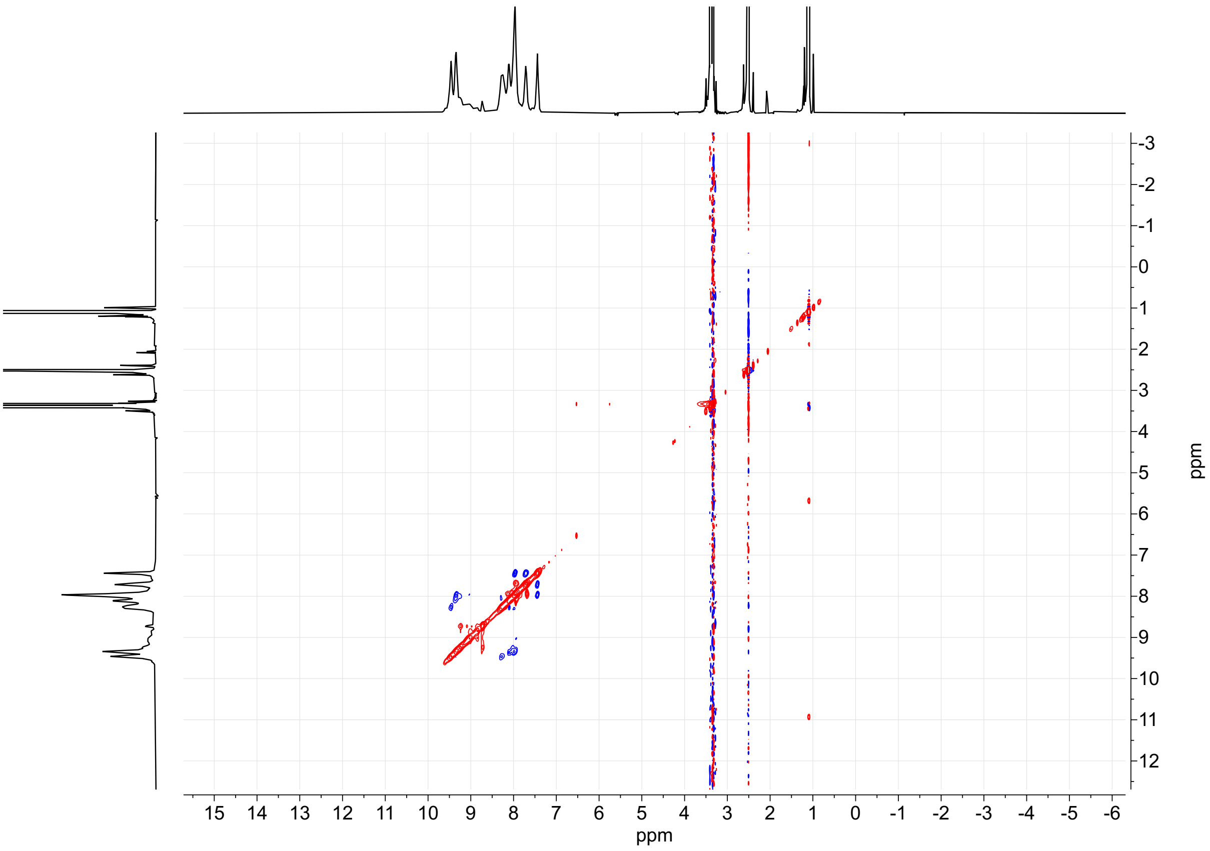

Figure S40. ROSEY NMR (DMSO-d_6, 600 MHz, 298 K) of $Pd_6L_{12}(NTf_2)_{12}$.

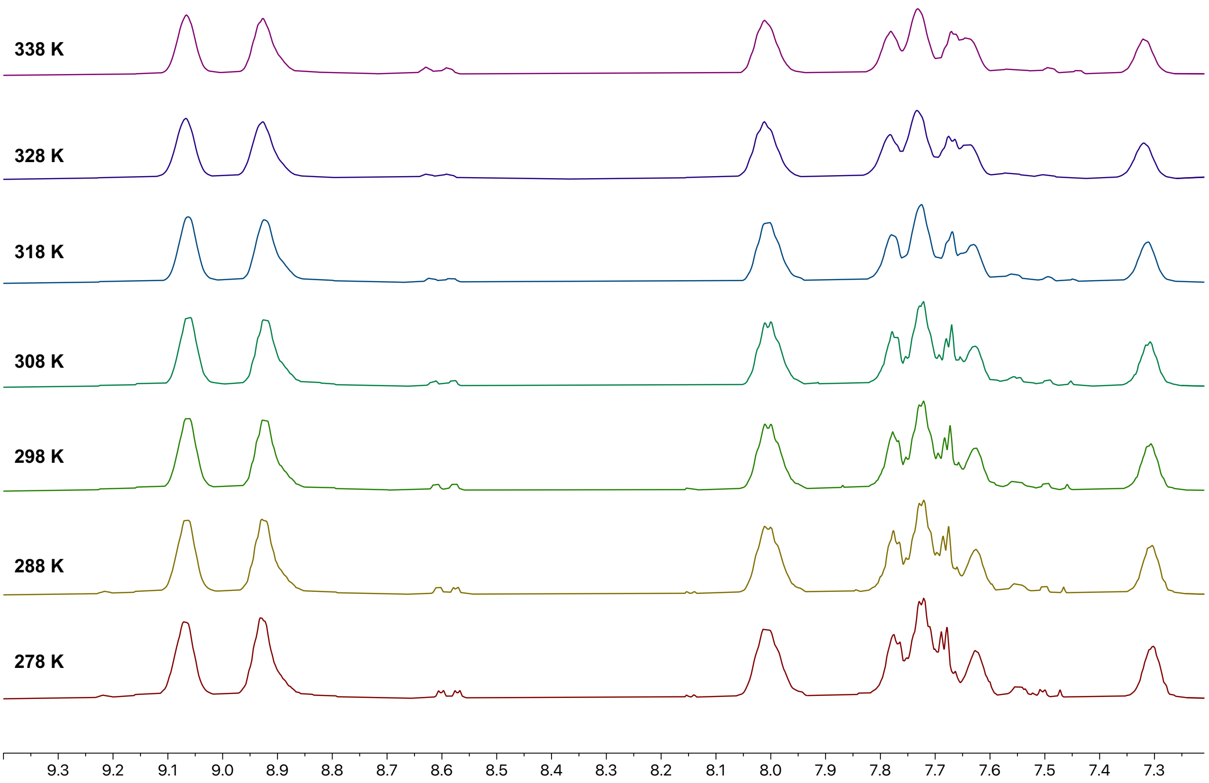

Figure S41. VT NMR(d_3-MeCN, 600 MHz, 298 K) of $Pd_6L_{12}(NTf_2)_{12}$ from 278K to 338K.

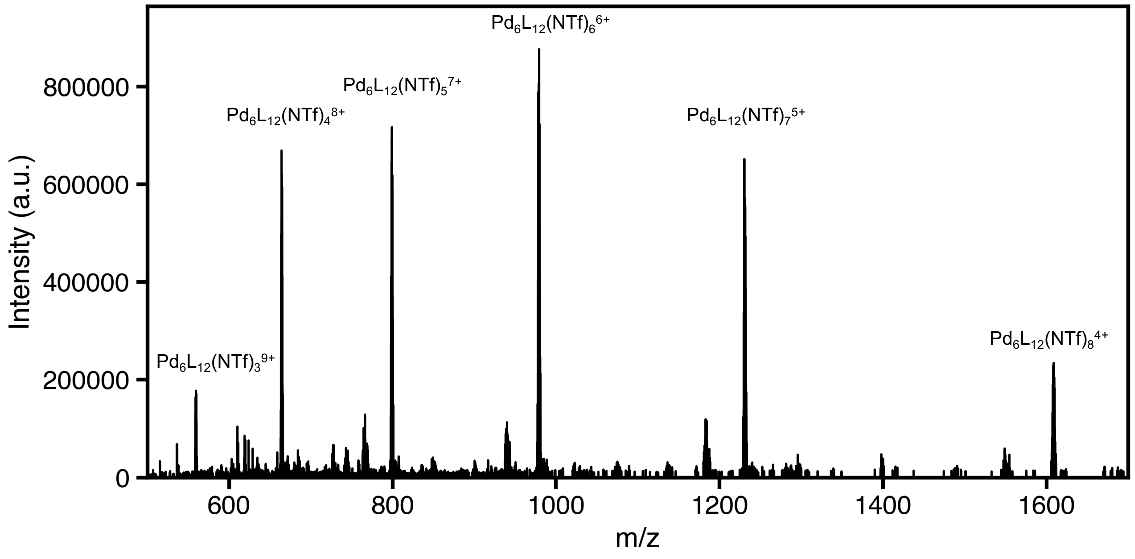

Figure S42. ESI-MS (positive-ion mode, CH_3CN) of $Pd_6L_{12}(NTf_2)_{12}$.

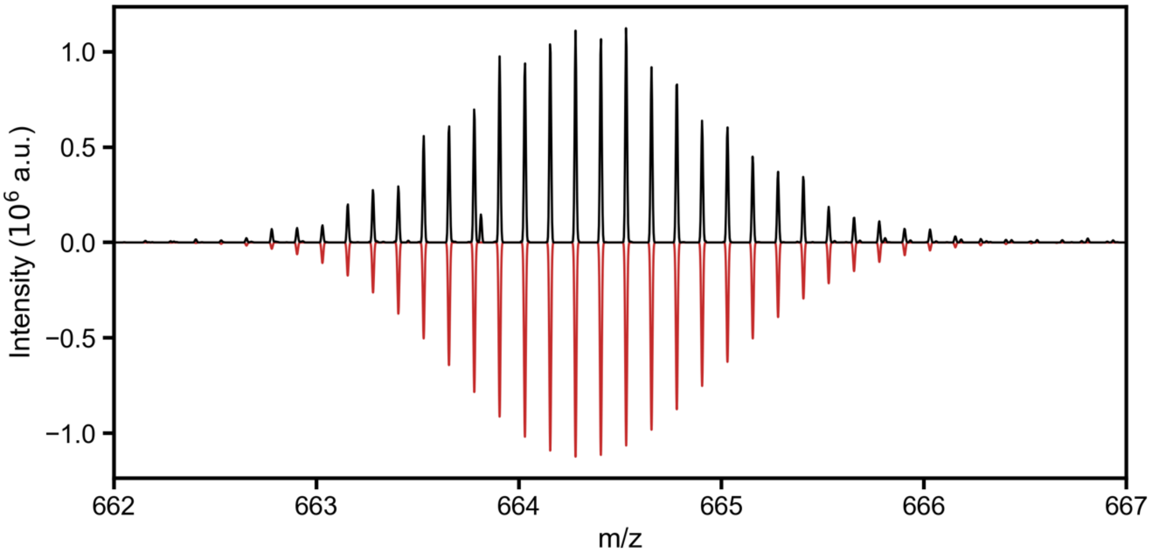

Figure S43. HRMS experimental (top) and calculated (bottom) isotopic distribution of $[Pd_6L_{12}(NTf_2)_4]^{8+}$.

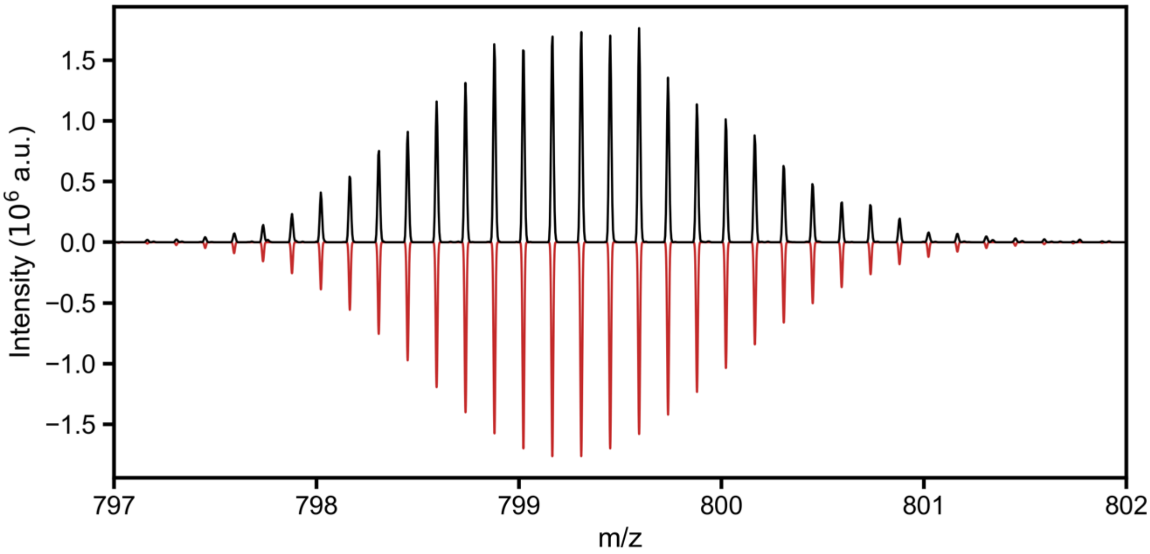

Figure S44. HRMS experimental (top) and calculated (bottom) isotopic distribution of $[Pd_6L_{12}(NTf_2)_5]^{7+}$.

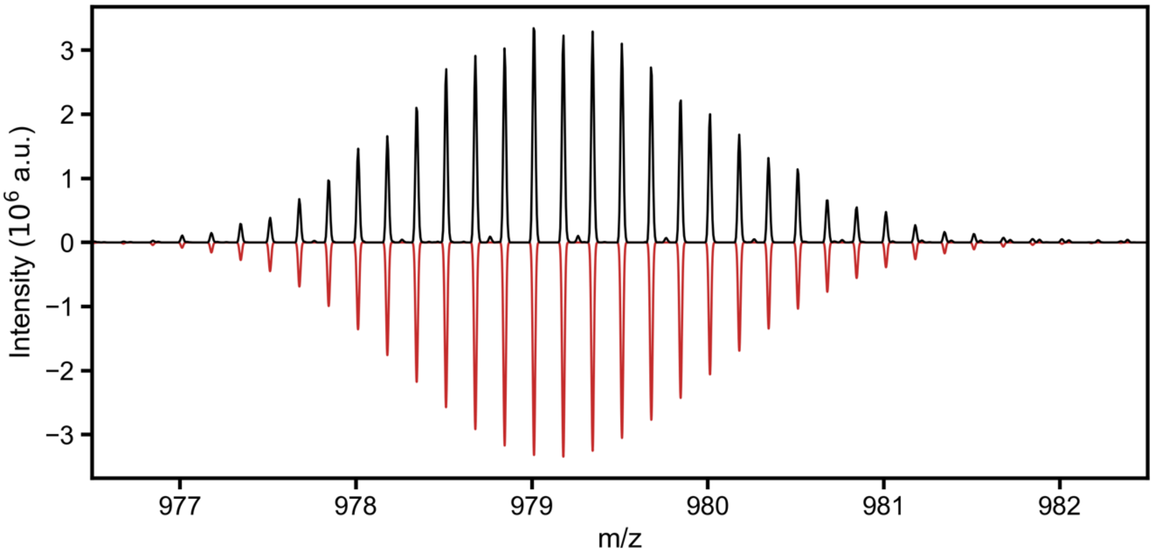

Figure S45. HRMS experimental (top) and calculated (bottom) isotopic distribution of $[Pd_6L_{12}(NTf_2)_6]^{6+}$.

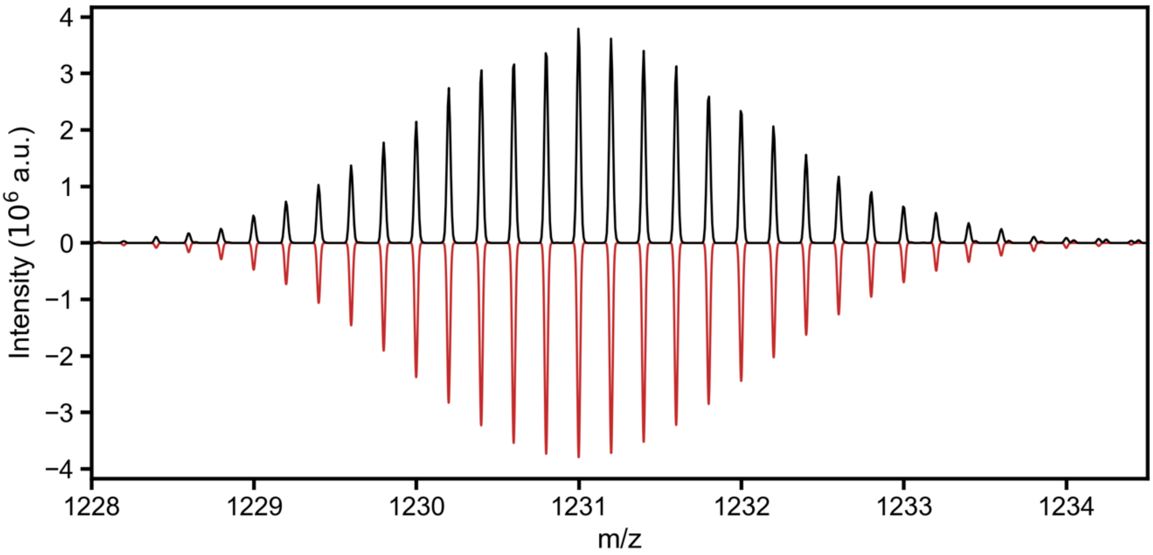

Figure S46. HRMS experimental (top) and calculated (bottom) isotopic distribution of $[Pd_6L_{12}(NTf_2)_7]^{5+}$.

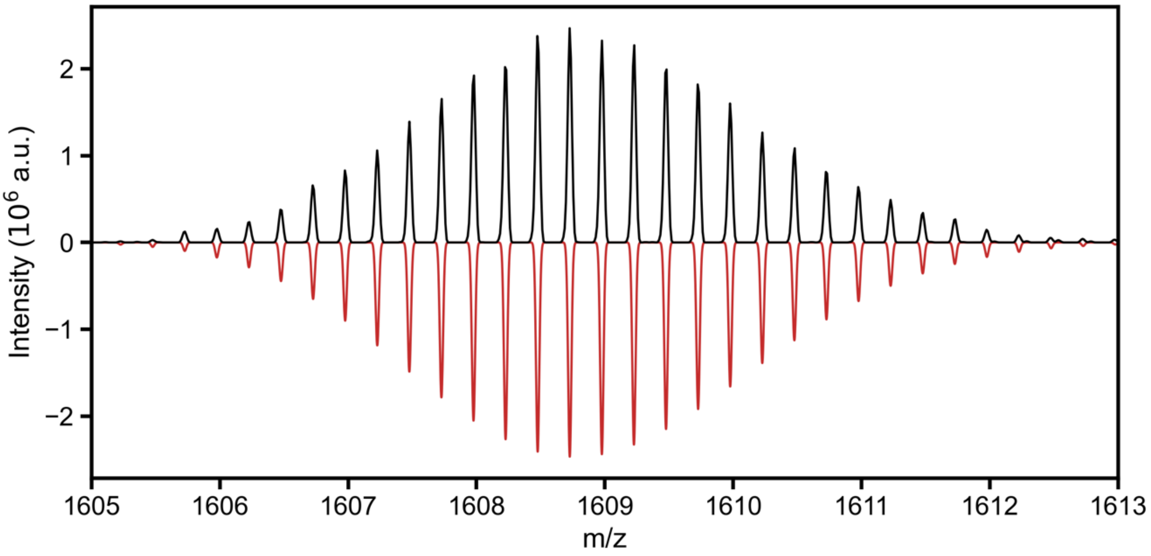

Figure S47. HRMS experimental (top) and calculated (bottom) isotopic distribution of $[Pd_6L_{12}(NTf_2)_8]^{4+}$.

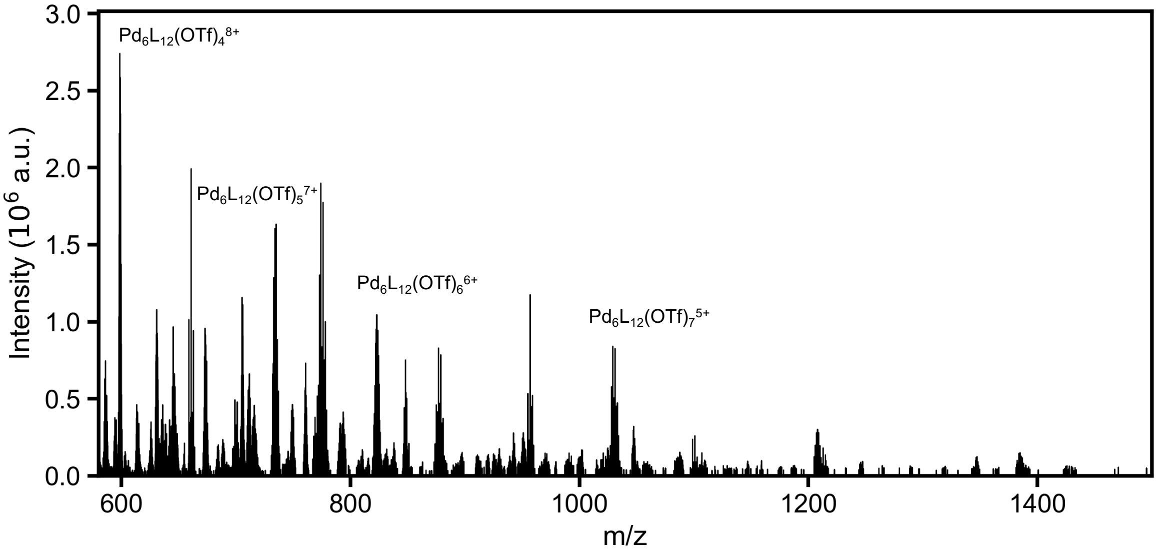

Figure S48. ESI-MS (positive-ion mode, CH_3CN) of $Pd_6L_{12}(OTf)_{12}$.

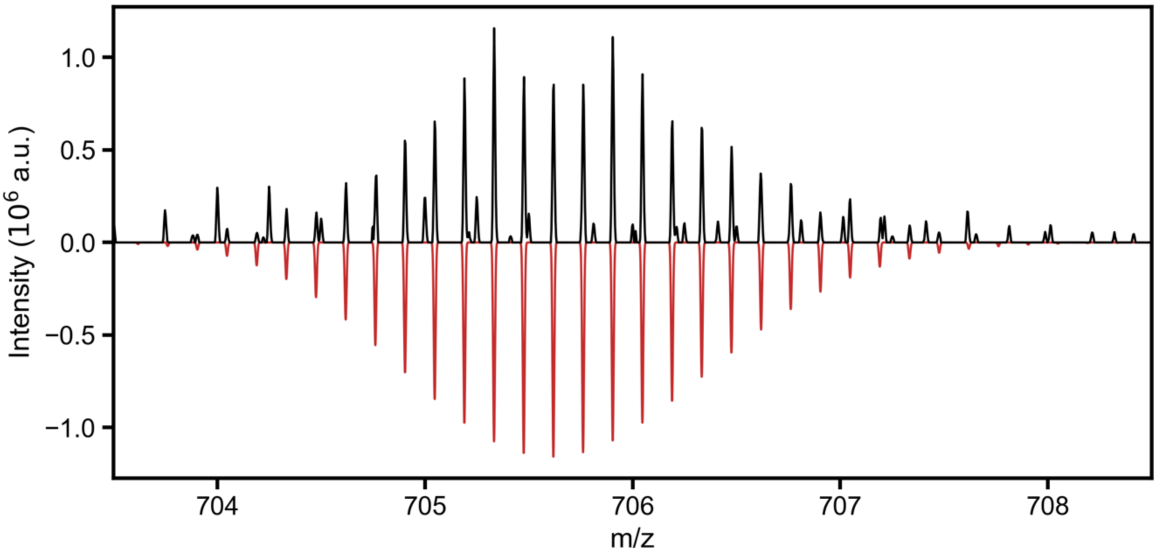

Figure S49. HRMS experimental (top) and calculated (bottom) isotopic distribution of $[Pd_6L_{12}(OTf)_5]^{7+}$.

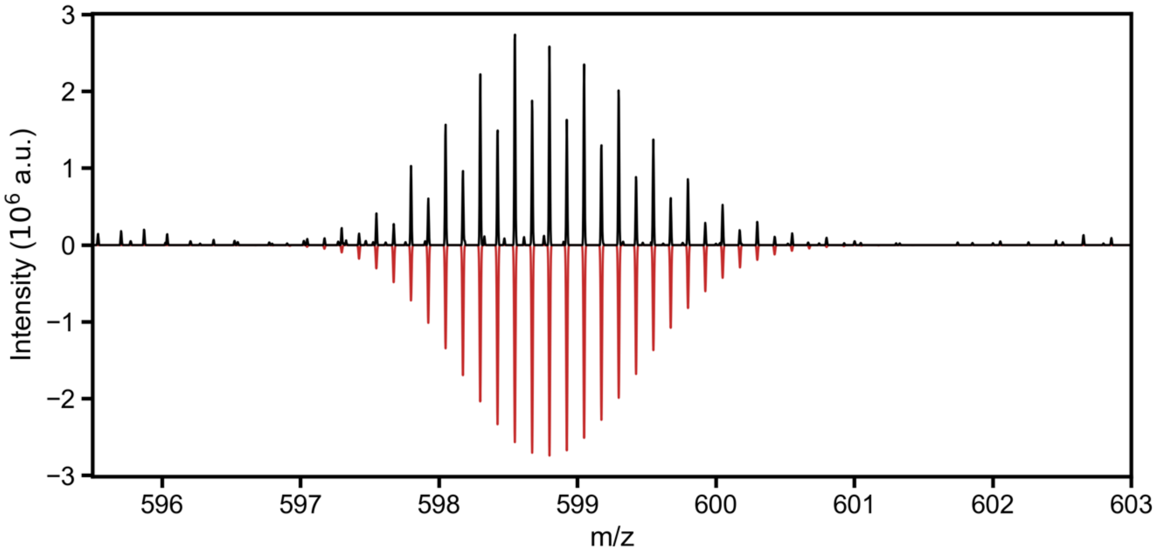

Figure S50. HRMS experimental (top) and calculated (bottom) isotopic distribution of $[Pd_6L_{12}(OTf)_4]^{8+}$.

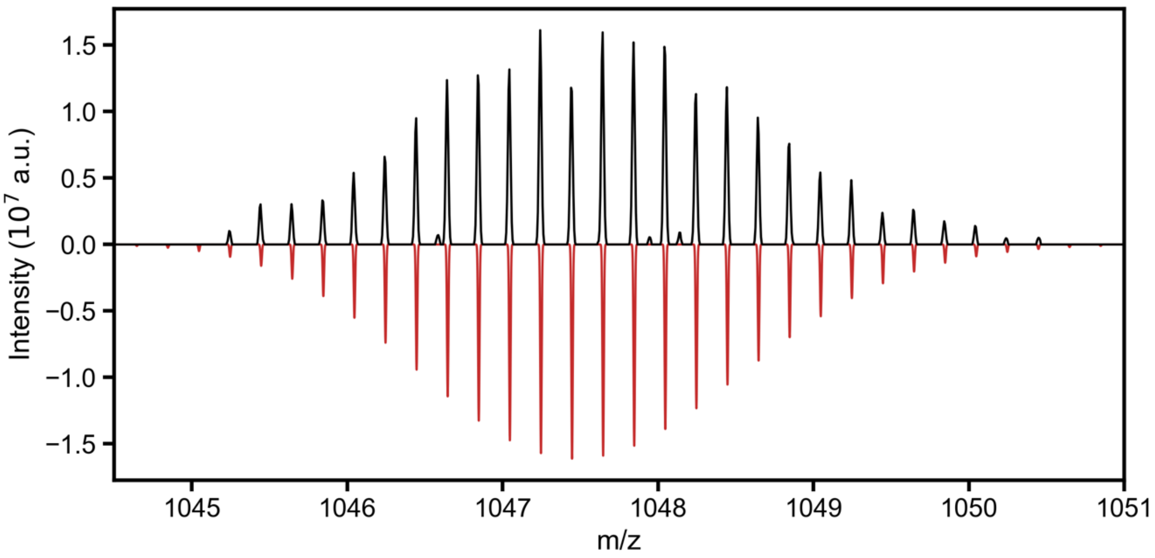

Figure S51. HRMS experimental (top) and calculated (bottom) isotopic distribution of $[Pd_6L_{12}(OTf)_7]^{5+}$.

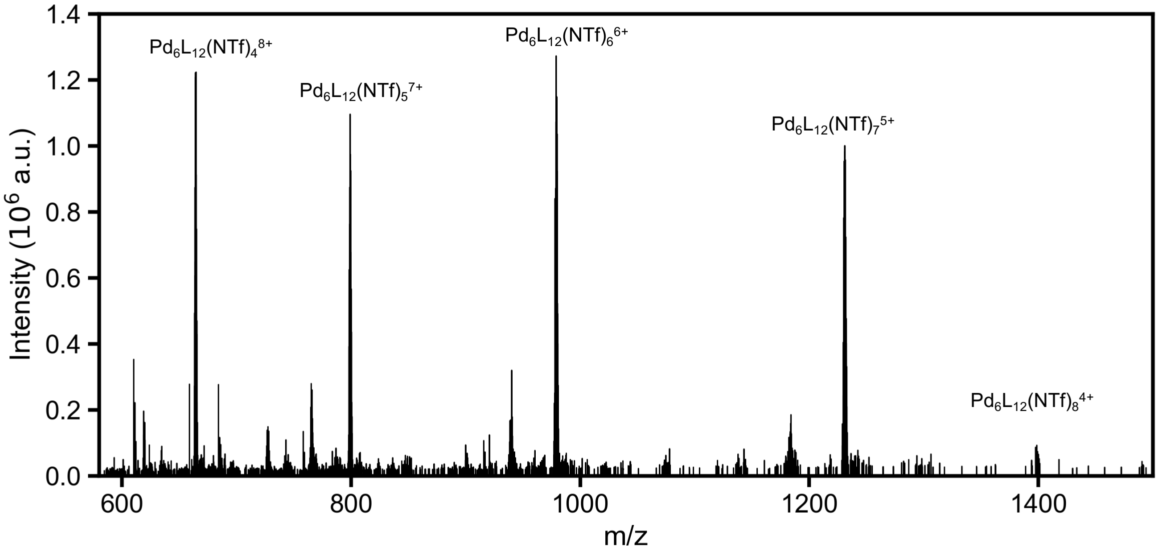

Figure S52. ESI-MS (positive-ion mode, CH_3CN) of $Pd_6L_{12}(OTf)_{12}$ equilibrated (16 hr) in DMSO, indicates that Pd_6L_{12} cage remains stable at room temperature in DMSO.

3. NMR Host-Guest Association Studies

A 0.3 mM stock solution of $Pd_4L_8(BF_4)_8$ or $Pd_6L_{12}(NTf_2)_{12}$ was prepared in d_3-MeCN. The residual proton signal of MeCN (δ = 1.96 ppm) was used as an internal reference for chemical shift calibration. 30 mM stock solutions of the guest molecules were also prepared in d_3-MeCN. For each experiment, 0.4 mL of the cage solution was transferred to an NMR tube, followed by the addition of 1 equivalent of the guest.

The resulting mixtures were analysed by ^{1}H NMR spectroscopy. Evidence of host-guest association was determined by: (i) chemical shift changes in cage and/or guest resonances; and/or (ii) the appearance of a cage–guest adduct signal in the HRMS spectrum.

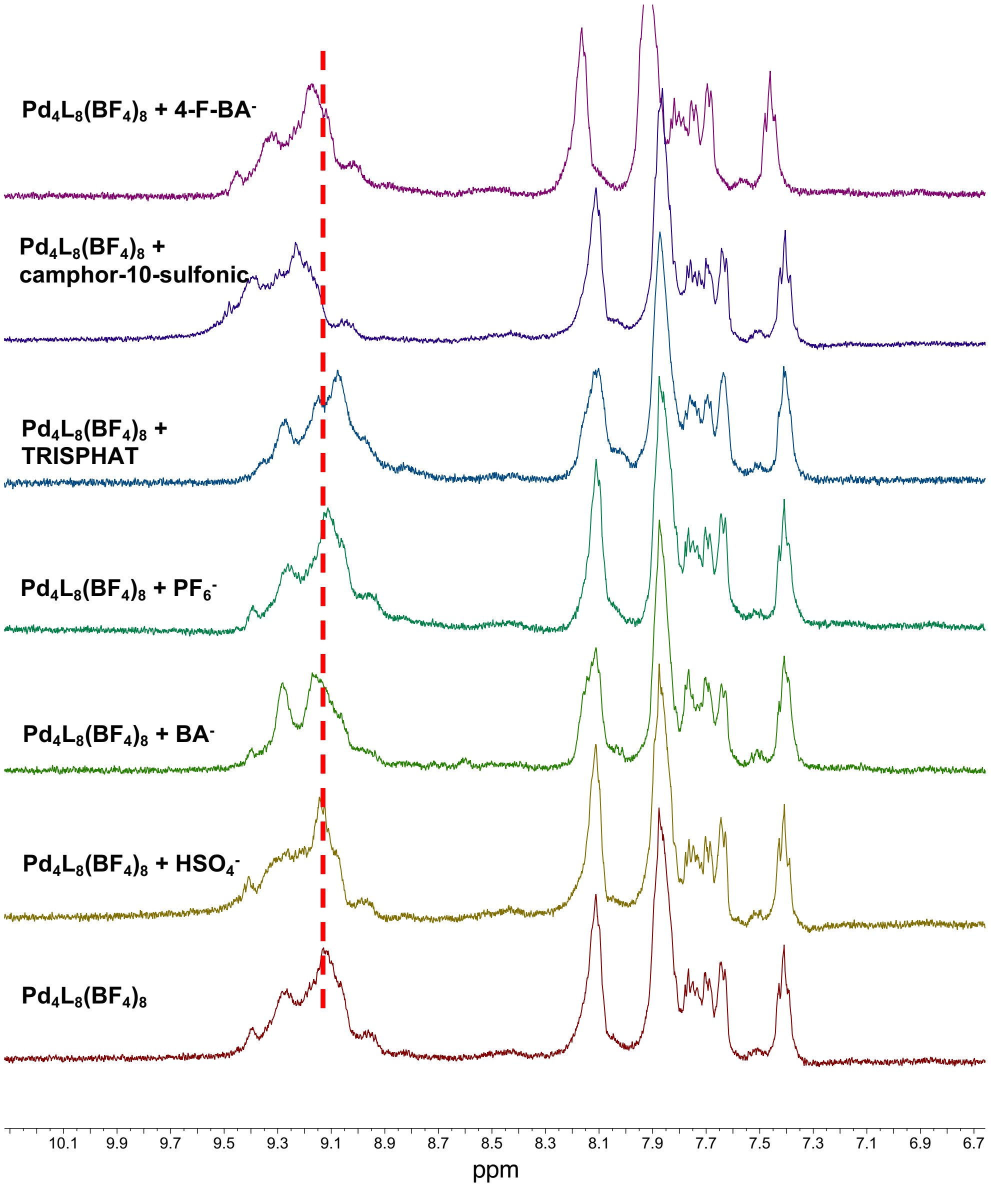

Figure S53. Stacked ^{1}H NMR for Cage $Pd_4L_8(BF_4)_8$ with different guests (d_3-MeCN, 400 MHz, 298 K). Dashed line representing exemplar peak shift.

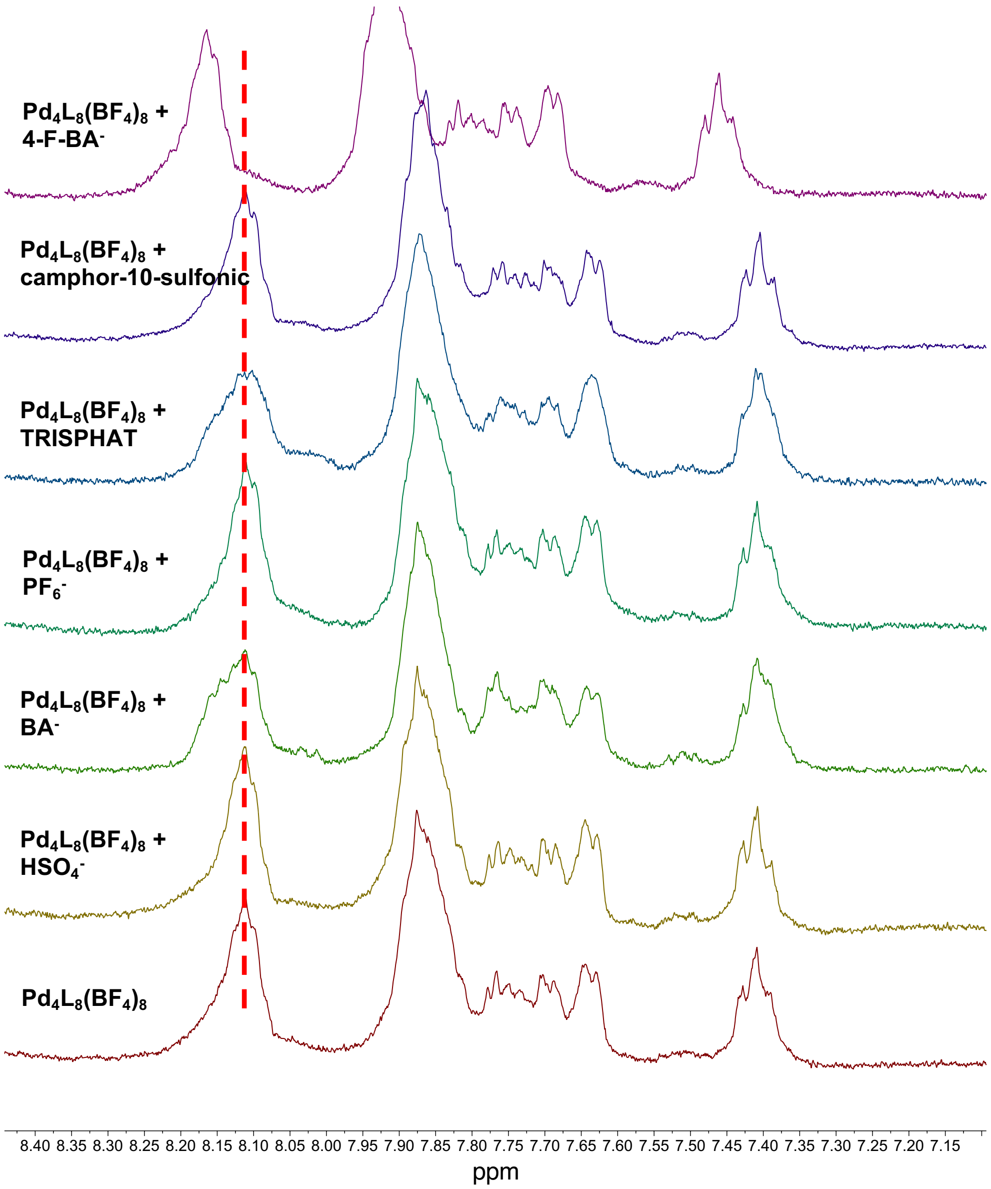

Figure S54. Stacked ^{1}H NMR for Cage $Pd_4L_8(BF_4)_8$ with different guests (d_3-MeCN, 400 MHz, 298 K).

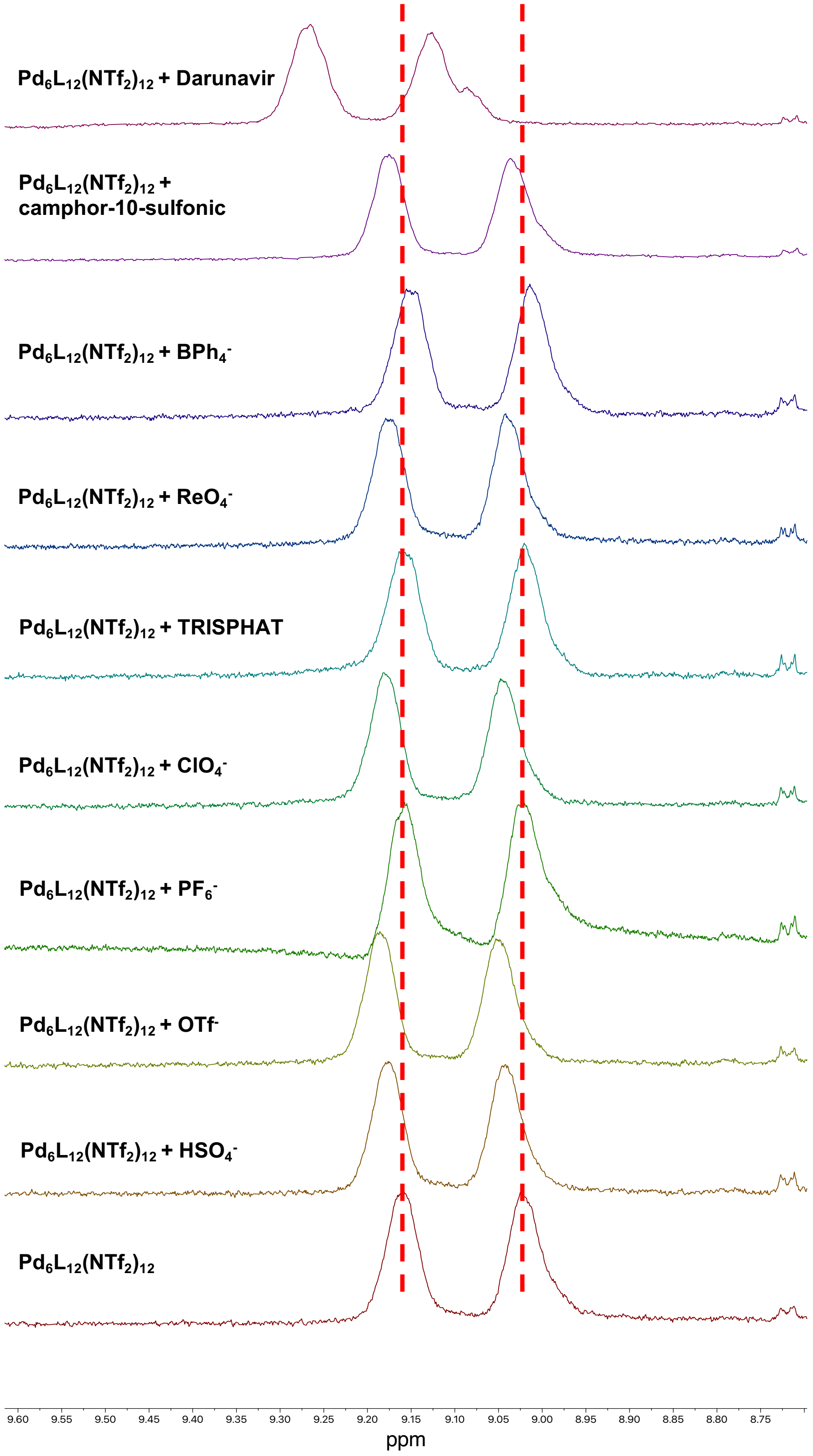

Figure S55. Stacked ^1H NMR for the $Pd_6L_8(NTf_2)_{12}$ with different guests (d_3-MeCN, 400 MHz, 298 K) and exemplar shifts highlighted.

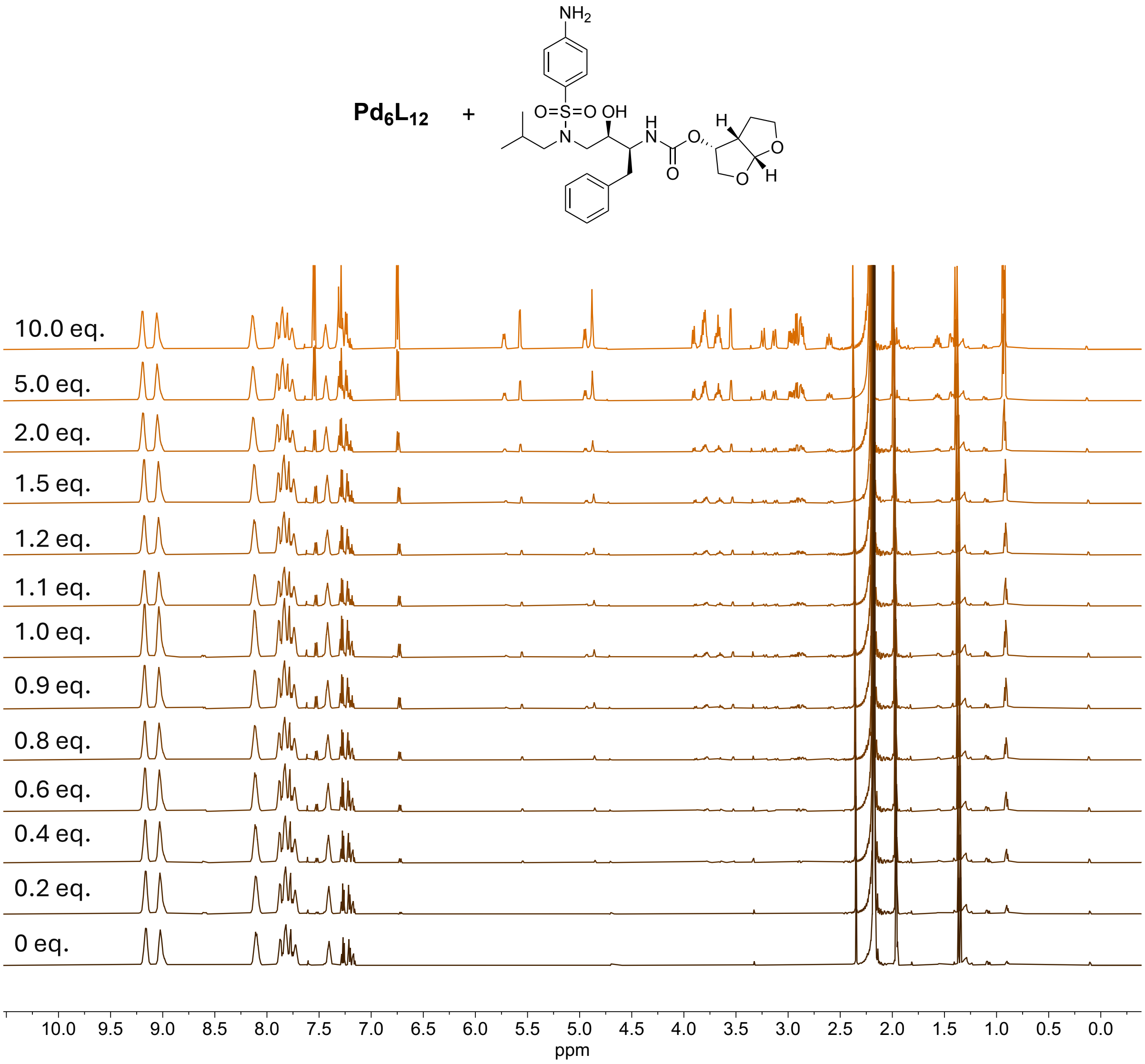

Figure S56. ^{1}H NMR titration (d_3-MeCN, 600 MHz, 298 K) of $Pd_6L_{12}(NTf_2)_{12}$ (0.30 mM) and varying equivalents of Darunavir.

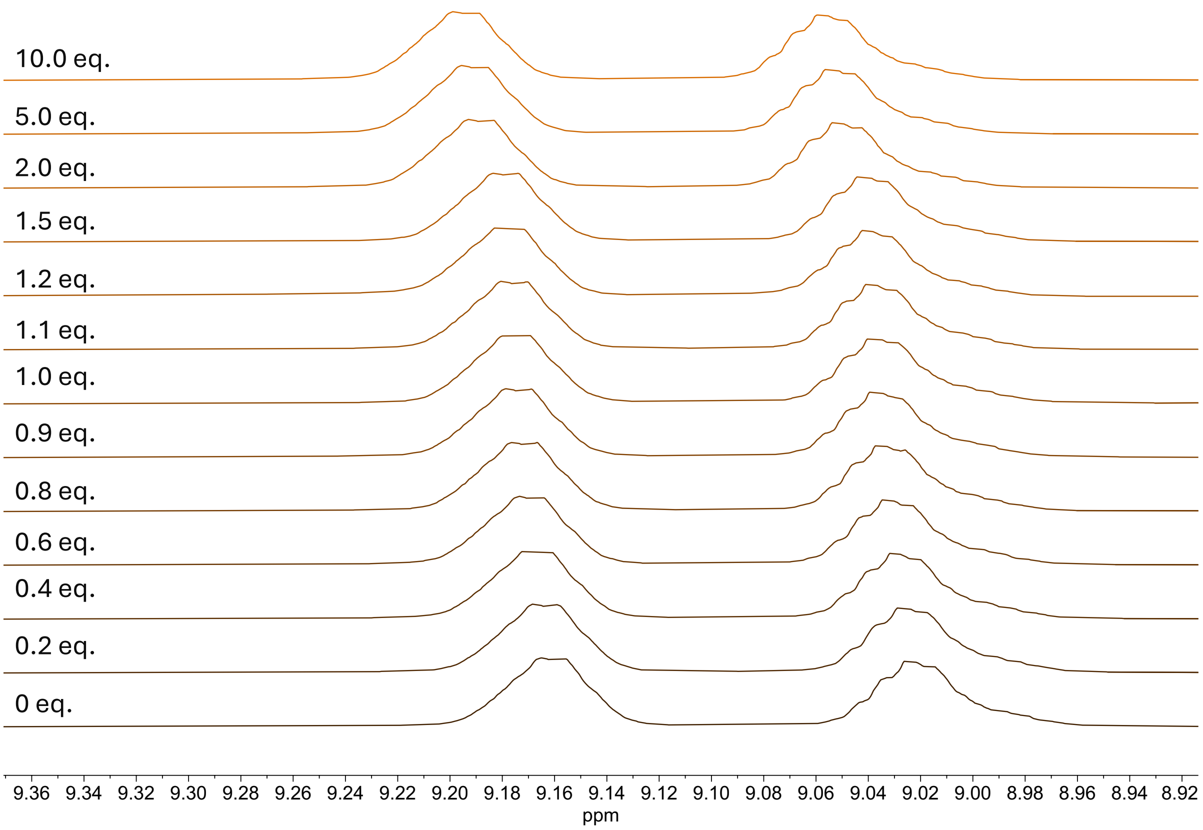

Figure S57. Partial ^{1}H NMR titration (d_3-MeCN, 600 MHz, 298 K) of $Pd_6L_{12}(NTf_2)_{12}$ (0.30 mM) and varying equivalents of Darunavir.

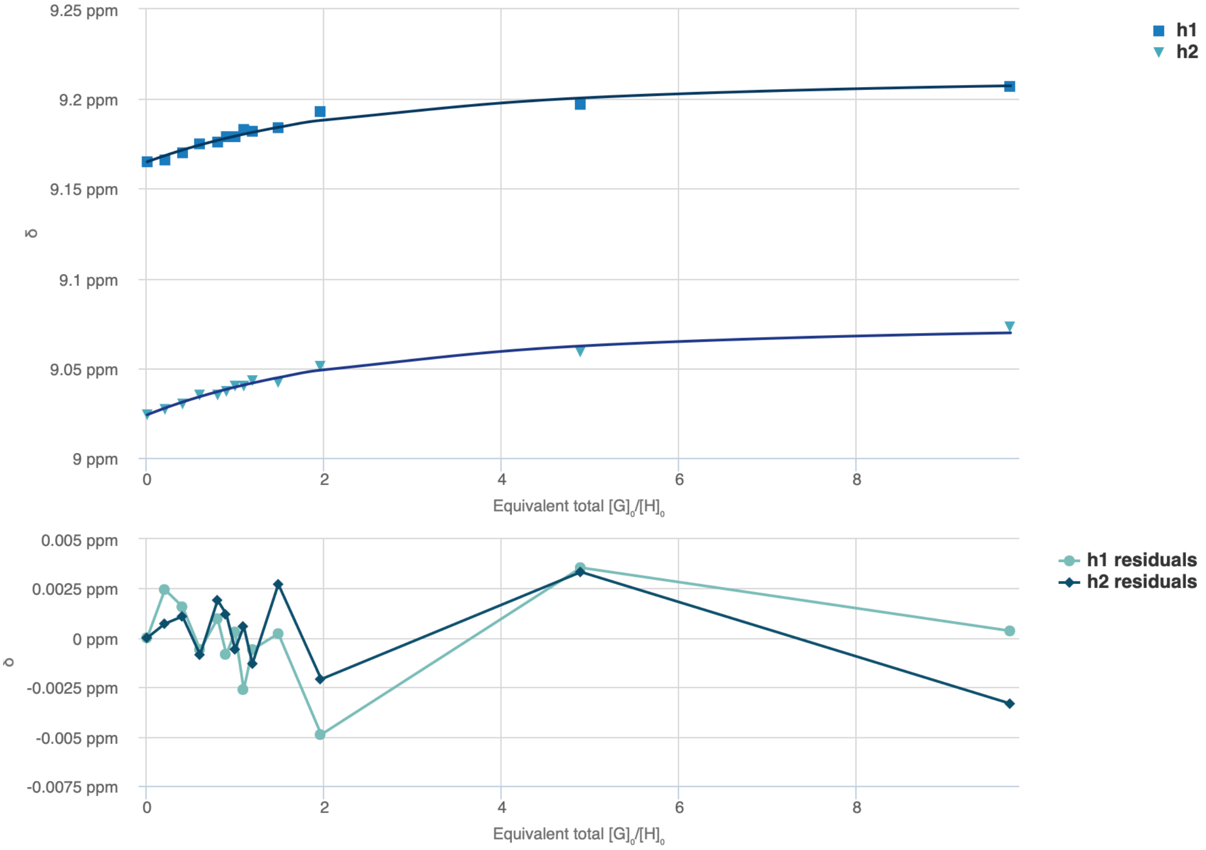

Figure S58. Binding data for $Pd_6L_{12}(NTf_2)_{12}$ and Darunavir, K_a = 1827.36 M^{-1} ± 10.9% using a 1:1model in Bindfit.

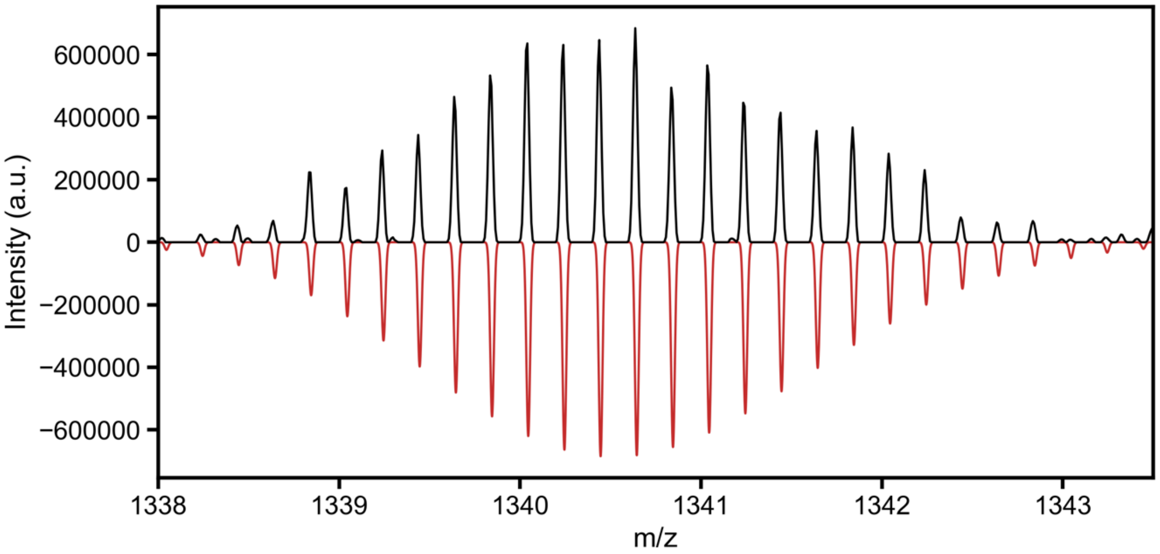

Figure S59. HRMS experimental (top) and calculated (bottom) isotopic distribution of the [**5**(NTf_2)$_7$] $^{5+}$ bound to one Darunavir as a guest.

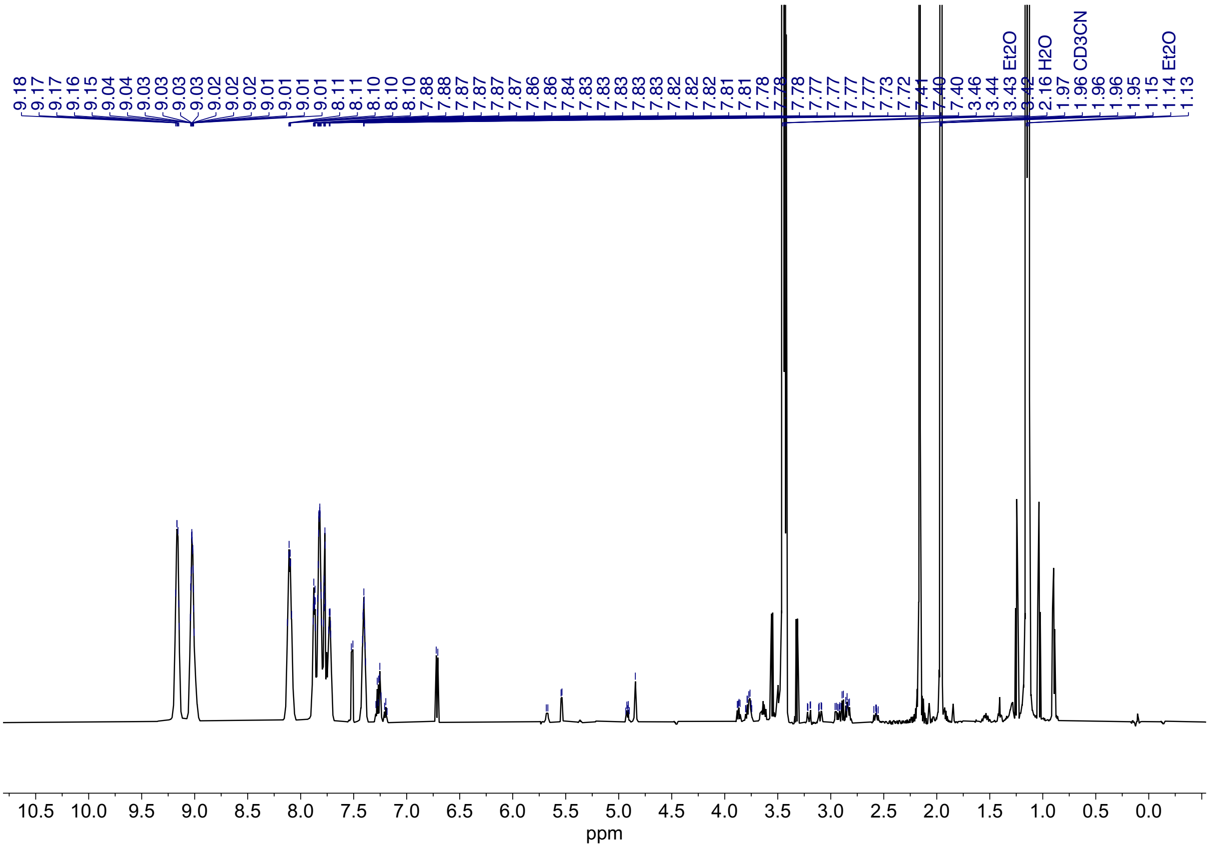

Figure S60. ^{1}H NMR (d_3-MeCN, 600 MHz, 298 K) of $Pd_6L_{12}(NTf_2)_{12}$.

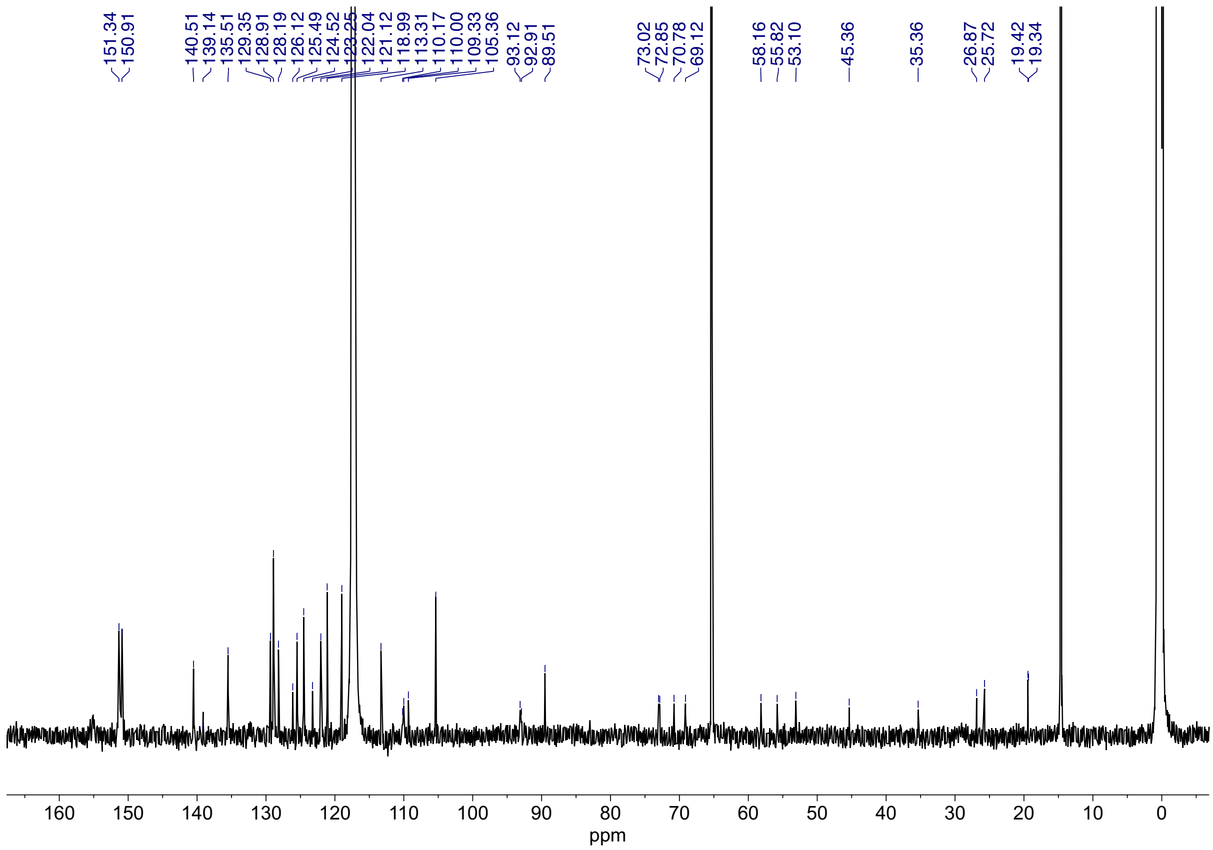

Figure S61. ^{13}C NMR (d_3-MeCN, 600 MHz, 298 K) of $Pd_6L_{12}(NTf_2)_{12}$ + Darunavir complex.

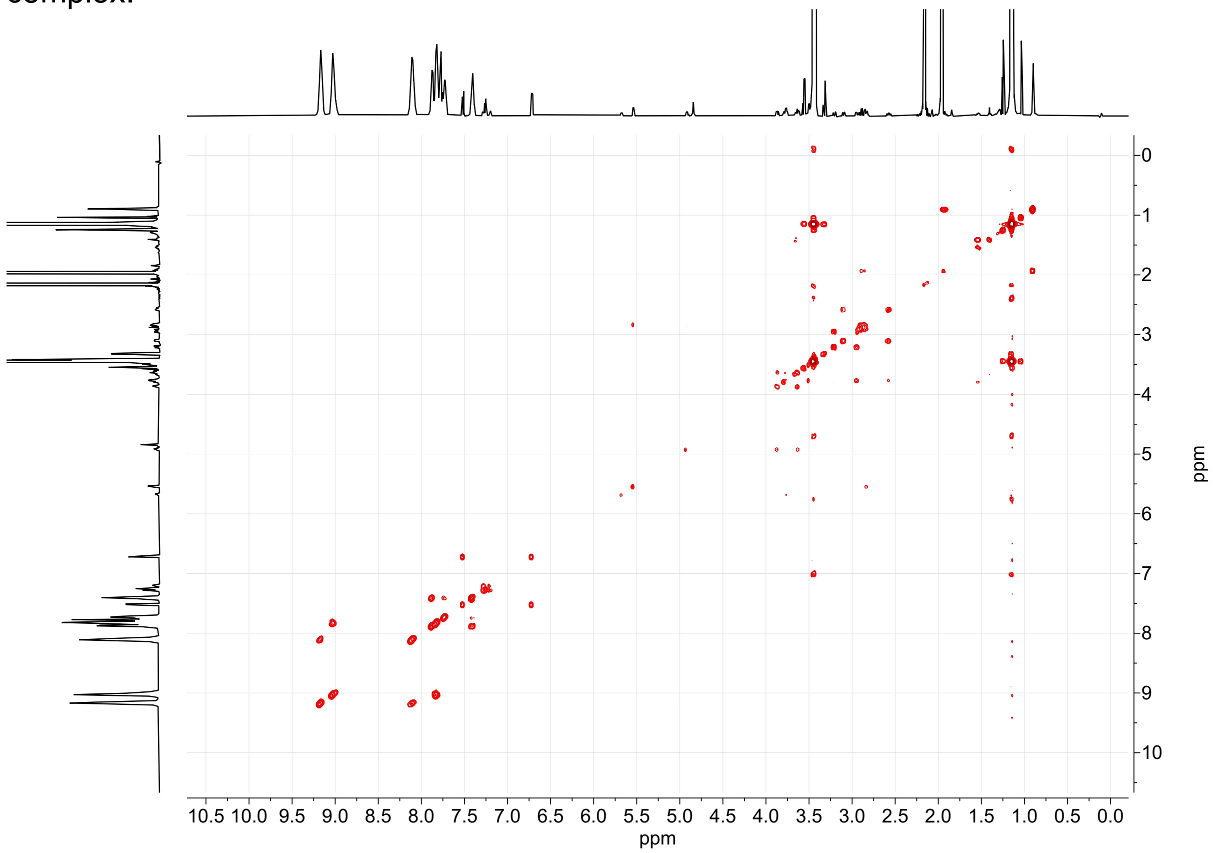

Figure S62. COSY NMR (d_3-MeCN, 600 MHz, 298 K) of $Pd_6L_{12}(NTf_2)_{12}$ + Darunavir complex.

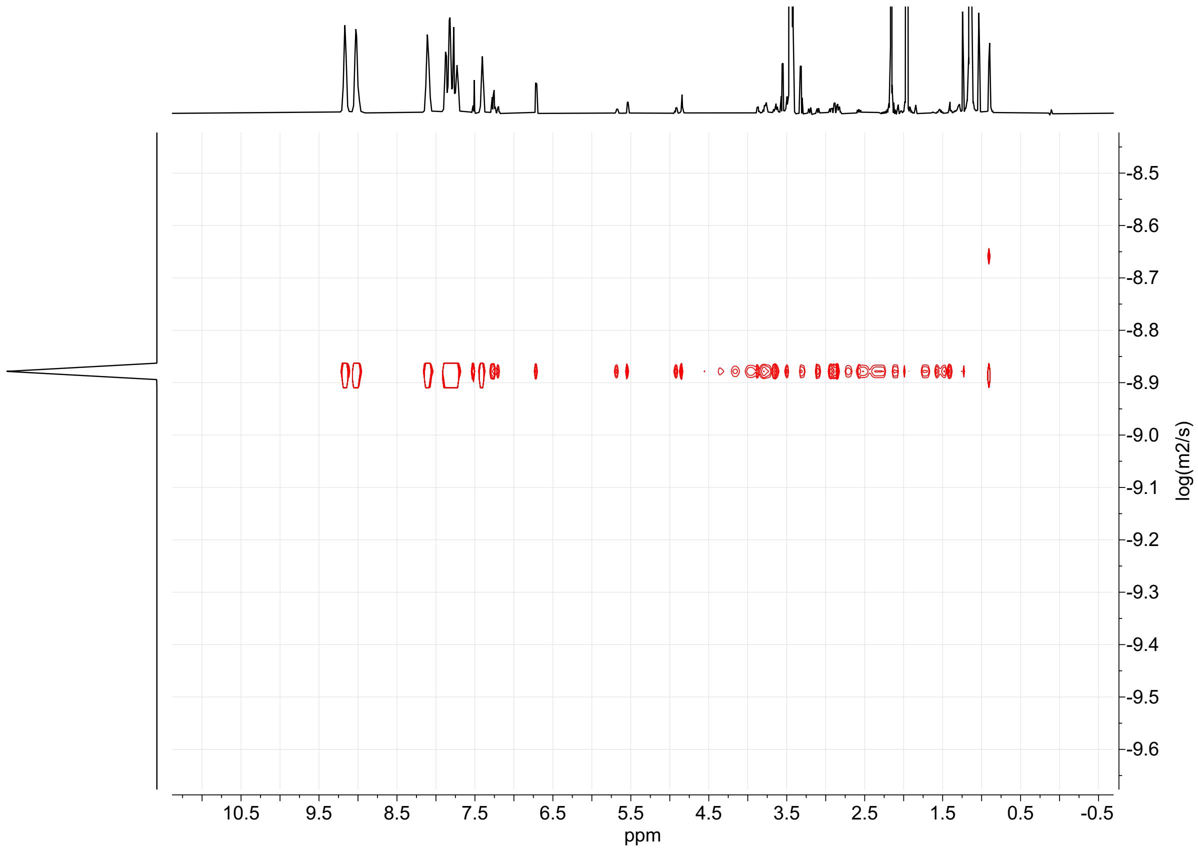

Figure S63. DOSY NMR (d_3-MeCN, 600 MHz, 298 K) of $Pd_6L_{12}(NTf_2)_{12}$ + Darunavir complex. A single diffusion band was observed, supporting complexation.

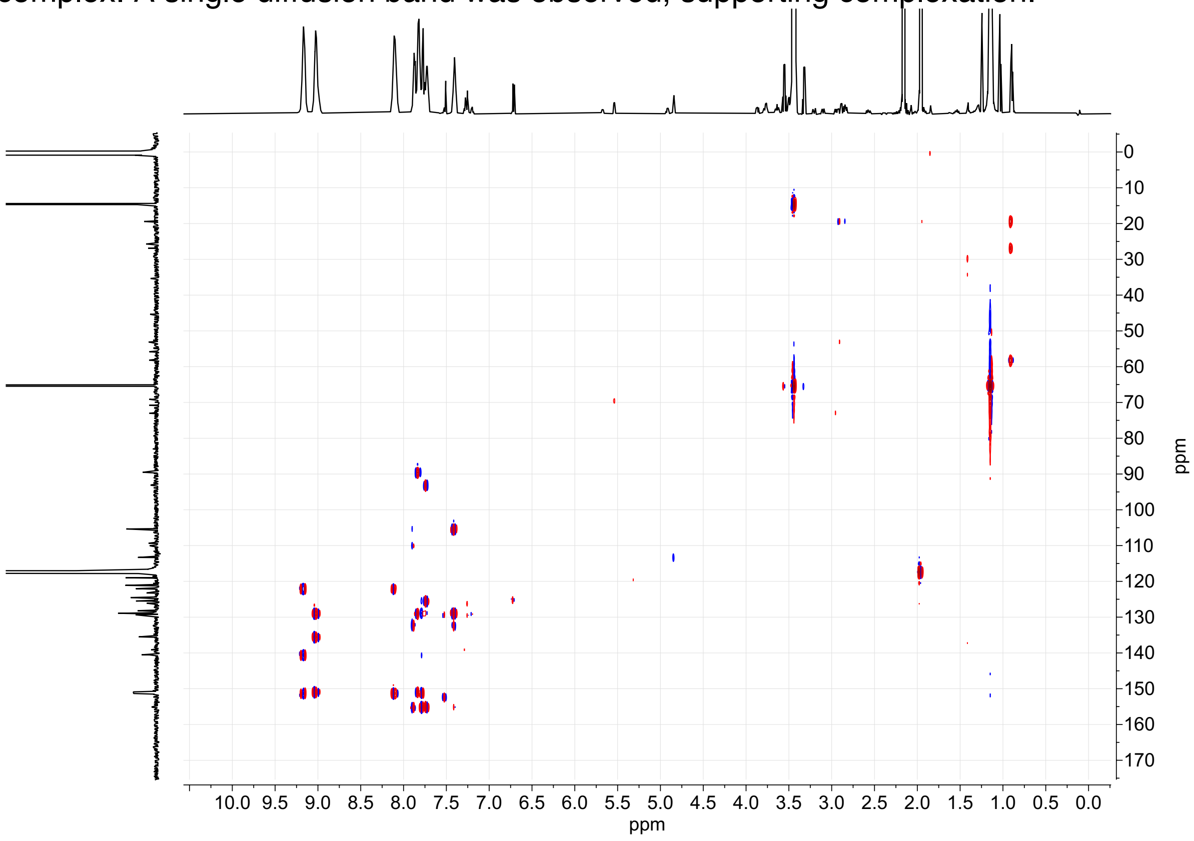

Figure S64. HMBC NMR (d_3-MeCN, 600 MHz, 298 K) of $Pd_6L_{12}(NTf_2)_{12}$ + Darunavir complex.

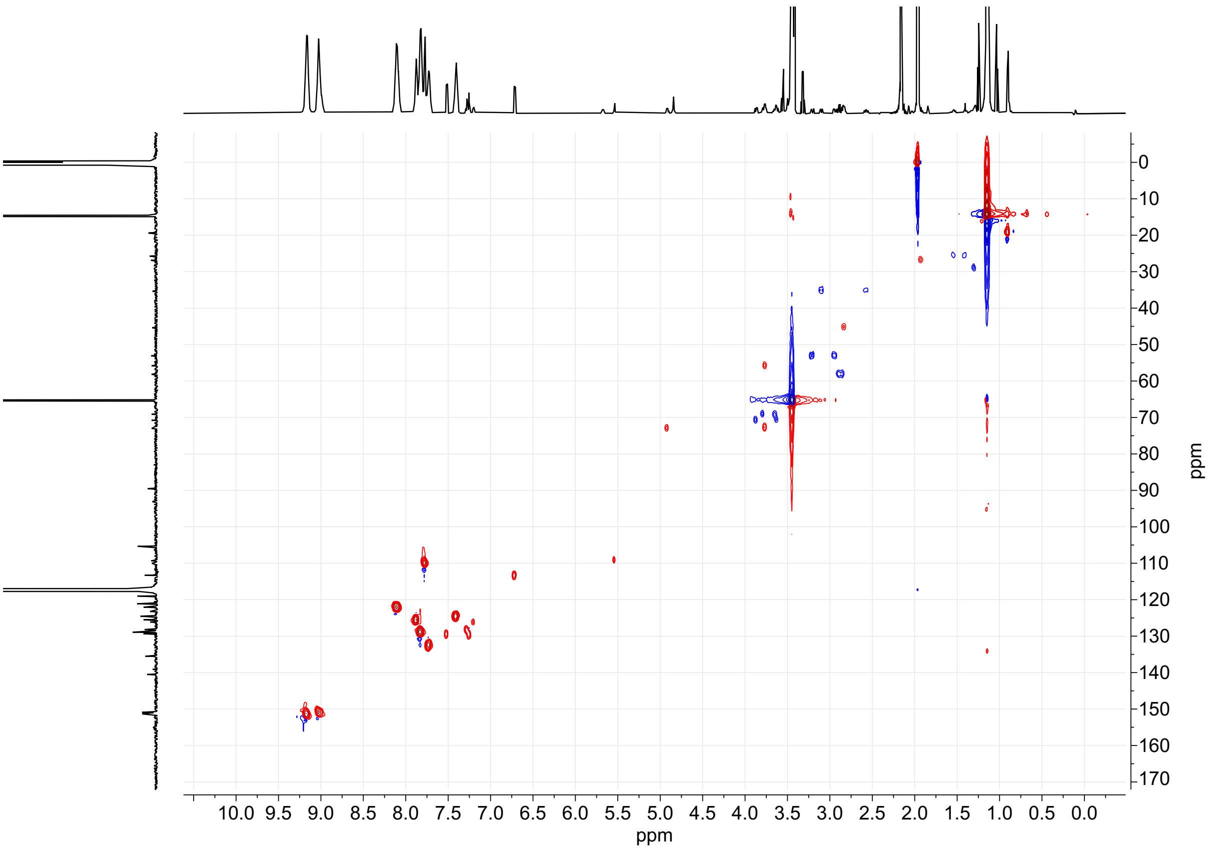

Figure S65. HSQC NMR (d_3-MeCN, 600 MHz, 298 K) of $Pd_6L_{12}(NTf_2)_{12}$ + Darunavir complex.

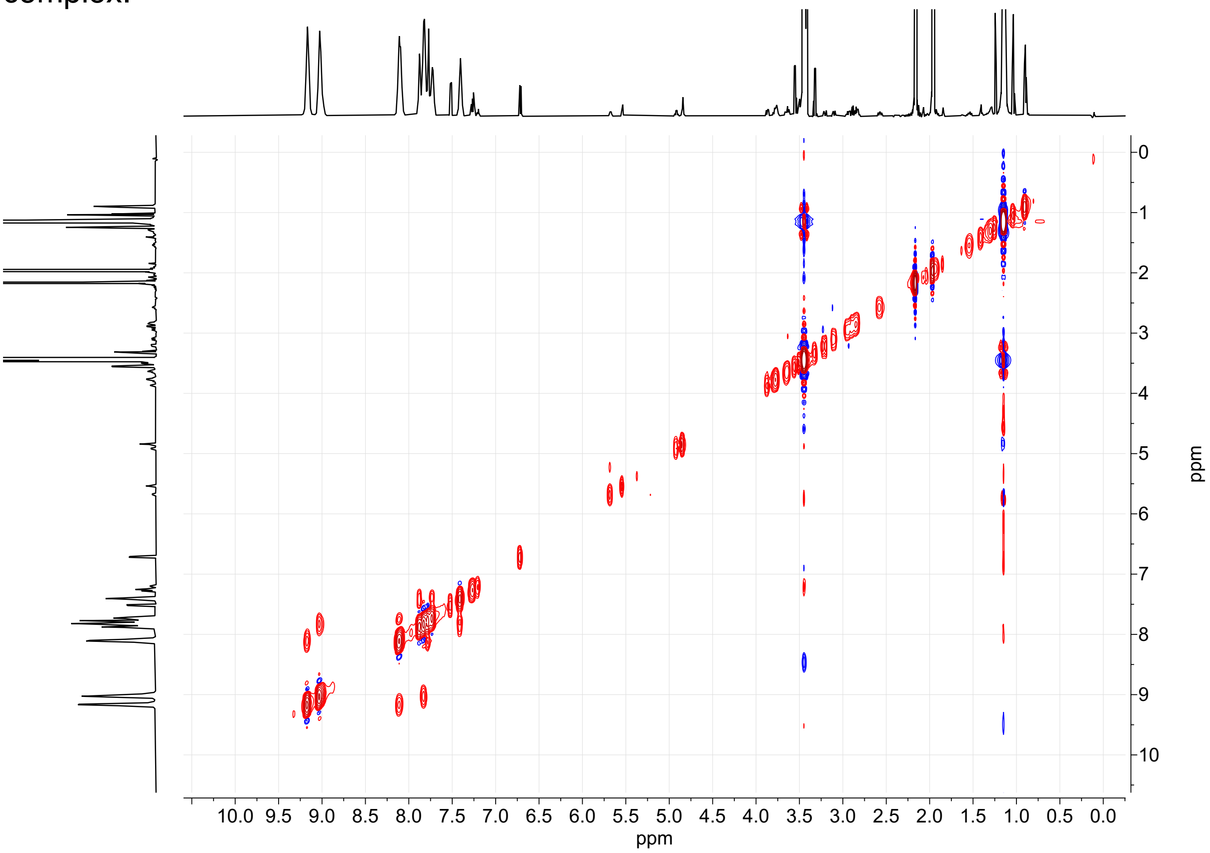

Figure S66. NOSEY NMR (d_3-MeCN, 600 MHz, 298 K) of $Pd_6L_{12}(NTf_2)_{12}$ + Darunavir complex.

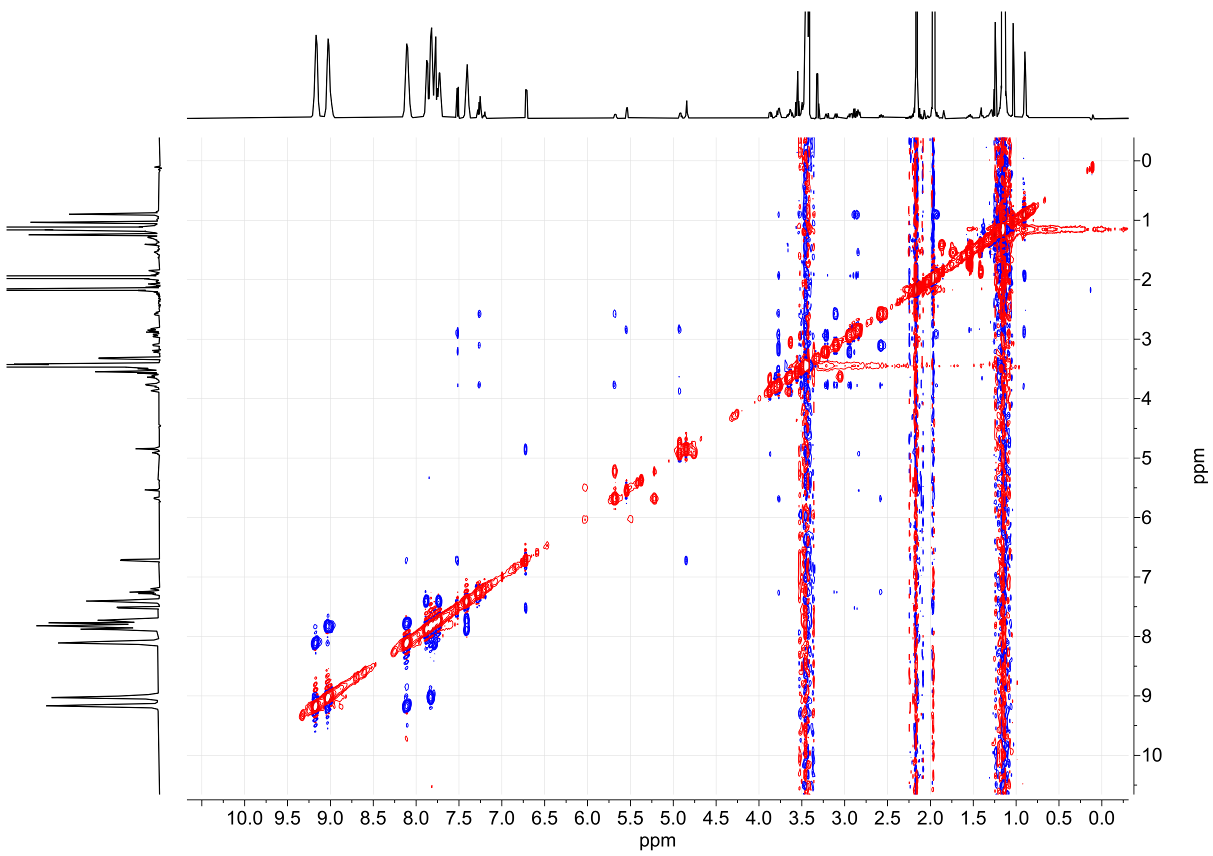

Figure S67. ROSEY NMR (d_3-MeCN, 600 MHz, 298 K) of $Pd_6L_{12}(NTf_2)_{12}$ + Darunavir complex. A potential weak cross-peak at 8.2-6.5 ppm was observed, but wider interactions were not well resolved, likely due to the relatively weak binding of Darunavir in solution.

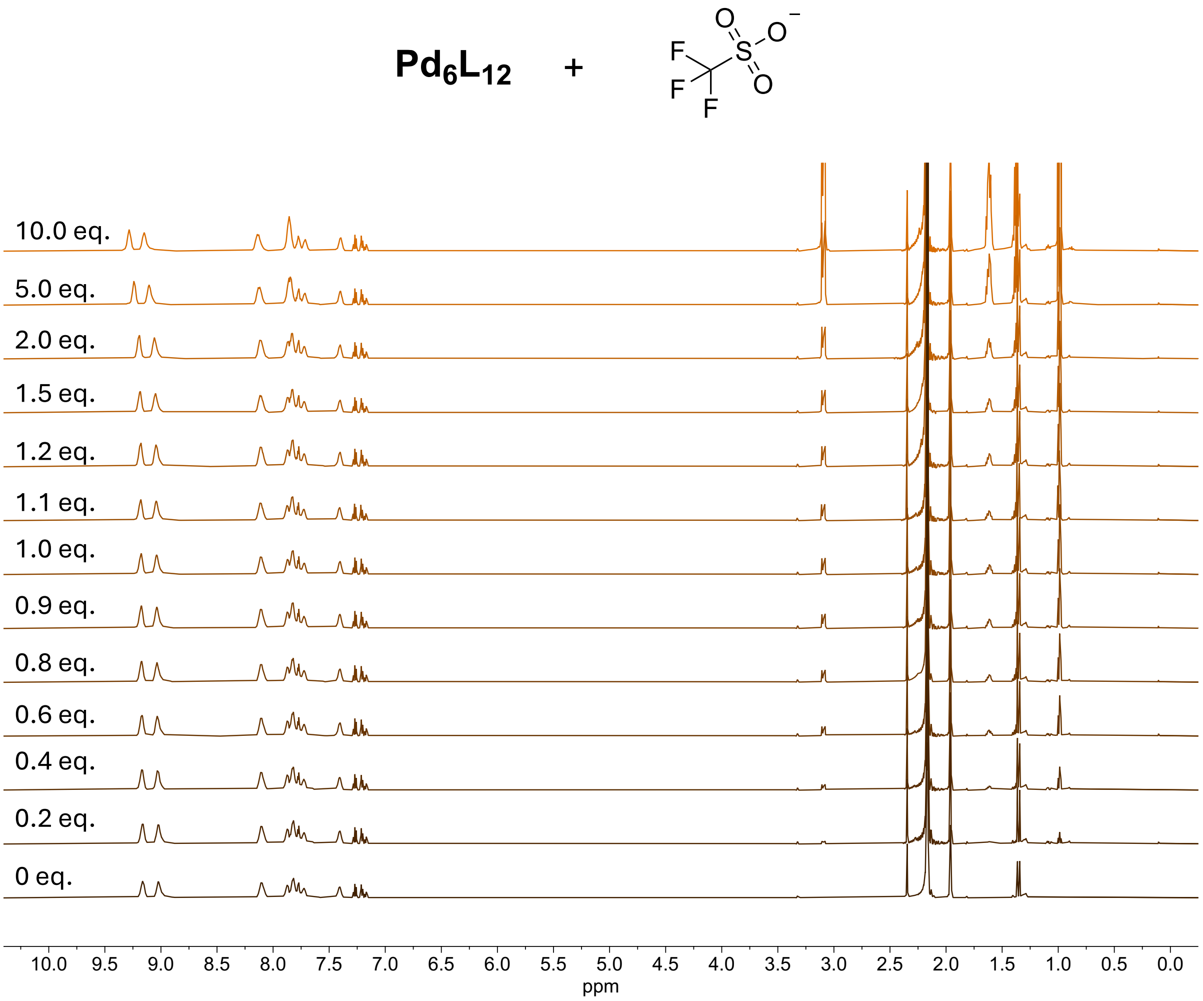

Figure S68. ^{1}H NMR titration (d_3-MeCN, 600 MHz, 298 K) of $Pd_6L_{12}(NTf_2)_{12}$ (0.30 mM) and varying equivalents of OTf^-.

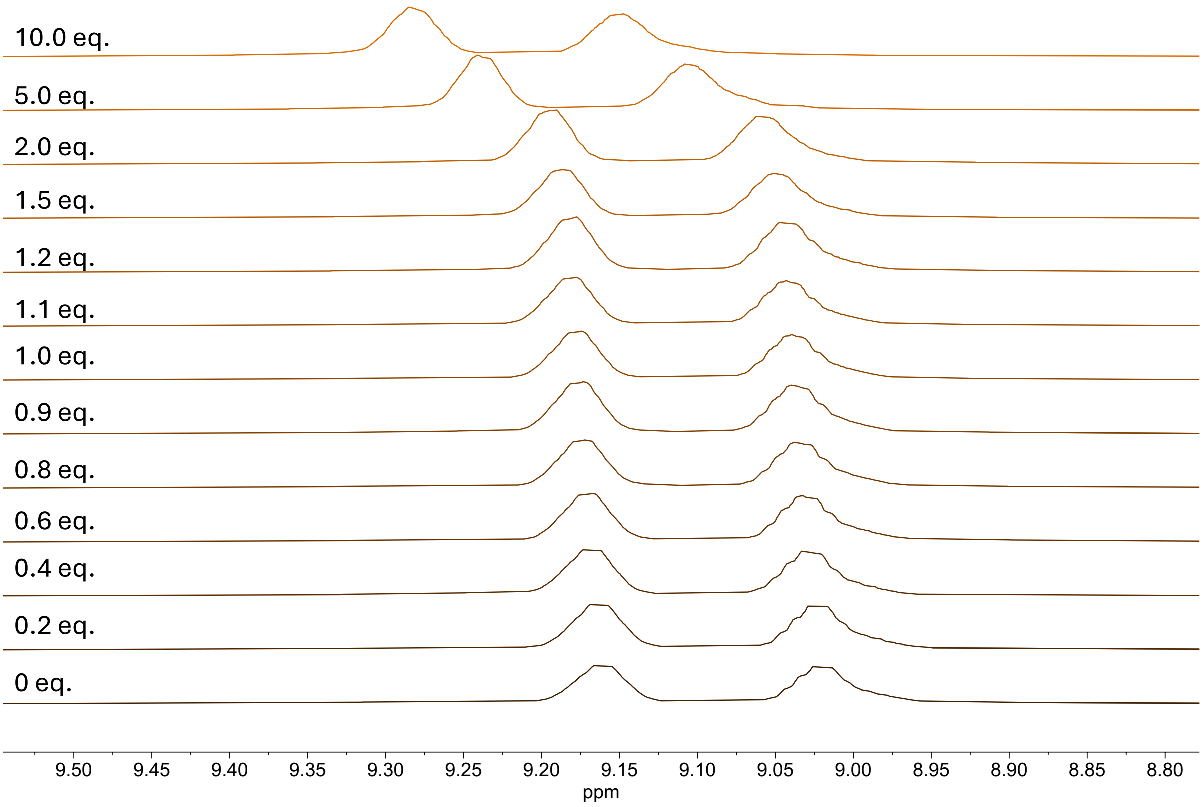

Figure S69. Partial ^{1}H NMR titration (d_3-MeCN, 600 MHz, 298 K) of $Pd_6L_{12}(NTf_2)_{12}$ (0.30 mM) and varying equivalents of OTf^-.

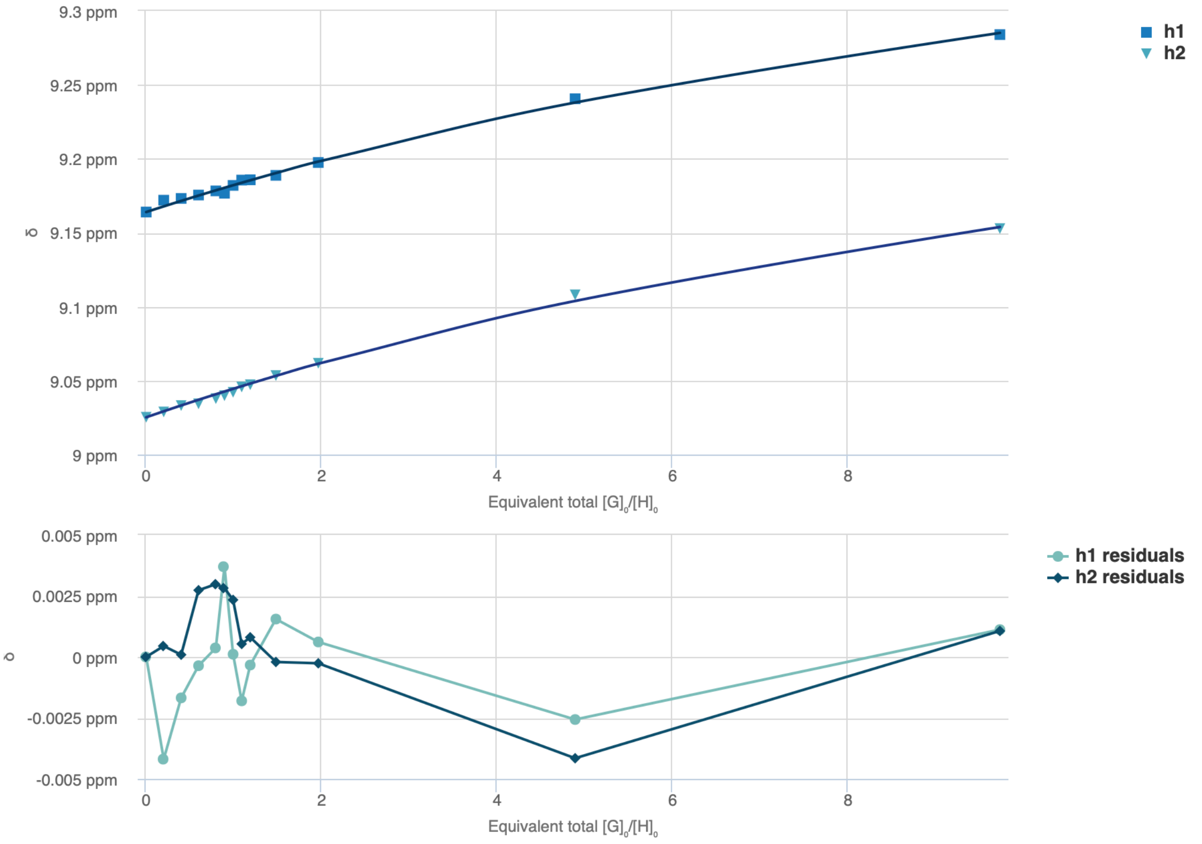

Figure S70. Binding data for $Pd_6L_{12}(NTf_2)_{12}$ and OTf^-, K_a = 207.99 M^{-1}± 2.8% using a 1:1 model in Bindfit.

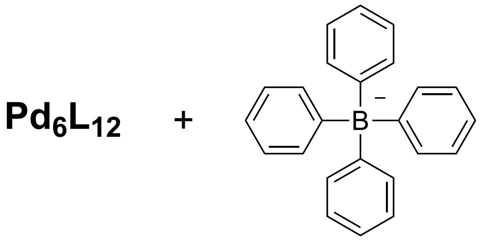

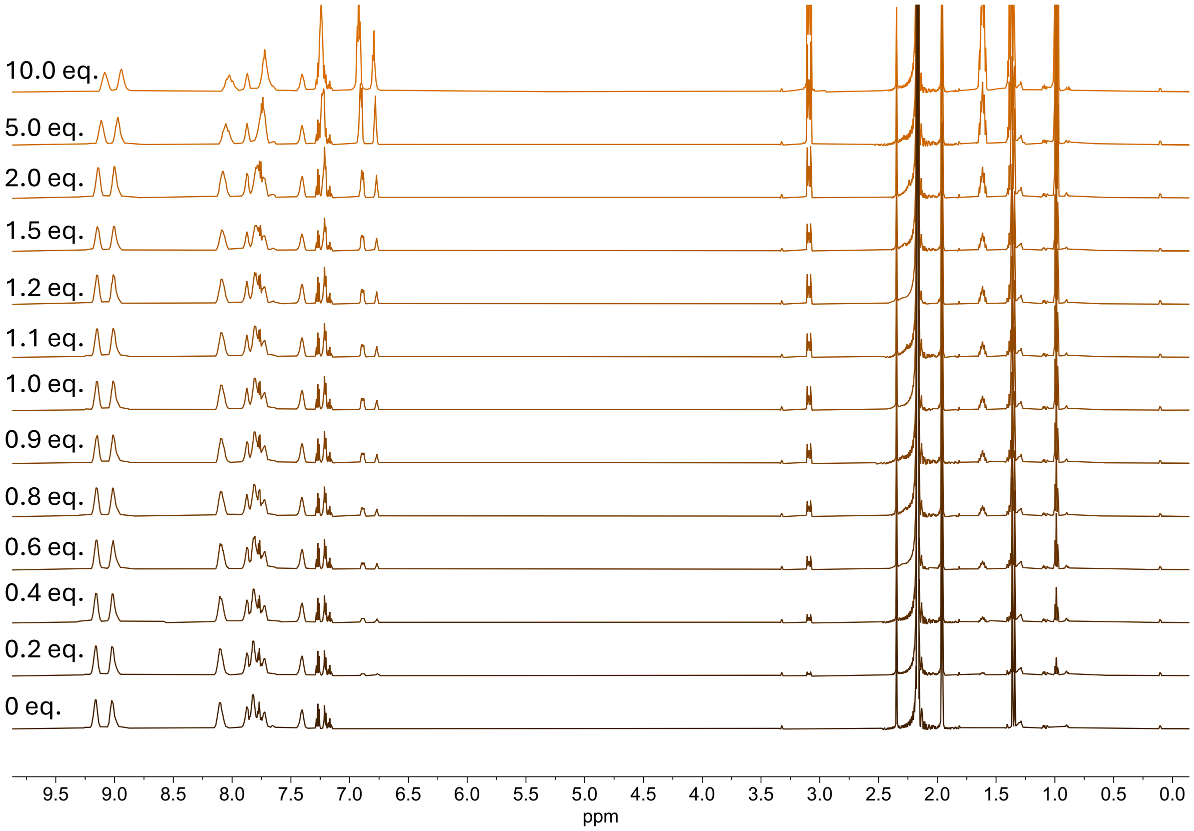

Figure S71. ^{1}H NMR titration (d_3-MeCN, 600 MHz, 298 K) of $Pd_6L_{12}(NTf_2)_{12}$ (0.30 mM) and varying equivalents of BPh_4^-.

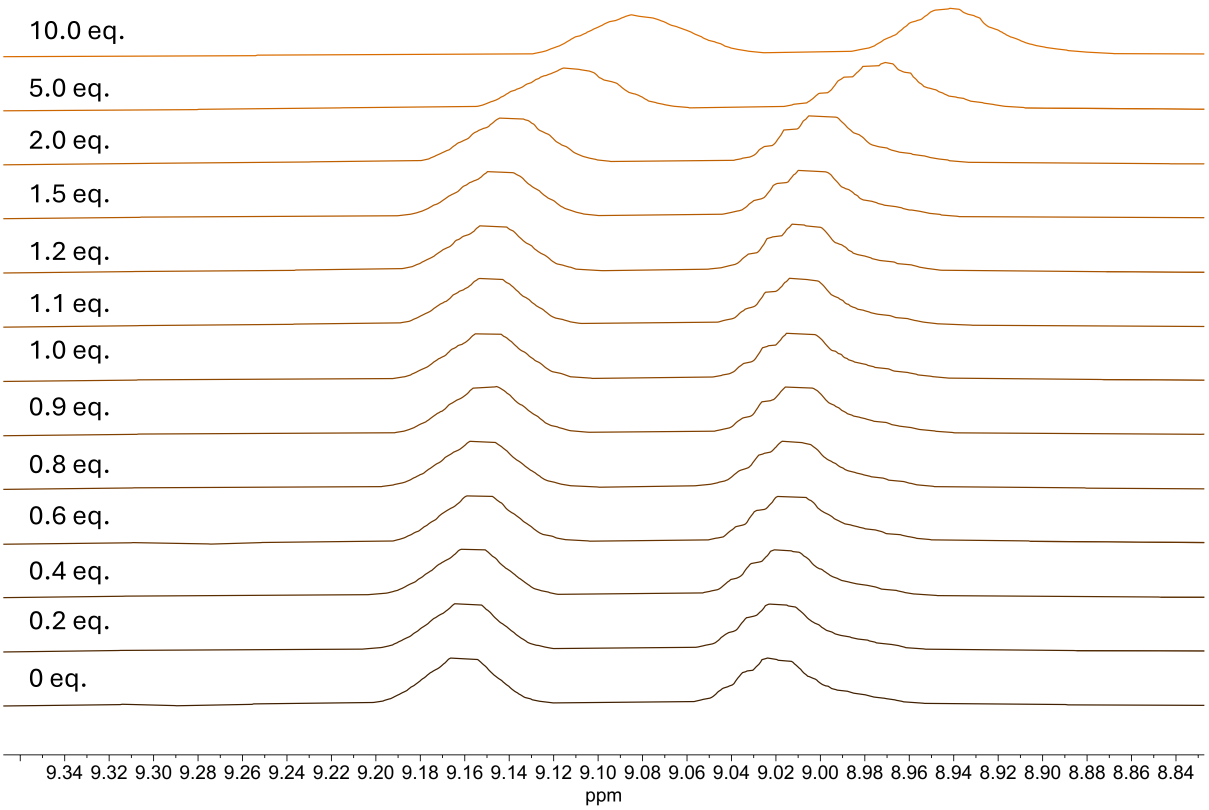

Figure S72. Partial ^1H NMR titration (d_3-MeCN, 600 MHz, 298 K) of $Pd_6L_{12}(NTf_2)_{12}$ (0.30 mM) and varying equivalents of BPh_4^-.

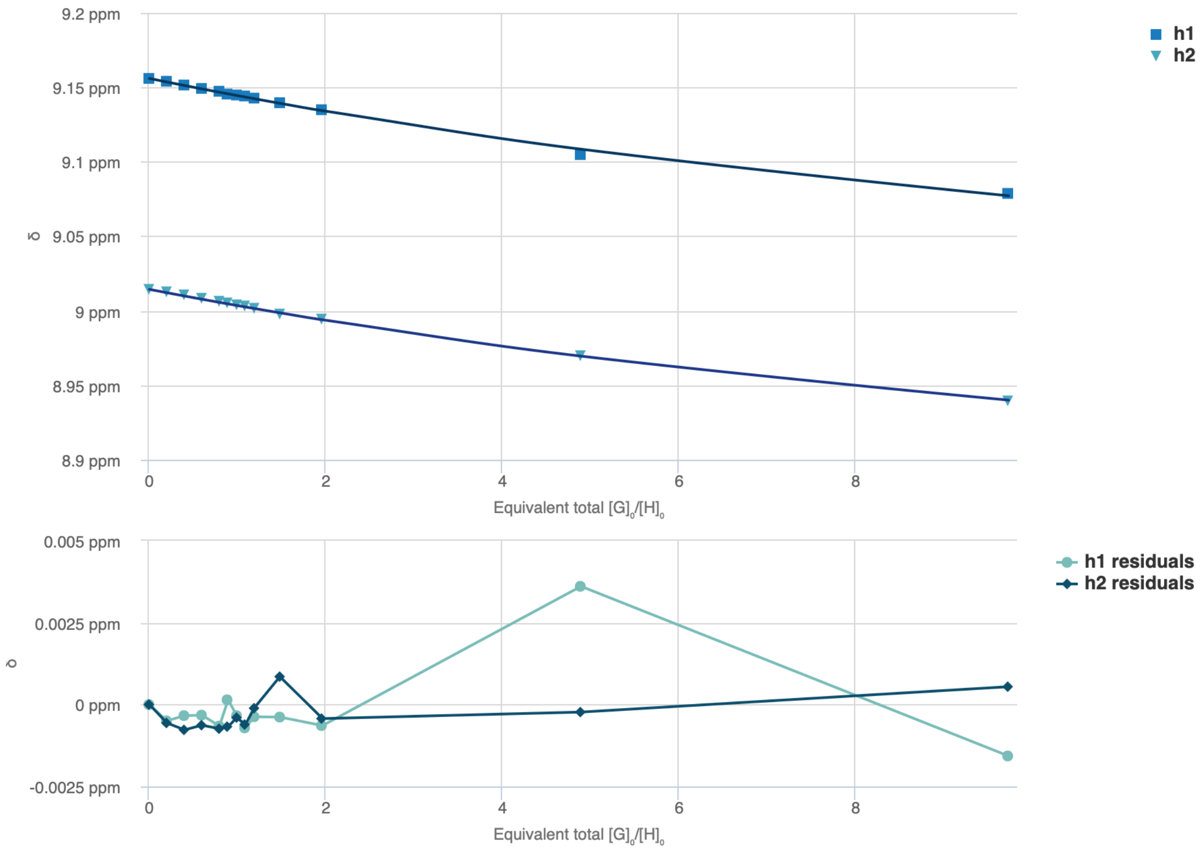

Figure S73. Binding data for $Pd_6L_{12}(NTf_2)_{12}$ and BPh_4^-, $K_a = 186.84\ M^{-1} \pm 2.1\%$.

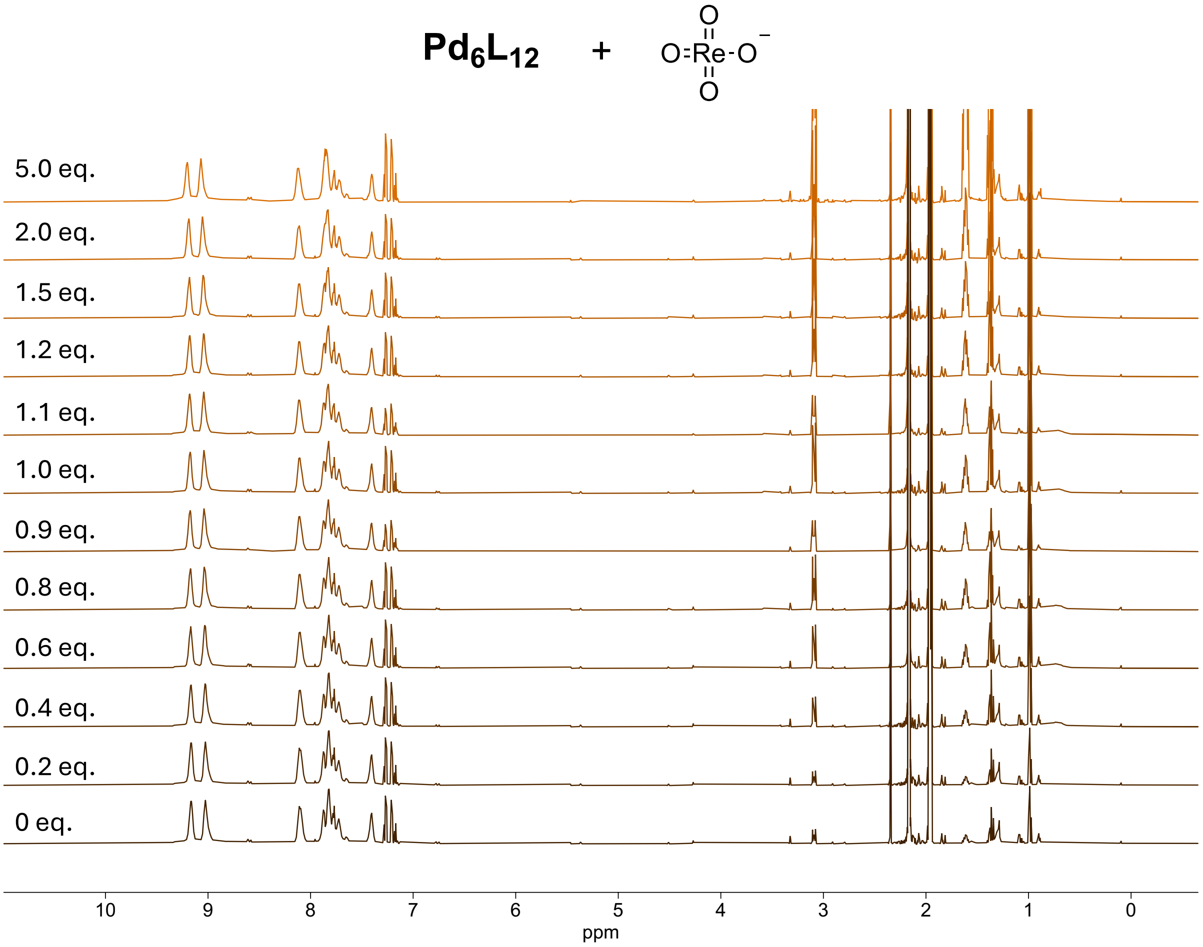

Figure S74. ^{1}H NMR titration (d_3-MeCN, 600 MHz, 298 K) of $Pd_6L_{12}(NTf_2)_{12}$ (0.30 mM) and varying equivalents of ReO_4^-.

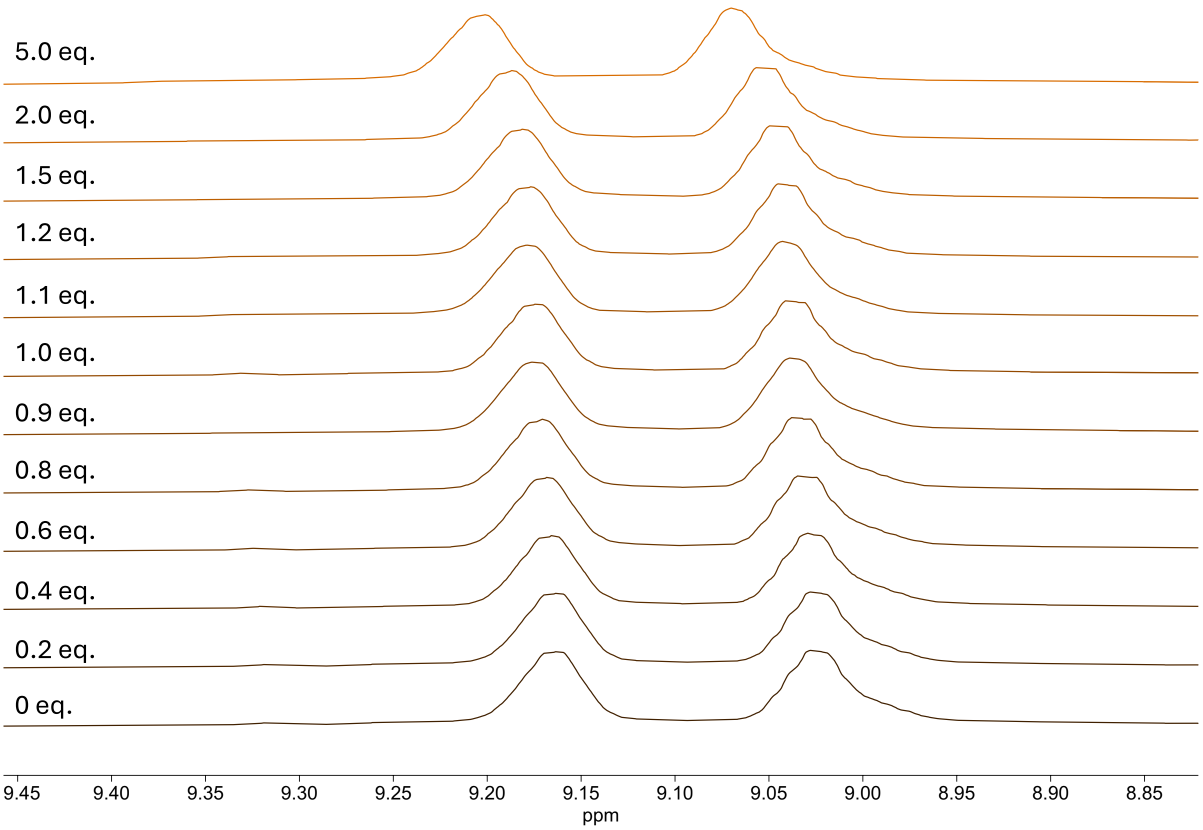

Figure S75. Partial ^{1}H NMR titration (d_3-MeCN, 600 MHz, 298 K) of $Pd_6L_{12}(NTf_2)_{12}$ (0.30 mM) and varying equivalents of ReO_4^-.

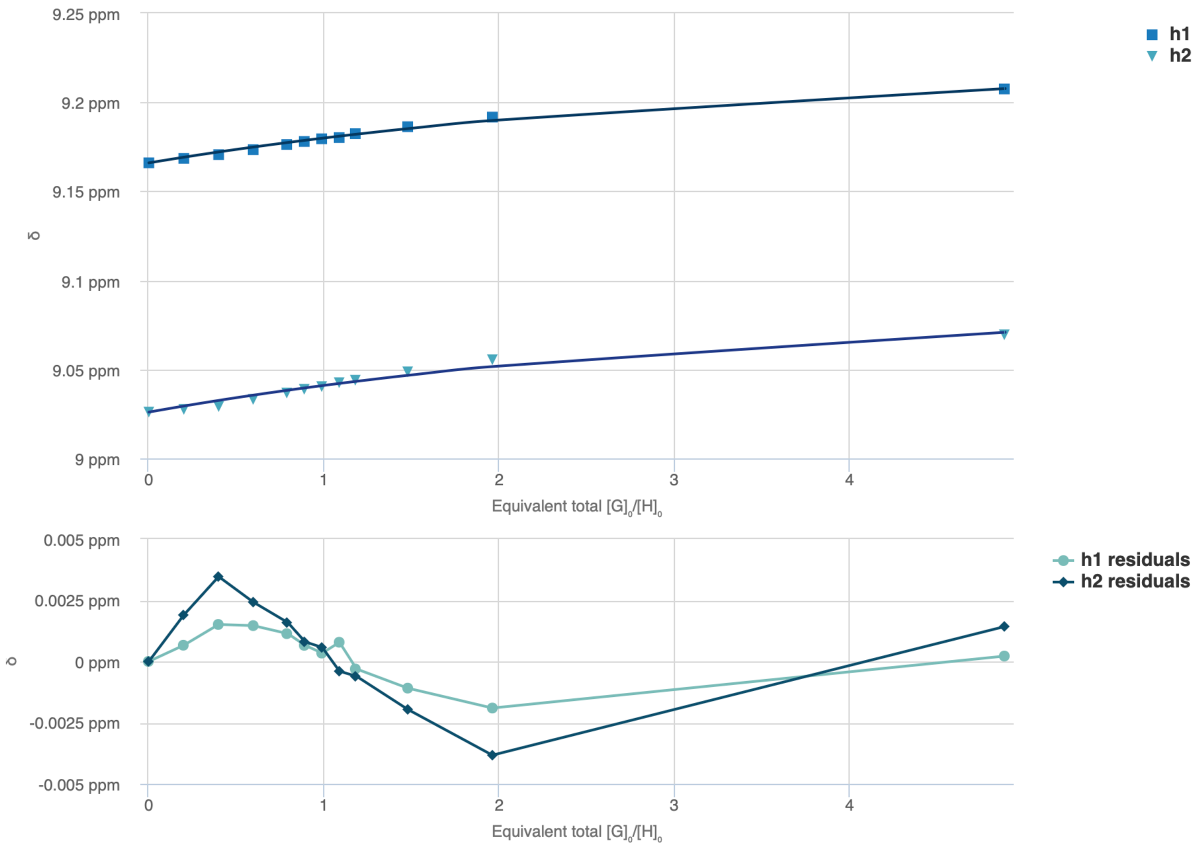

Figure S76. Binding data for $Pd_6L_{12}(NTf_2)_{12}$ and ReO_4^-, K_a = 891.83 M^{-1} ± 6.9%.

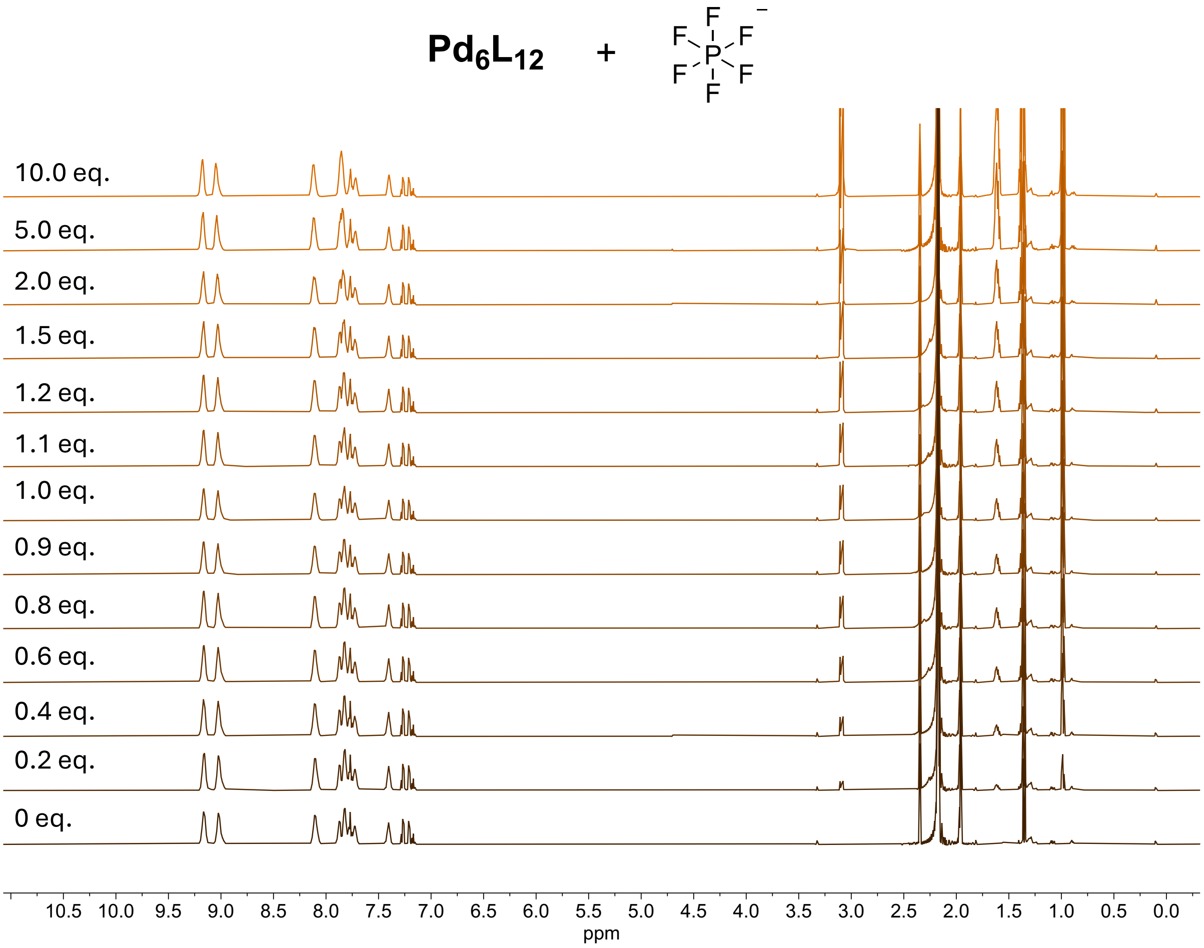

Figure S77. ^1H NMR titration (d_3-MeCN, 600 MHz, 298 K) of $Pd_6L_{12}(NTf_2)_{12}$ (0.30 mM) and varying equivalents of PF_6^-.

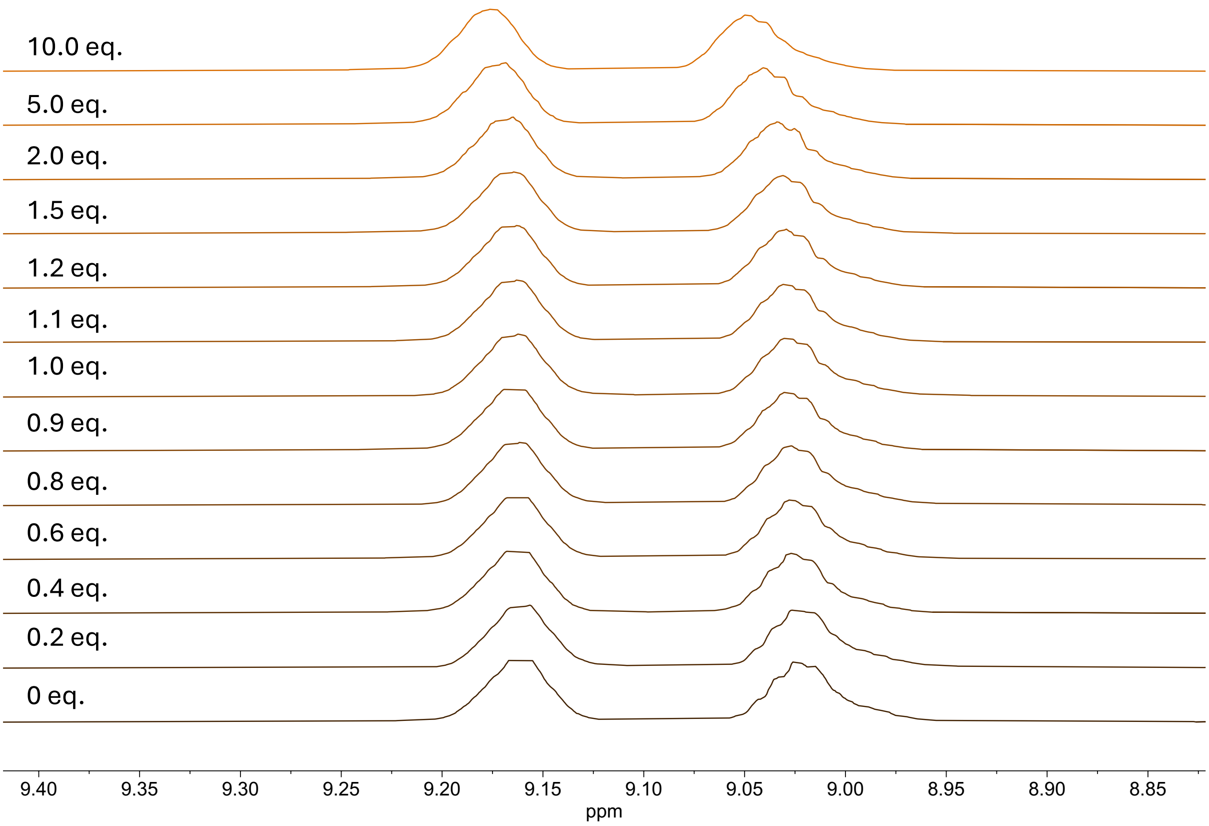

Figure S78. Partial ^{1}H NMR titration (d_3-MeCN, 600 MHz, 298 K) of $Pd_6L_{12}(NTf_2)_{12}$ (0.30 mM) and varying equivalents of PF_6^-.

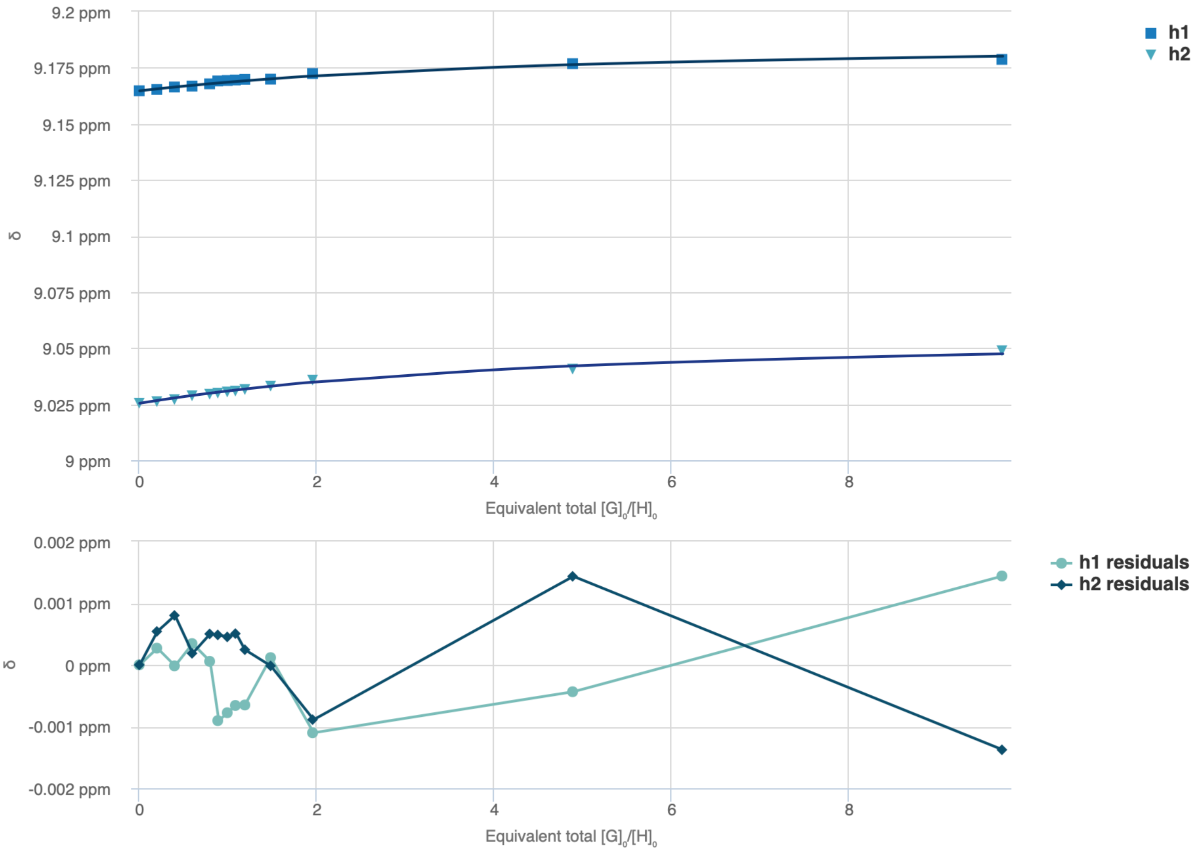

Figure S79. Binding data for $Pd_6L_{12}(NTf_2)_{12}$ and PF_6^-, K_a = 851.54 M^{-1} ± 8.3%.

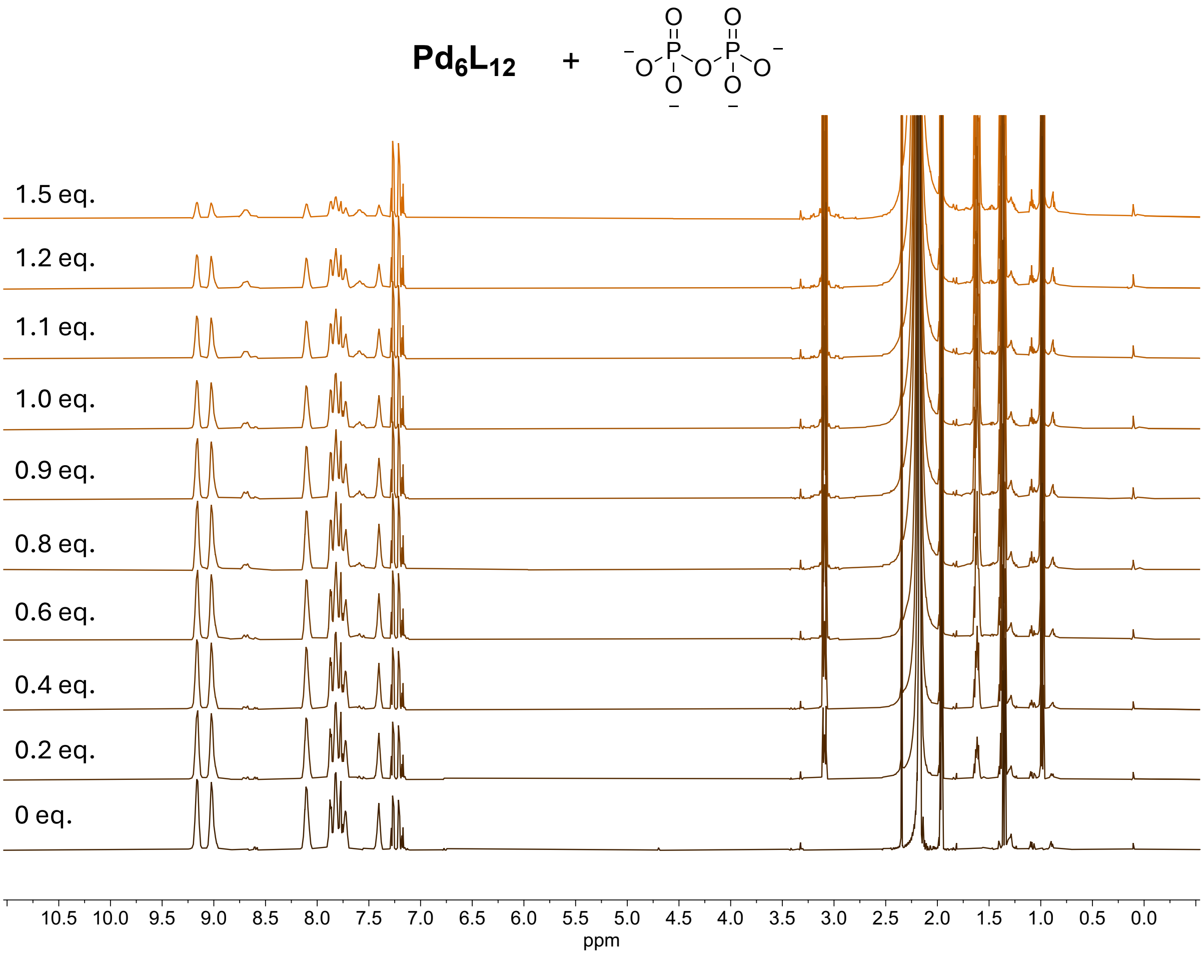

Figure S80. ^{1}H NMR titration (d_3-MeCN, 600 MHz, 298 K) of $Pd_6L_{12}(NTf_2)_{12}$ (0.30 mM) and varying equivalents of $P_2O_7^{4-}$.

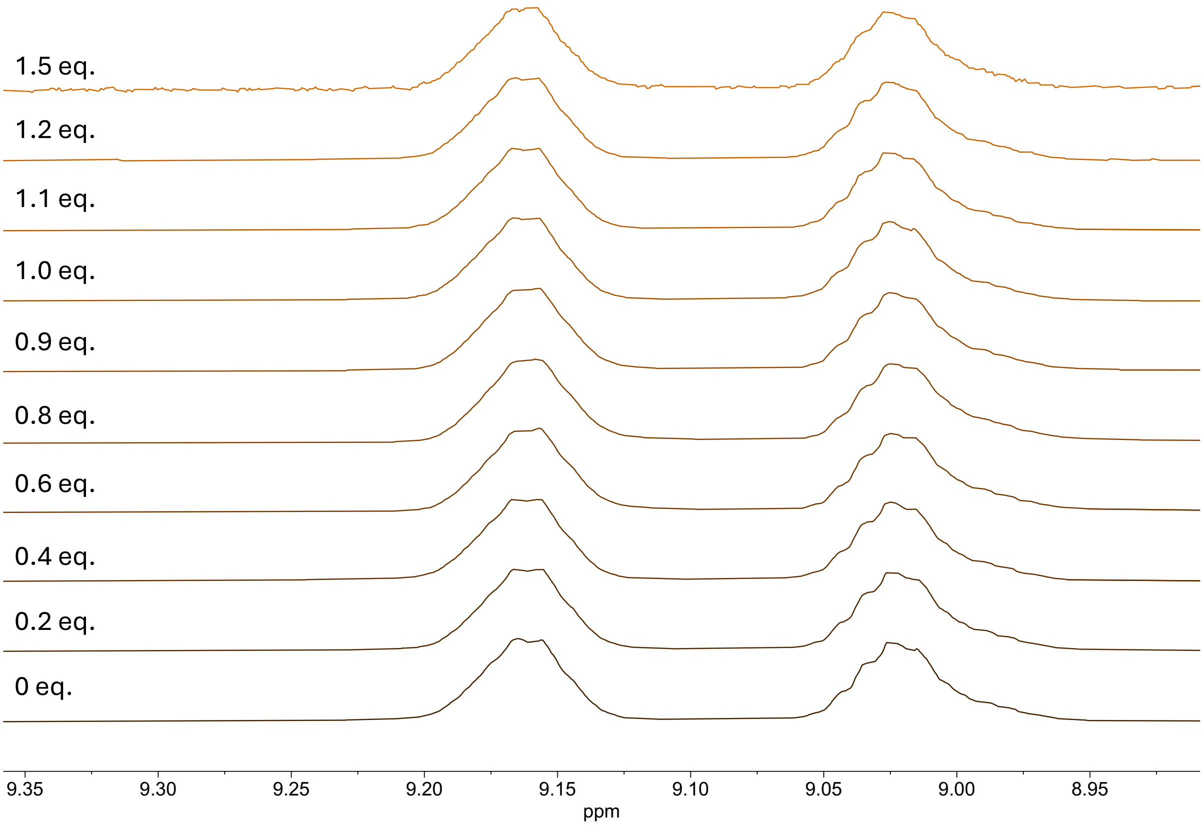

Figure S81. Partial ^{1}H NMR titration (d_3-MeCN, 600 MHz, 298 K) of $Pd_6L_{12}(NTf_2)_{12}$ (0.30 mM) and varying equivalents of $P_2O_7^{4-}$.

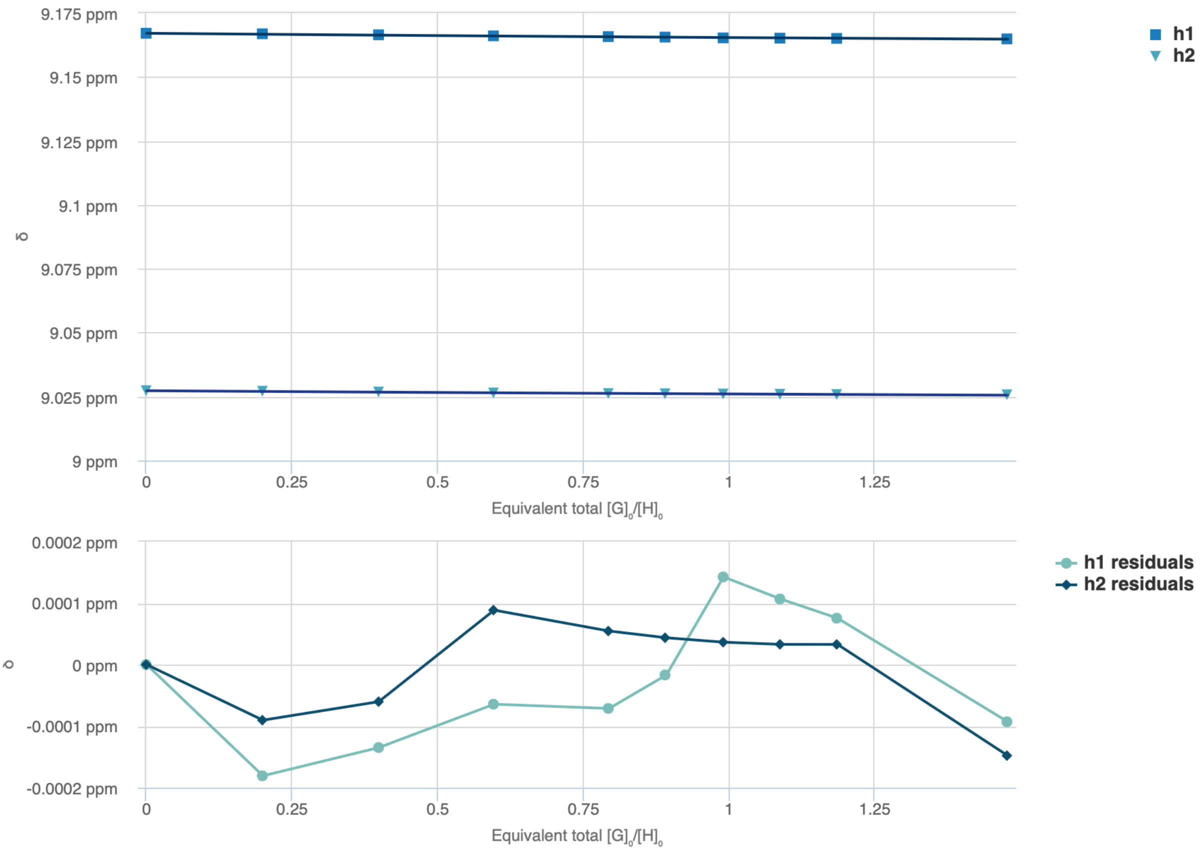

Figure S82. Binding data for $Pd_6L_{12}(NTf_2)_{12}$ and $P_2O_7^{4-}$, K_a = 962.33 M^{-1} ± 5.4%.

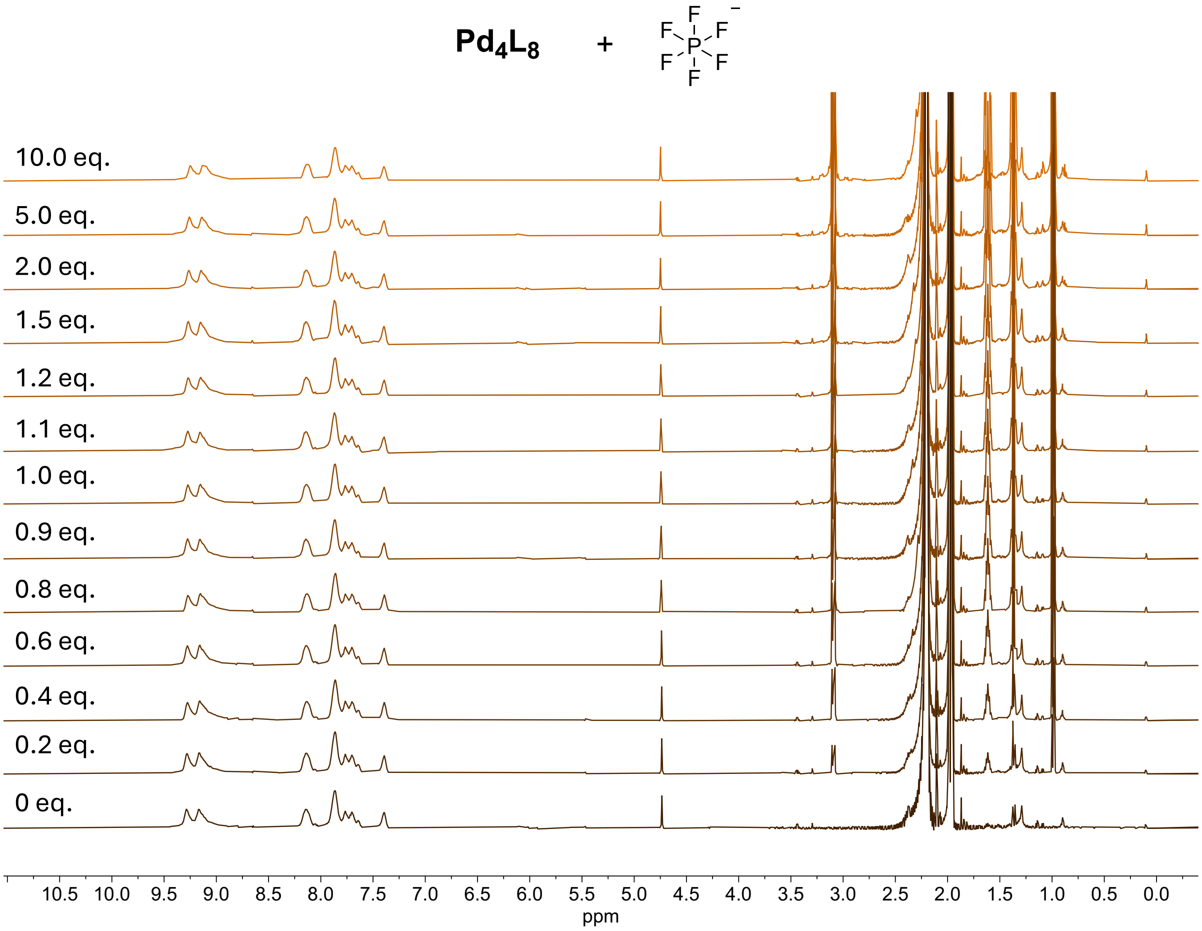

Figure S83. ^{1}H NMR titration (d_3-MeCN, 600 MHz, 298 K) of $Pd_4L_8(BF_4)_8$ (0.50 mM) and varying equivalents of PF_6^-.

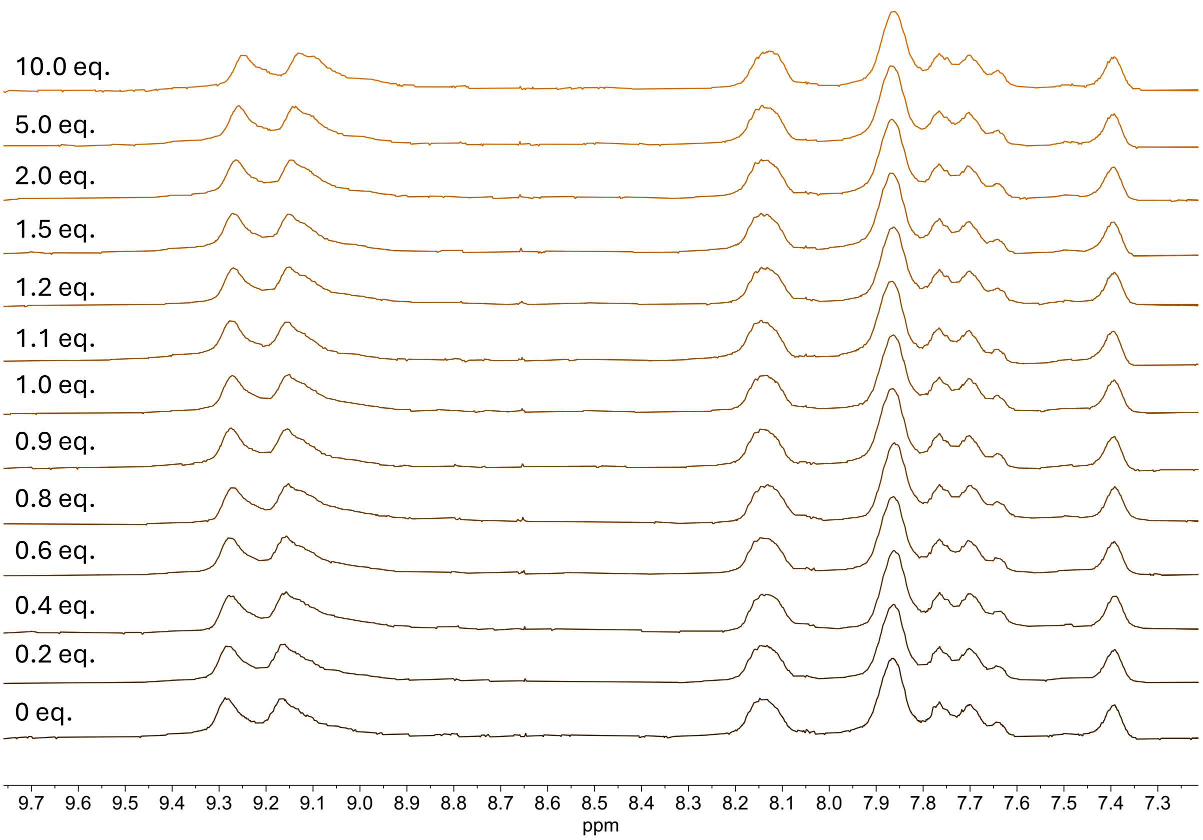

Figure S84. Partial ^{1}H NMR titration (d_3-MeCN, 600 MHz, 298 K) of $Pd_4L_8(BF_4)_8$ (0.50 mM) and varying equivalents of PF_6^-.

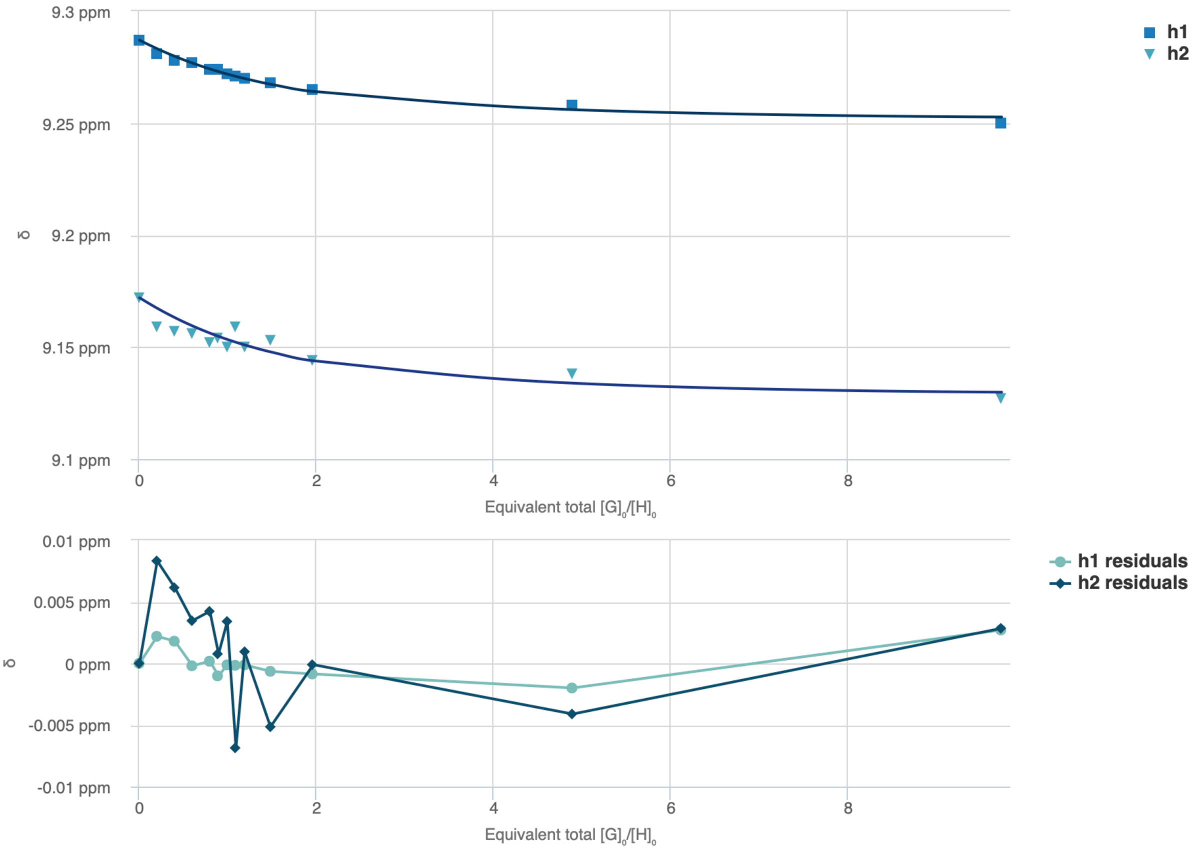

Figure S85. Binding data for $Pd_4L_8(BF_4)_8$ and PF_6^-, K_a = 962.33 M^{-1} ± 5.4%.

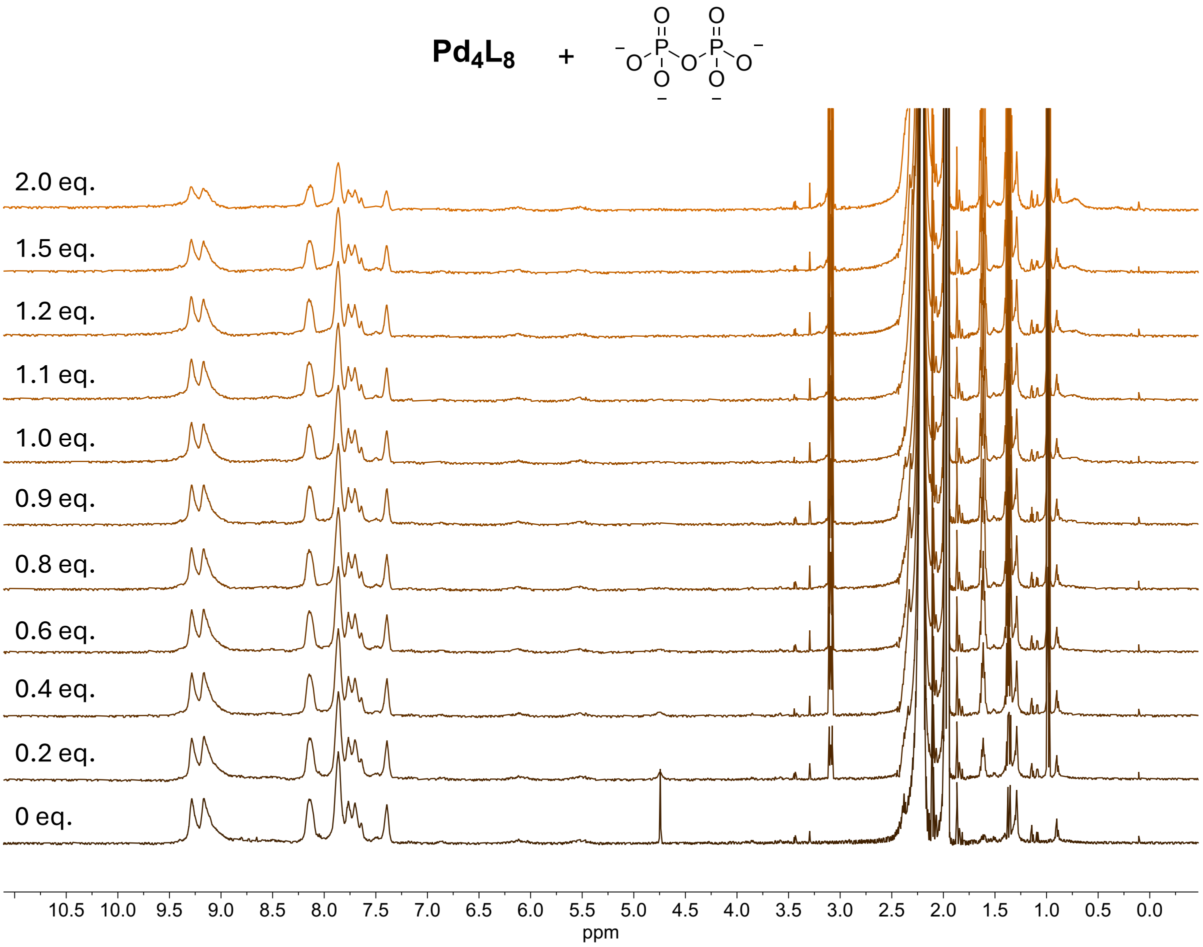

Figure S86. ^{1}H NMR titration (d_3-MeCN, 600 MHz, 298 K) of $Pd_4L_8(BF_4)_8$ (0.50 mM) and varying equivalents of $P_2O_7^{4-}$.

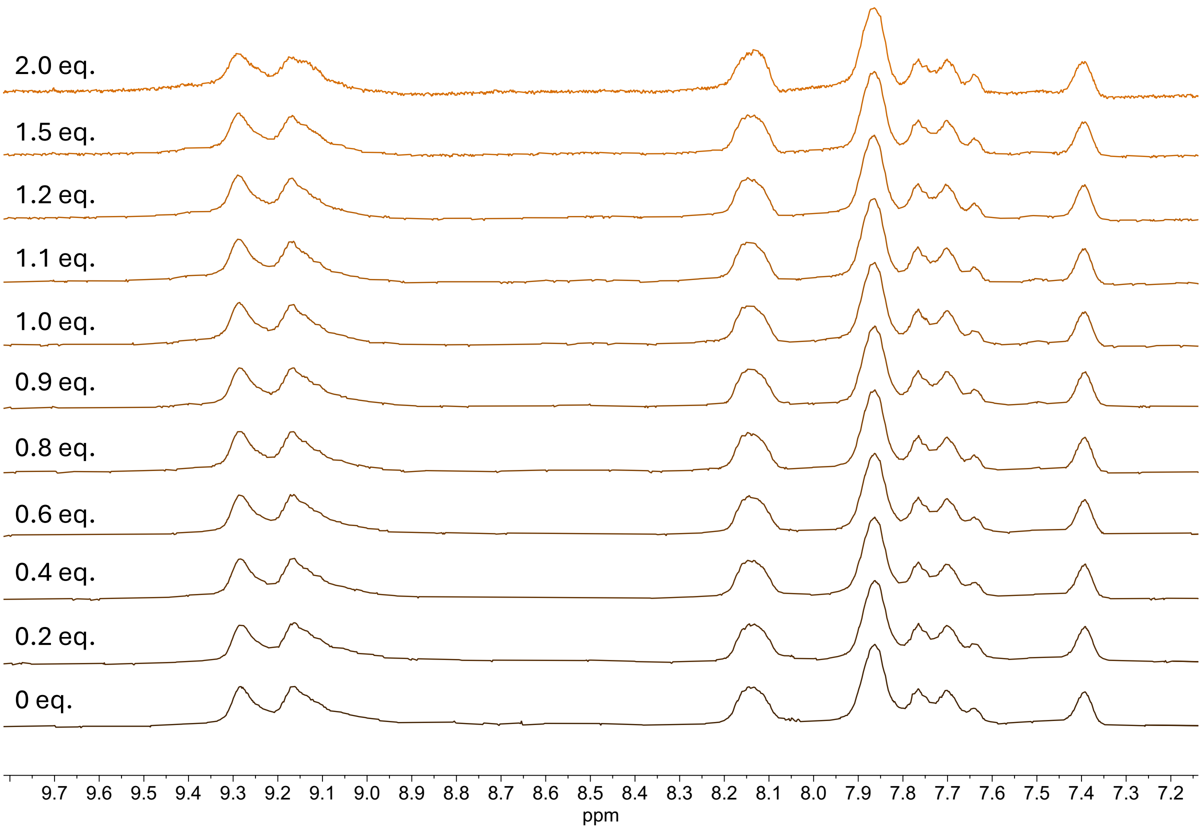

Figure S87. Partial ^{1}H NMR titration (*d3*-MeCN, 600 MHz, 298 K) of $Pd_4L_8(BF_4)_8$ (0.50 mM) and varying equivalents of $P_2O_7^{4-}$.

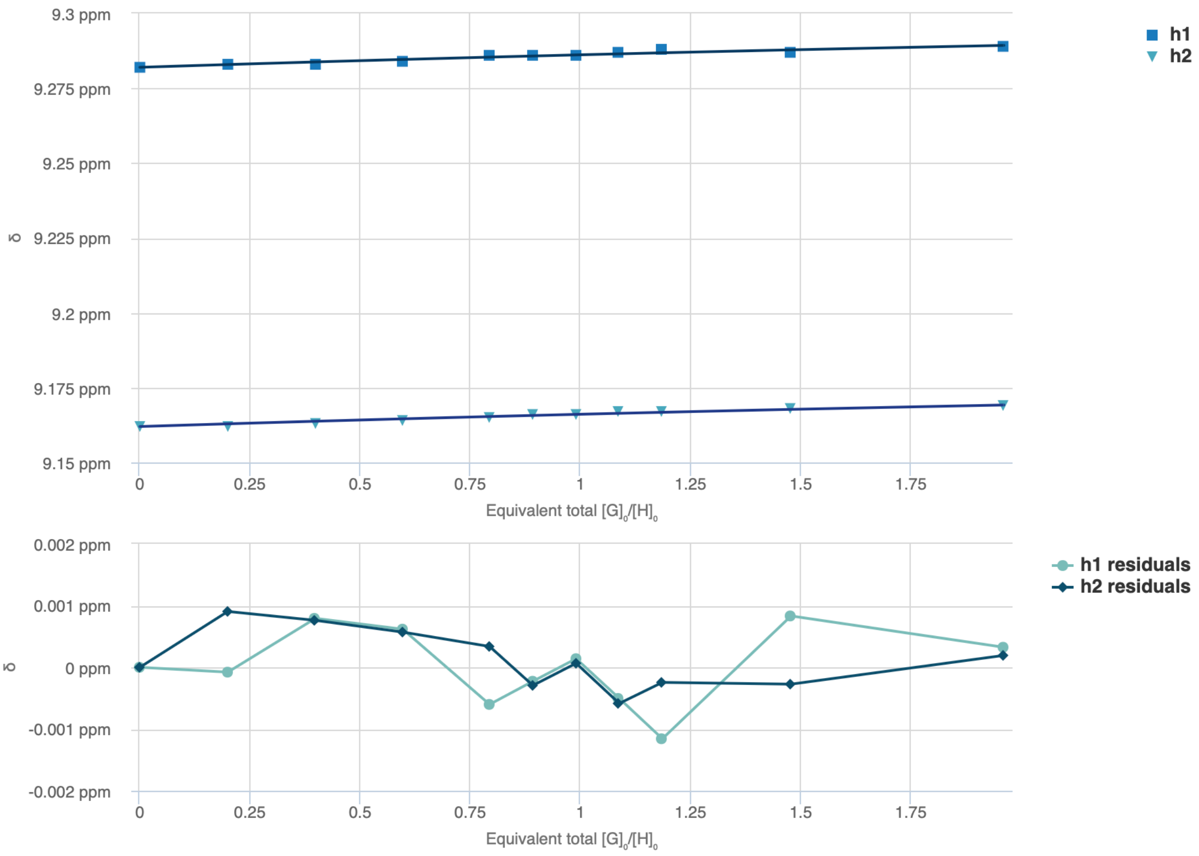

Figure S88. Binding data for $Pd_4L_8(BF_4)_8$ and $P_2O_7^{4-}$, K_a = 616.34 M^{-1} ± 8.8%.

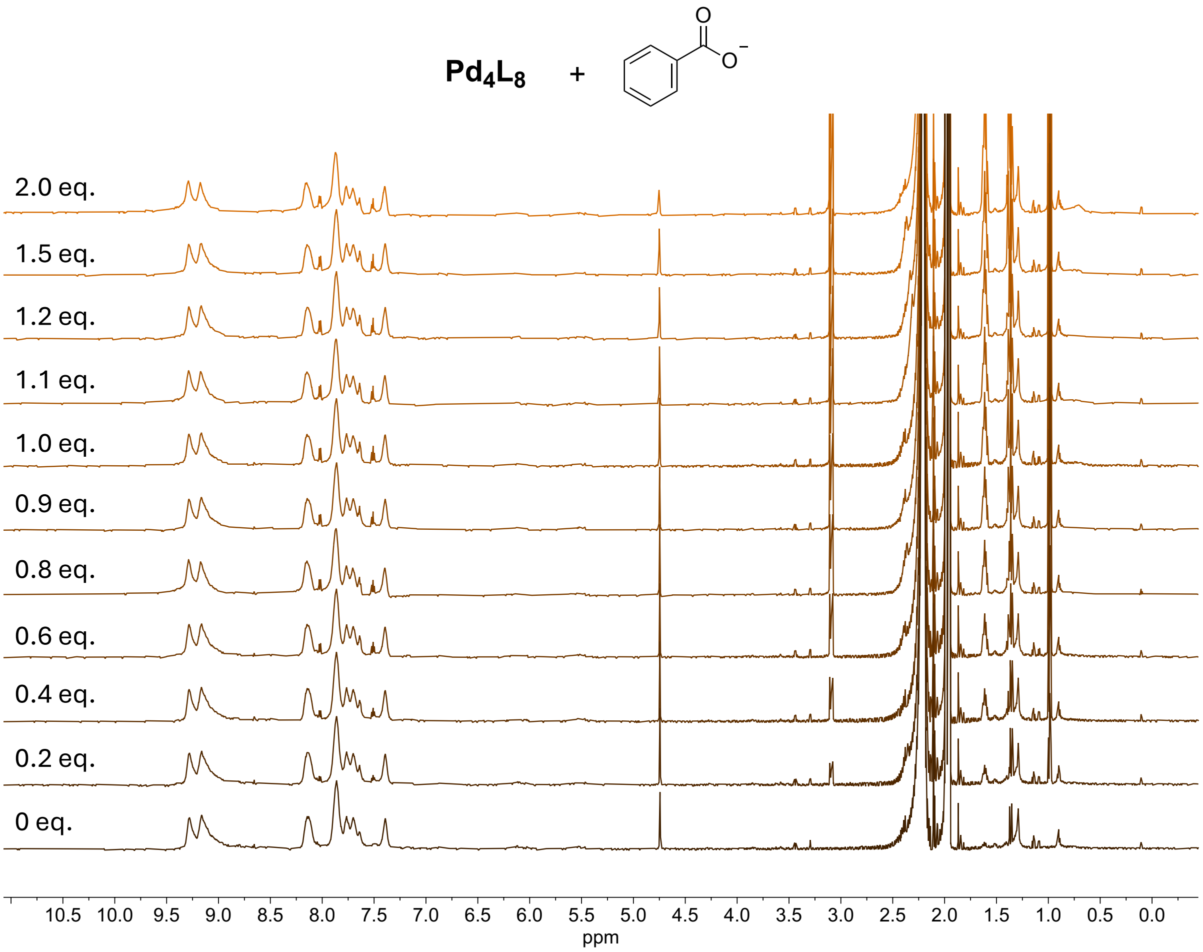

Figure S89. ^{1}H NMR titration (d_3-MeCN, 600 MHz, 298 K) of $Pd_4L_8(BF_4)_8$ (0.50 mM) and varying equivalents of benzoate.

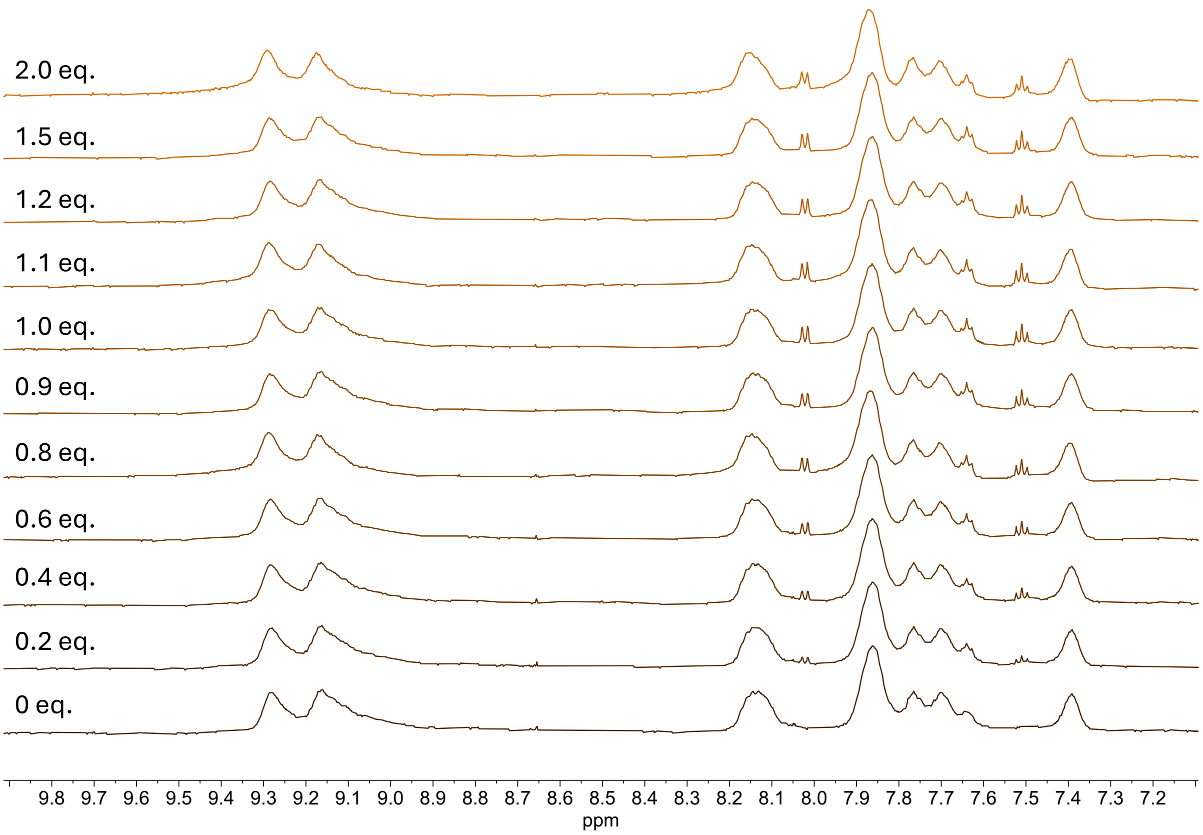

Figure S90. Partial ^{1}H NMR titration (d_3-MeCN, 600 MHz, 298 K) of $Pd_4L_8(BF_4)_8$ (0.50 mM) and varying equivalents of benzoate.

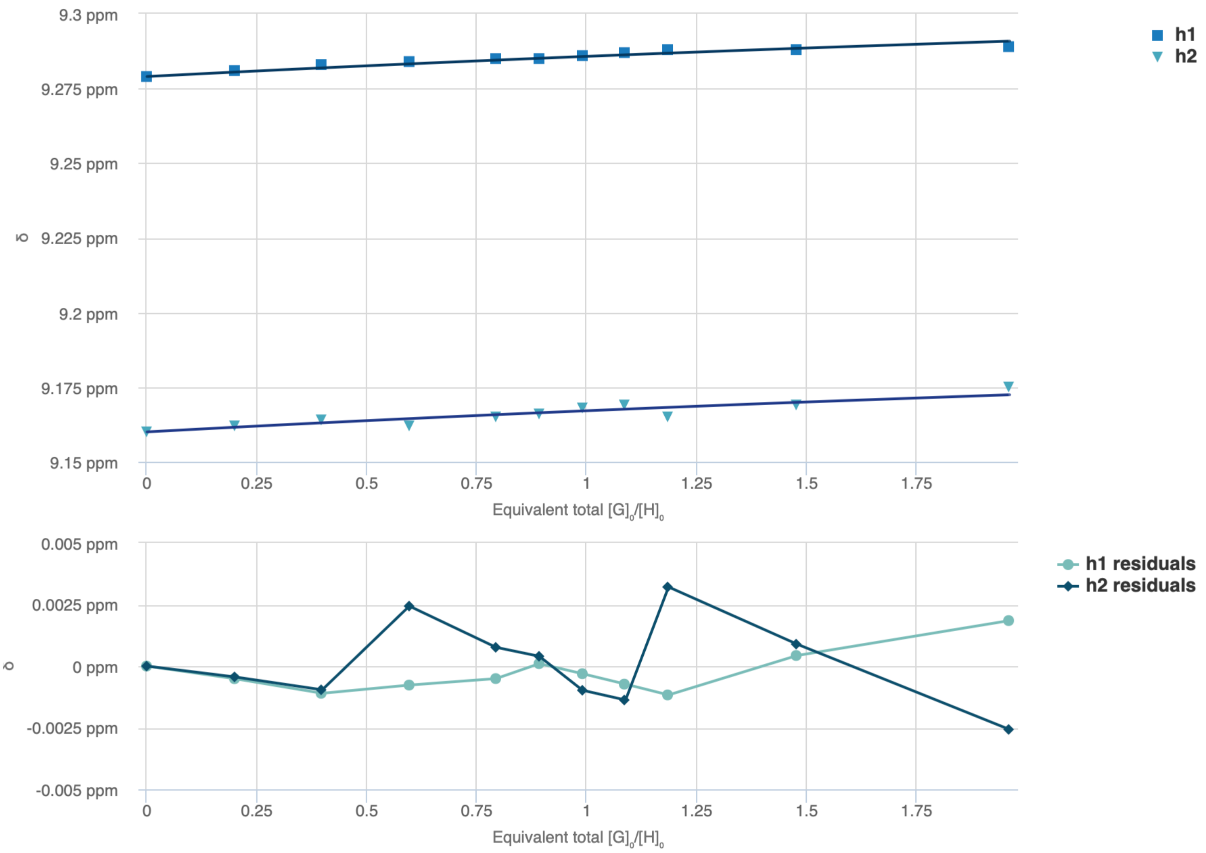

Figure S91. Binding data for $Pd_4L_8(BF_4)_8$ and benzoate, K_a = 610.65 M^{-1} ± 12.7%.

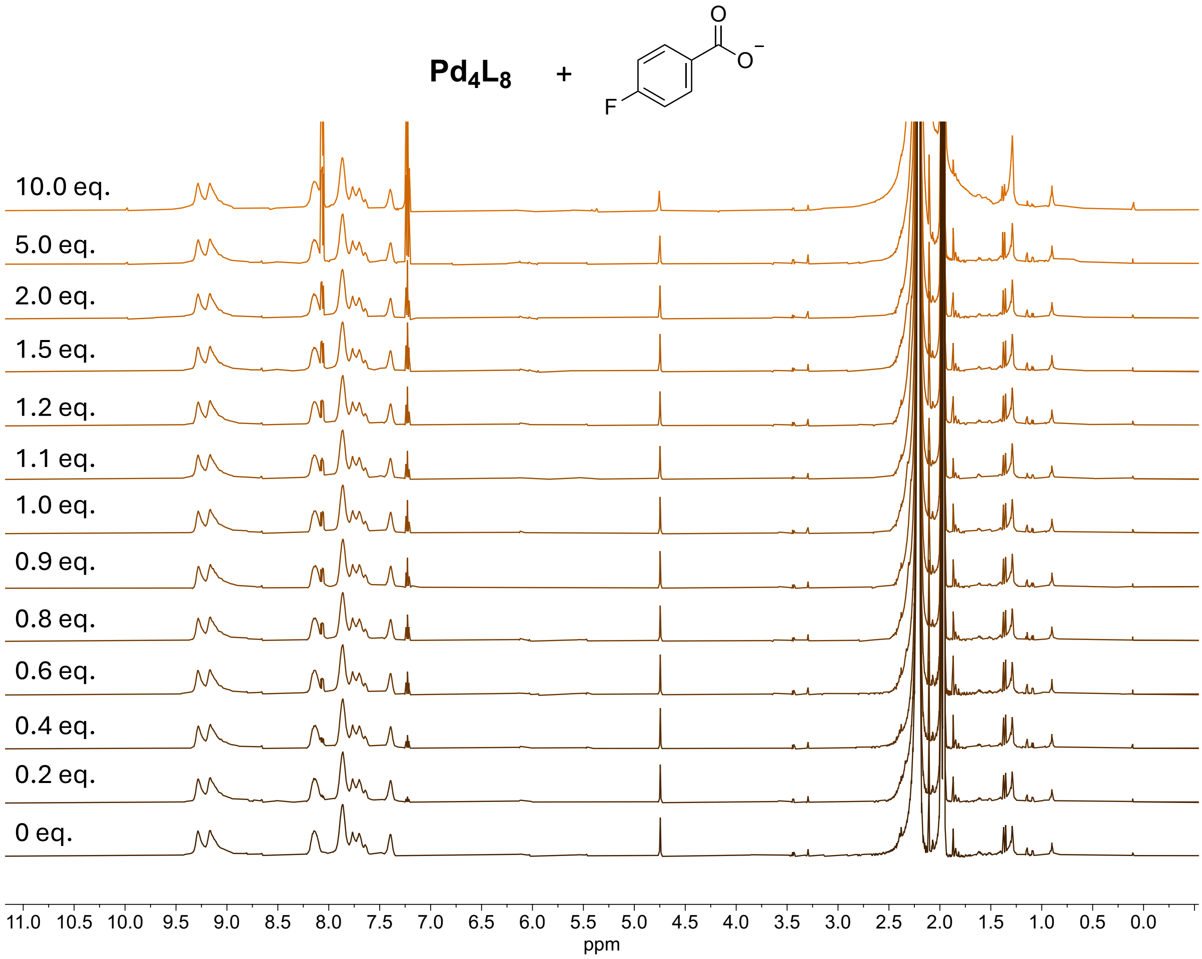

Figure S92. ^{1}H NMR titration (*d*$_3$-MeCN, 600 MHz, 298 K) of $Pd_4L_8(BF_4)_8$ (0.50 mM) and varying equivalents of 4F-benzoate.

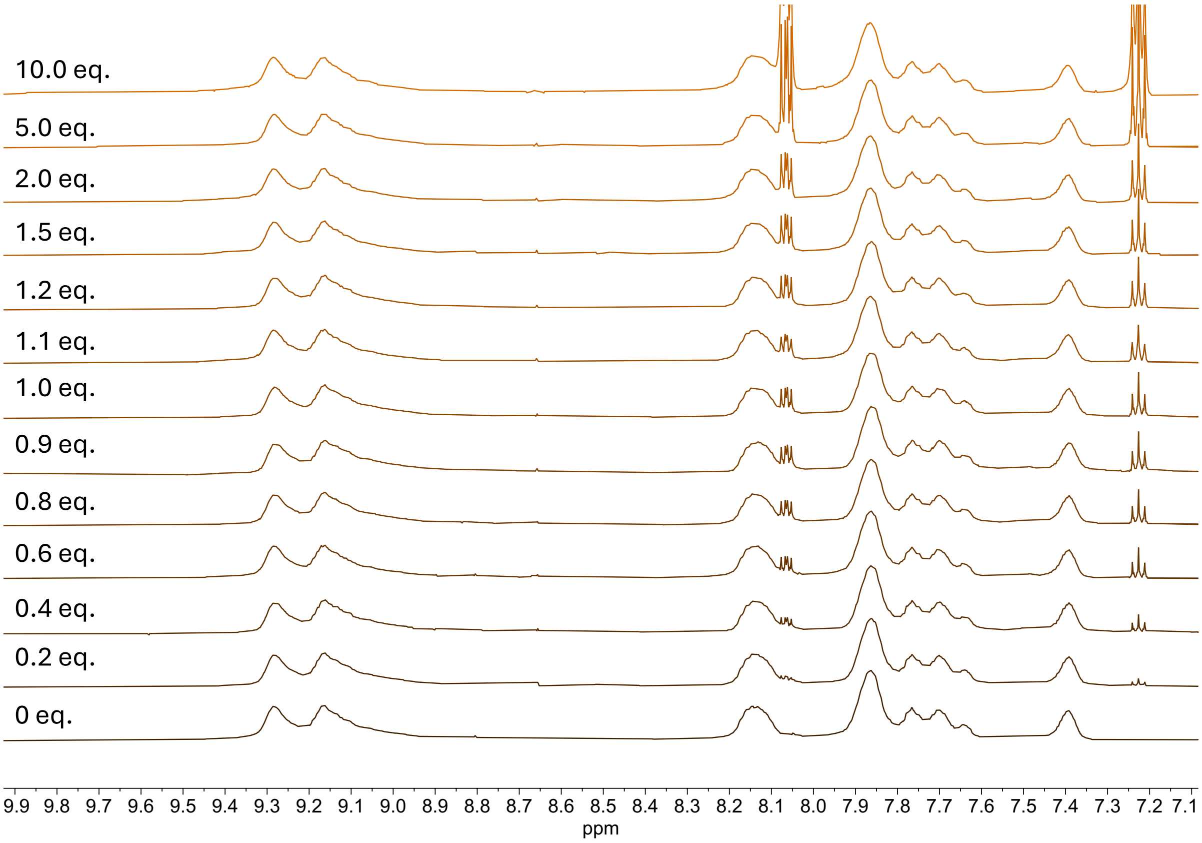

Figure S93. Partial ^{1}H NMR titration (d_3-MeCN, 600 MHz, 298 K) of $Pd_4L_8(BF_4)_8$ (0.50 mM) and varying equivalents of 4F-benzoate.

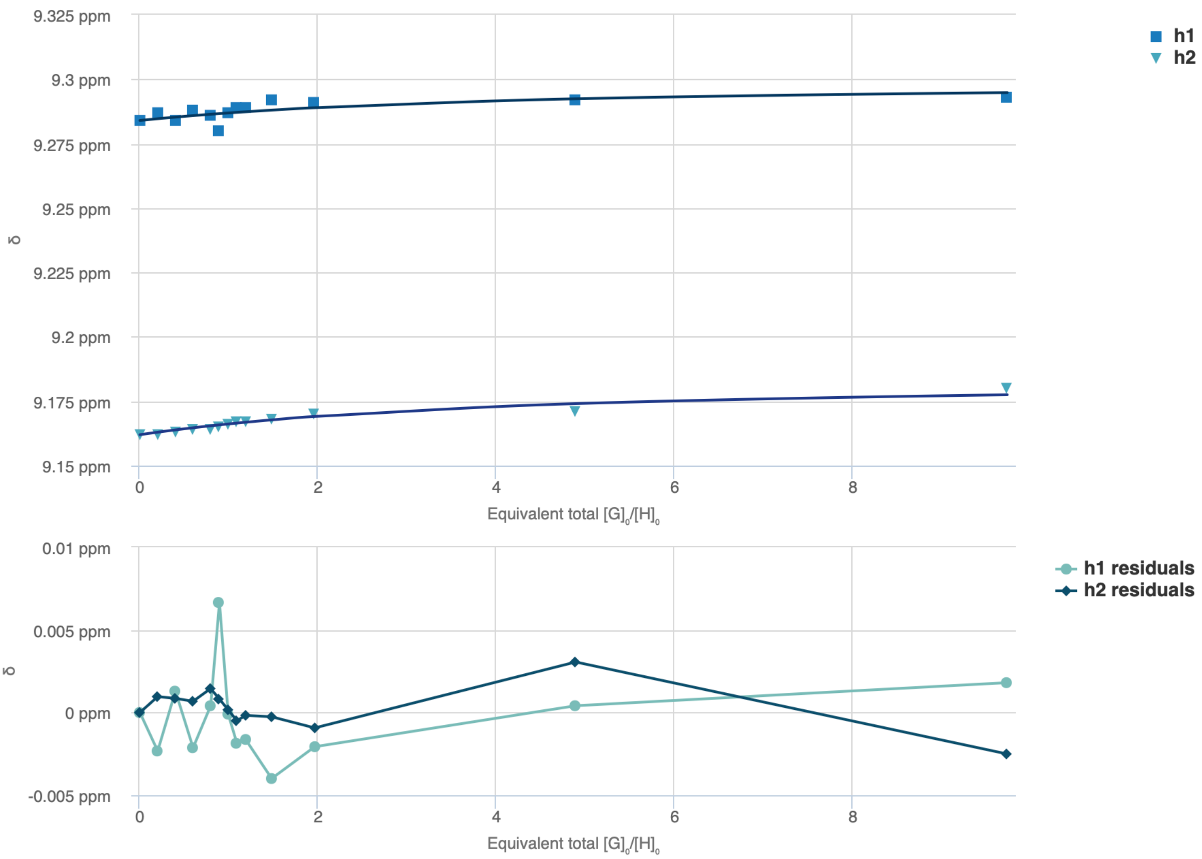

Figure S94. Binding data for $Pd_4L_8(BF_4)_8$ and 4F-benzoate, K_a = 1040.38 M^{-1} ± 35.2%.

4. Cage Interconversion Tests

The interconversion between Pd_4L_8 and Pd_6L_{12} can be triggered by either anions or solvents. The conversion of the cage was monitored by HRMS.

Interconversion of Cage Pd_4L_8 to Pd_6L_{12}

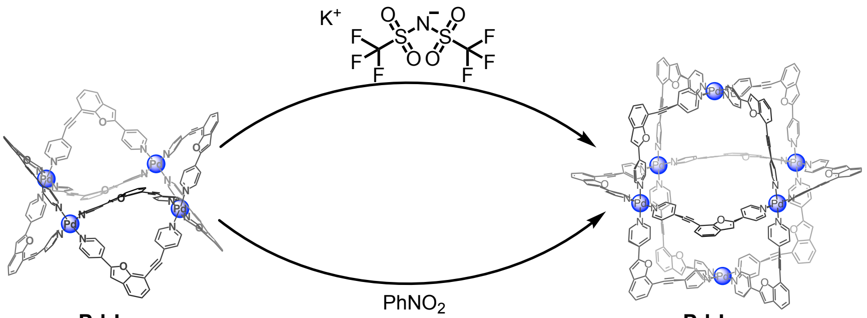

Condition 1 (Anion trigger): In a 2 mL vial, a solution of cage $Pd_4L_8(BF_4)_8$ (0.4 mL, 0.3 mM, 1 eq.) in acetonitrile was added to $KNTf_2$ (1.53 mg, 4.80 µmol, 40 eq.). The mixture was then stirred at 50 °C for 15 mins. The mixture was filtered and diluted for HRMS testing.

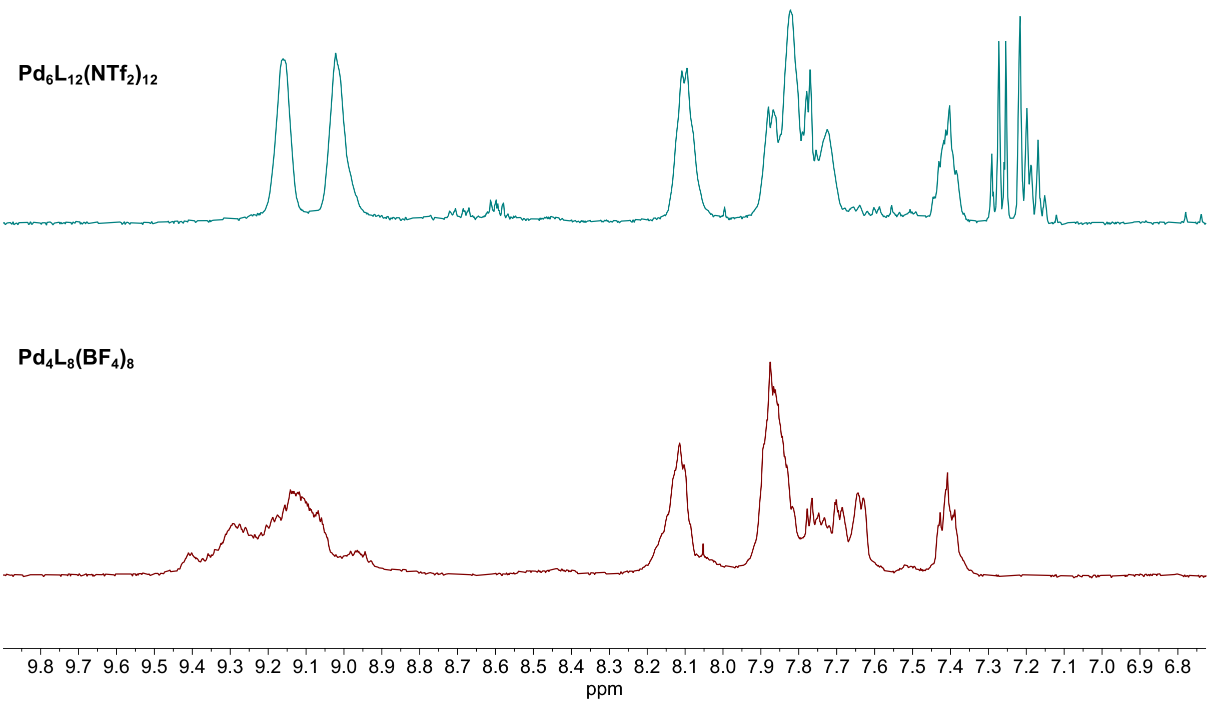

Figure S95. Stacked ^1H NMR for the anion triggered cage interconversion from $Pd_4L_8(BF_4)_8$ (Bottom) to $Pd_6L_{12}(NTf_2)_{12}$ (Top) (d_3-MeCN, 400 MHz, 298 K).

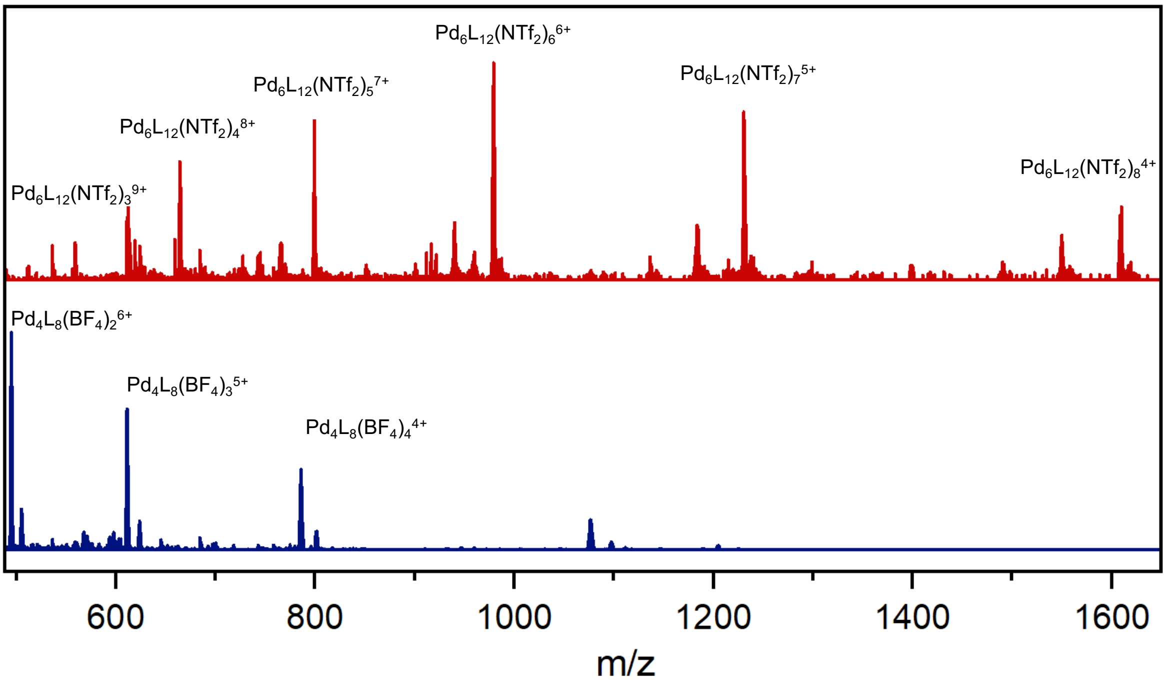

Figure S96. Stacked ESI-MS (positive-ion mode, CH_3CN) for the anion triggered cage interconversion from $Pd_4L_8(BF_4)_8$ (Bottom) to $Pd_6L_{12}(NTf_2)_{12}$ (Top).

Condition 2 (Solution trigger): In a 4 mL vial, excess amount of diethyl ether was added to a solution of cage $Pd_4L_8(BF_4)_8$ (0.4 mL, 0.3 mM, 1 eq.). The precipitated white solid was collected by centrifugation and re-dissolved into 0.4 mL nitrobenzene. The solution was heated at 50 ^{o}C for 4 h and diluted for HRMS testing.

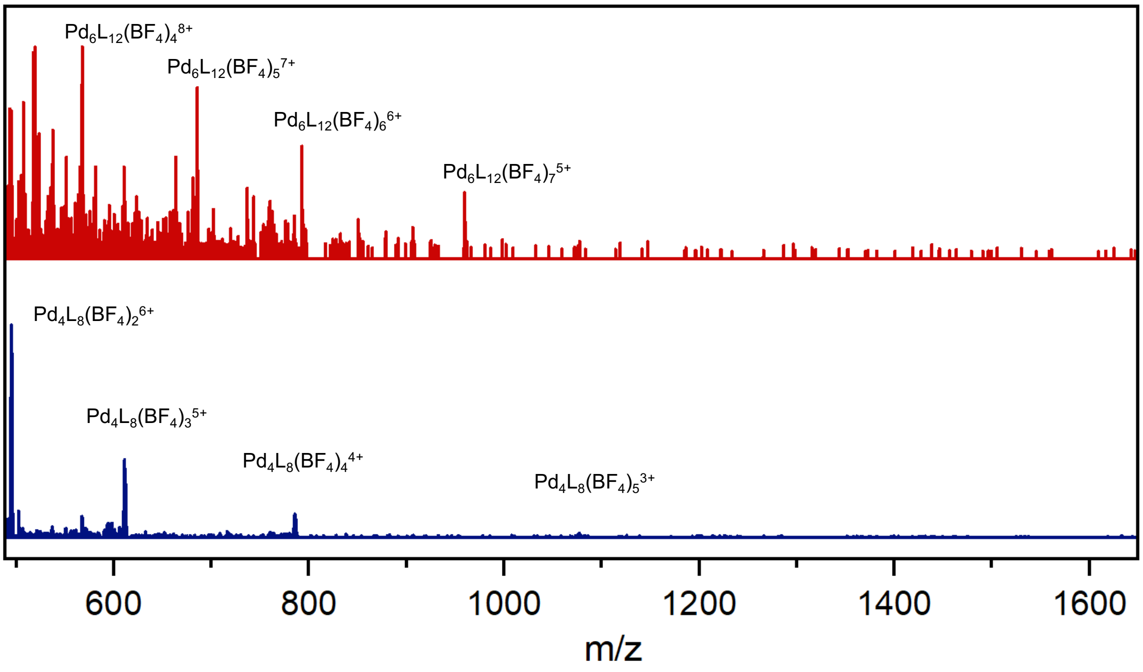

Figure S97. Stacked ESI-MS (positive-ion mode, CH_3CN) for the solvent triggered cage interconversion from $Pd_4L_8(BF_4)_8$ (Bottom) to $Pd_6L_{12}(BF_4)_{12}$ (Top).

Interconversion of Cage Pd_6L_{12} to Pd_4L_8

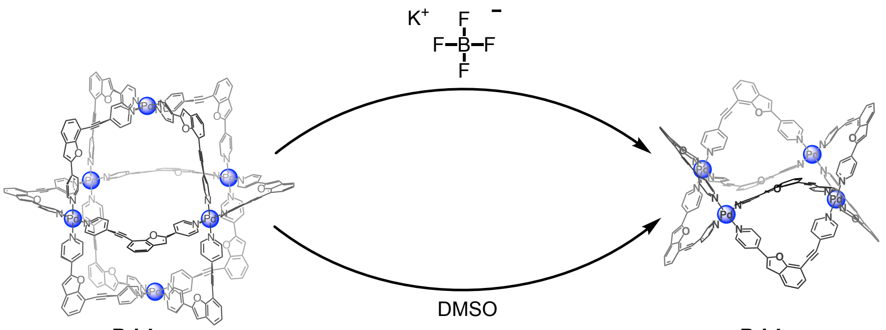

Condition 1 (Anion trigger): In a 2 mL vial, a solution of cage $Pd_6L_{12}(NTf_2)_{12}$ (0.4 mL, 0.3 mM, 1 eq.) in acetonitrile was added to KBF_4 (0.91 mg, 7.20 µmol, 60 eq.), the mixture was then stirred at 50 °C for 15 mins. The mixture was filtered and diluted for HRMS testing.

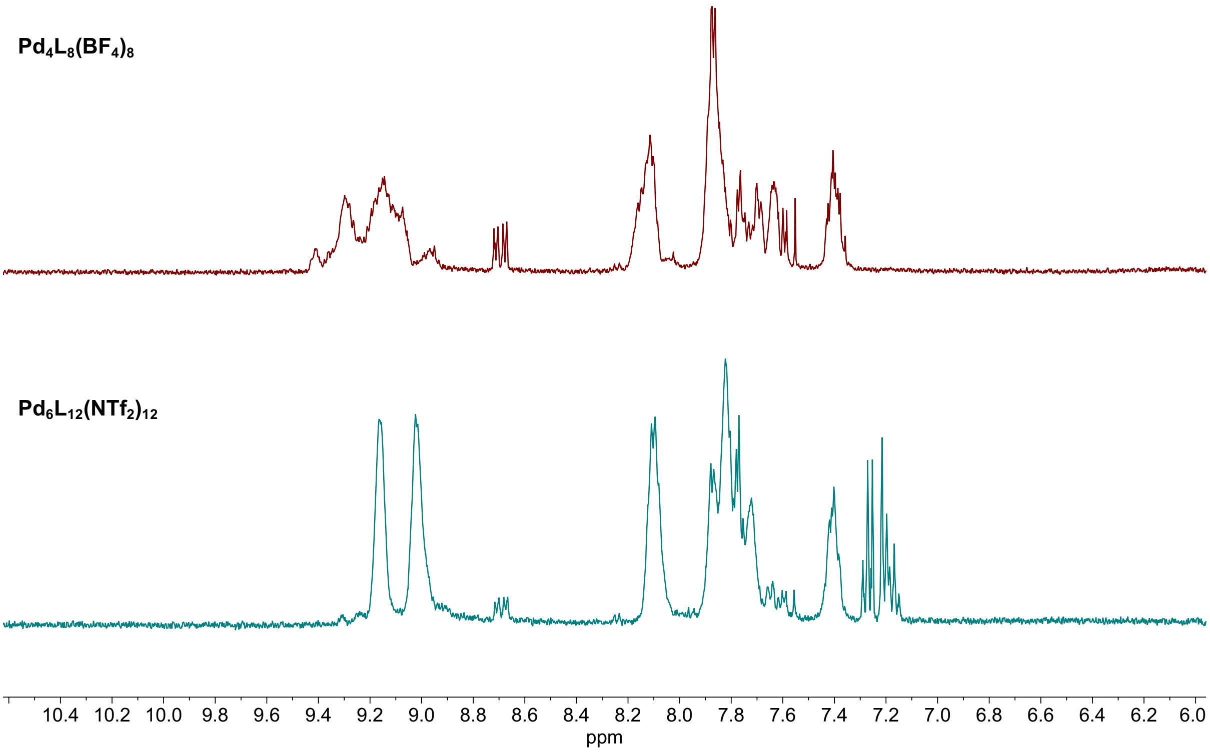

Figure S98. Stacked ^1H NMR for the anion triggered cage interconversion from $Pd_6L_{12}(NTf_2)_{12}$ (Bottom) to $Pd_4L_8(BF_4)_8$ (Top) (d_3-MeCN, 400 MHz, 298 K).

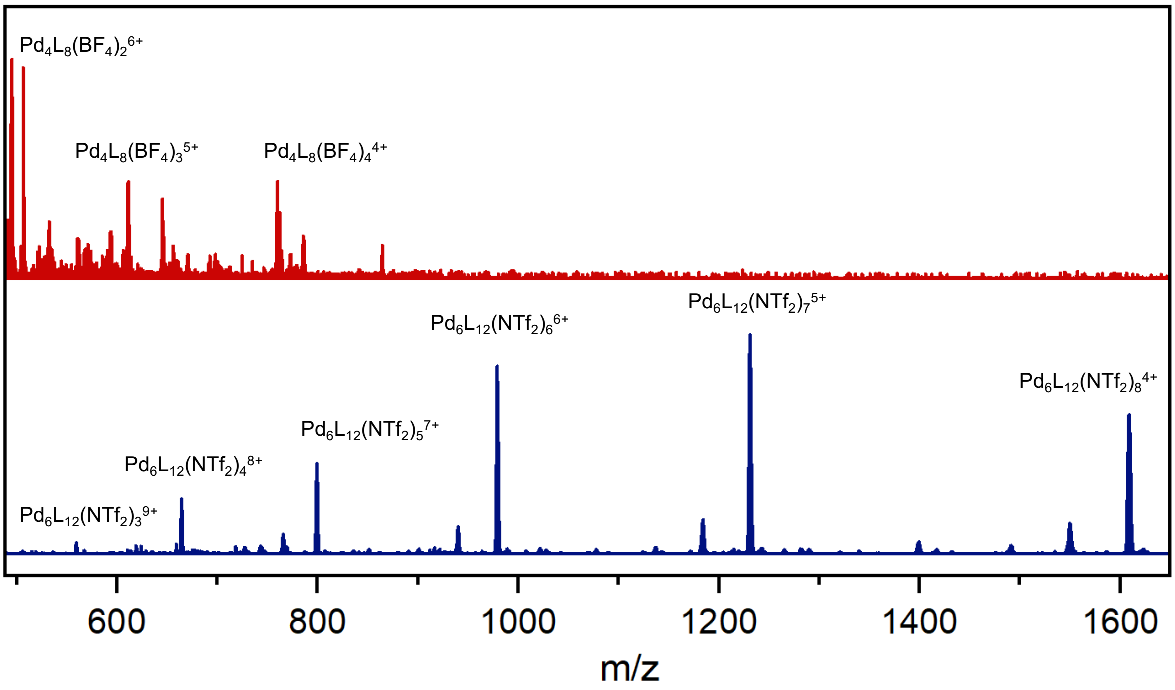

Figure S99. Stacked ESI-MS (positive-ion mode, CH_3CN) for the anion triggered cage interconversion from $Pd_6L_{12}(NTf_2)_{12}$ (Bottom) to $Pd_4L_8(BF_4)_8$ (Top).

Condition 2 (Solution trigger): In a 4 mL vial, excess diethyl ether was added to the solution of cage $Pd_6L_{12}(NTf_2)_{12}$ (0.4 mL, 0.3 mM, 1 eq.). The precipitated white solid was collected by centrifugation and re-dissolved in 0.4 mL DMSO. The solution was heated at 50 ^oC for 4 h and diluted for HRMS testing.

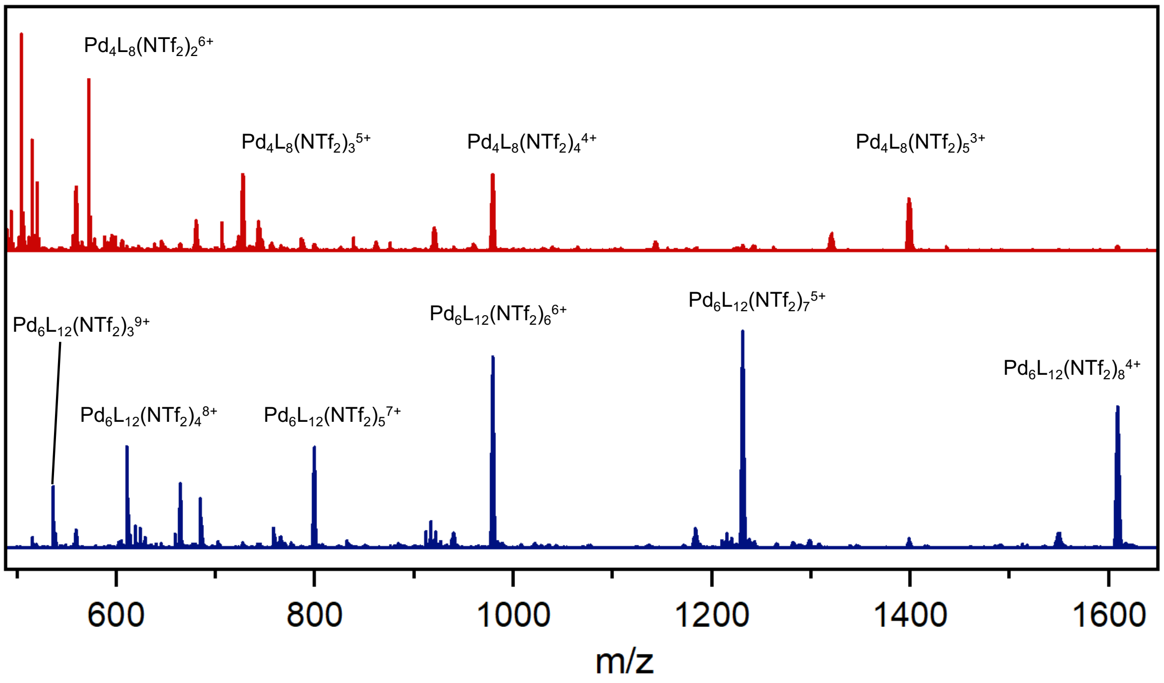

Figure S100. Stacked ESI-MS (positive-ion mode, CH_3CN) for the solvent triggered cage interconversion from $Pd_6L_{12}(NTf_2)_{12}$ (Bottom) to $Pd_4L_8(NTf_2)_8$ (Top).

5. Reversible Guest Binding

To investigate reversible guest exchange, we selected 4-fluorobenzoate (4F-BA^-) and trifluoromethanesulfonate (OTf^-) as guest molecules. Stock solutions of 4-fluorobenzoic acid and tetrabutylammonium triflate (TBA·OTf) were prepared at a concentration of 300 mM in d_3-MeCN. A stock solution of the host cage $Pd_6L_{12}(NTf_2)_{12}$ (0.4 mL, 0.3 mM) was also prepared in d_3-MeCN and transferred to an NMR tube. One equivalent of each guest was sequentially added to the host solution, followed by thorough mixing.

Anionic interconversion triggering additives, KBF_4 and $KNTf_2$, were each prepared as 30 mM stock solutions in d_3-MeCN. Due to the limited solubility of KBF_4 in acetonitrile, the solution was sonicated to ensure uniform dispersion before use.

The solution of cage $Pd_6L_{12}(NTf_2)_{12}$ with both guests in d_3-MeCN was first analysed by ^{19}F NMR to record the initial state. Subsequently, 60 equivalents of KBF_4 (corresponding to 5 equivalents relative to the NTf_2^- anions of the cage) were added to the solution. The mixture was stirred thoroughly and heated at 50 °C for 1 hour to facilitate conversion to cage $Pd_4L_8(BF_4)_8$. The resulting solution was then analysed by ^1H NMR.

To reverse the transformation, 120 equivalents of $KNTf_2$ (10 equivalents relative to the BF_4^- anions of the cage) were added to the same solution. After stirring and heating at 50 °C for 1 hour, ^1H NMR was again recorded, confirming regeneration of cage $Pd_6L_{12}(NTf_2)_{12}$.

This reversible interconversion procedure was repeated for four full cycles, alternating between addition of 120 equivalents of KBF_4 or $KNTf_2$, with ^1H NMR measurements taken after each step to monitor the structural switching.

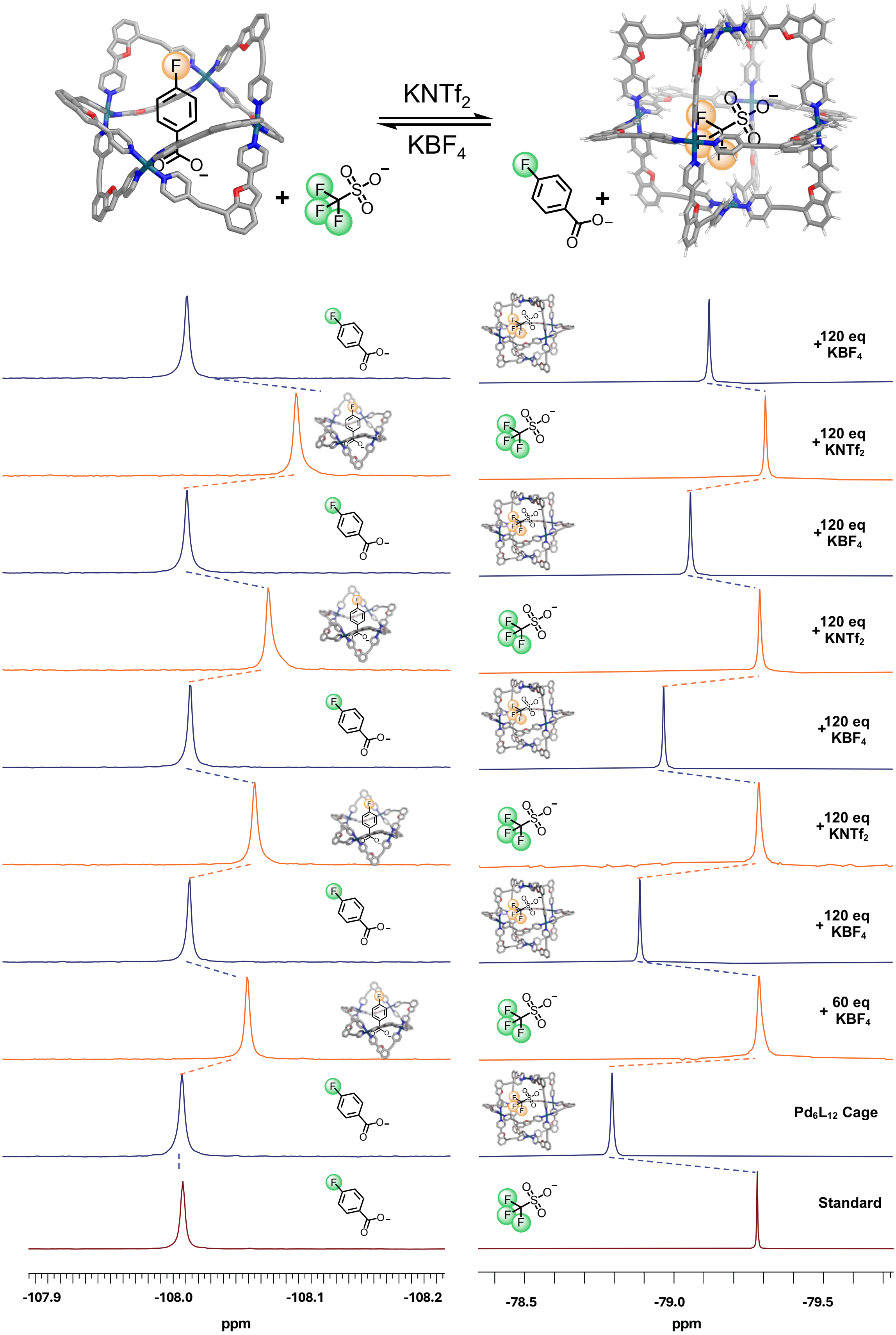

Figure S101. Stacked ^{19}F NMR spectra track the guest binding behavior through four cycles (d_3-MeCN, 376 MHz, 298 K).

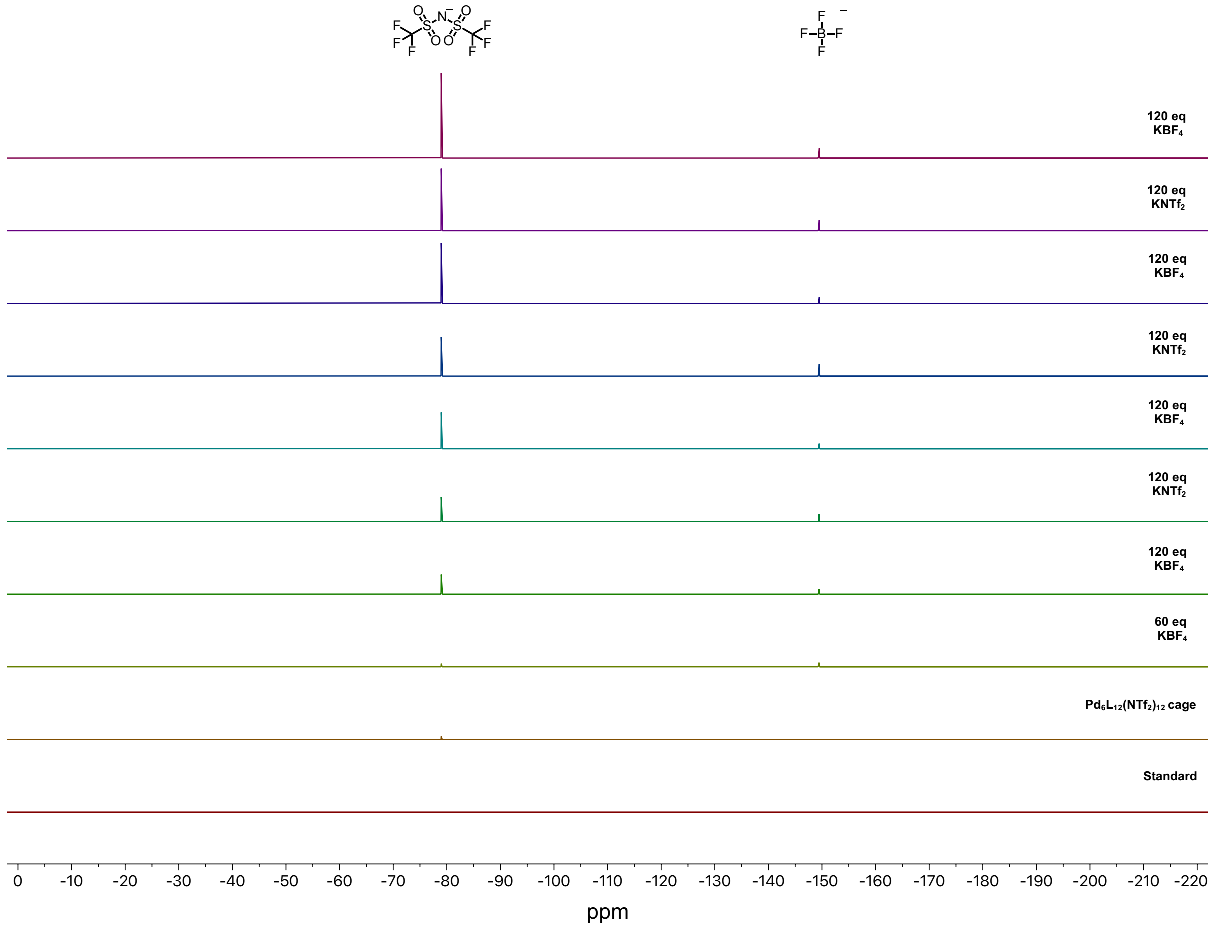

Figure S102. Stacked ^{19}F NMR full spectra track the accumulation of the salts added throughout four cycles (d_3-MeCN, 376 MHz, 298 K).

6. Cage Transformation for Darunavir Purification

To demonstrate the feasibility of guest-selective cage transformation for purifying Darunavir from a complex pharmaceutical mixture, Darunavir (**D**) was selected as the target molecule, with five drugs (**M1**-**M5**) **M1**: Amantadine; **M2**: 4-Fluoro-Thalidomide; **M3**: 4-OH-Thalidomide; **M4**: Oseltamivir; **M5**: Thalidomide; used as control, non-binding, compounds. Stock solutions (30 mM in d_3-MeCN) of all six guest molecules were prepared. In a 4 mL vial, 0.4 mL of a 0.3 mM stock solution of cage $Pd_6L_{12}(NTf_2)_{12}$ in d_3-MeCN was mixed with an equimolar mixture of the six guest molecules to afford a final guest concentration of 0.1 mM, with two equivalents of Cage $Pd_6L_{12}(NTf_2)_{12}$ relative to Darunavir. The solution was vortexed, and equilibrated briefly to allow selective binding between Cage $Pd_6L_{12}(NTf_2)_{12}$ and Darunavir (Step 1).

An excess of diethyl ether (Et_2O) was then added to the solution to induce precipitation of the Darunavir-bound Cage $Pd_6L_{12}(NTf_2)_{12}$ complex (Step 2). The precipitate was collected by centrifugation, and the supernatant was removed. The solid was redissolved in 0.4 mL d_3-MeCN (Step 3), followed by the addition of 60 equivalents of KBF_4. The mixture was heated at 50 °C for 1 hour to fully convert Cage $Pd_6L_{12}(NTf_2)_{12}$ to Cage $Pd_4L_8(BF_4)_8$, thereby triggering the release of the encapsulated Darunavir (Step 4).

An excess of Et_2O was again added to precipitate the resulting Cage $Pd_4L_8(BF_4)_8$, which was separated by centrifugation. The supernatant was concentrated by rotary evaporation to yield purified Darunavir (Step 5). The recovered Cage $Pd_4L_8(BF_4)_8$ can be reconverted to Cage $Pd_6L_{12}(NTf_2)_{12}$ to be reused in subsequent purification cycles (Step 6).

Each step of the process was monitored by ^1H NMR spectroscopy to confirm selective guest encapsulation, successful cage transformation, and efficient release and recovery of the target molecule. HRMS was collected to show that Darunavir, and none of **M1**-**M5** were bound in this context.

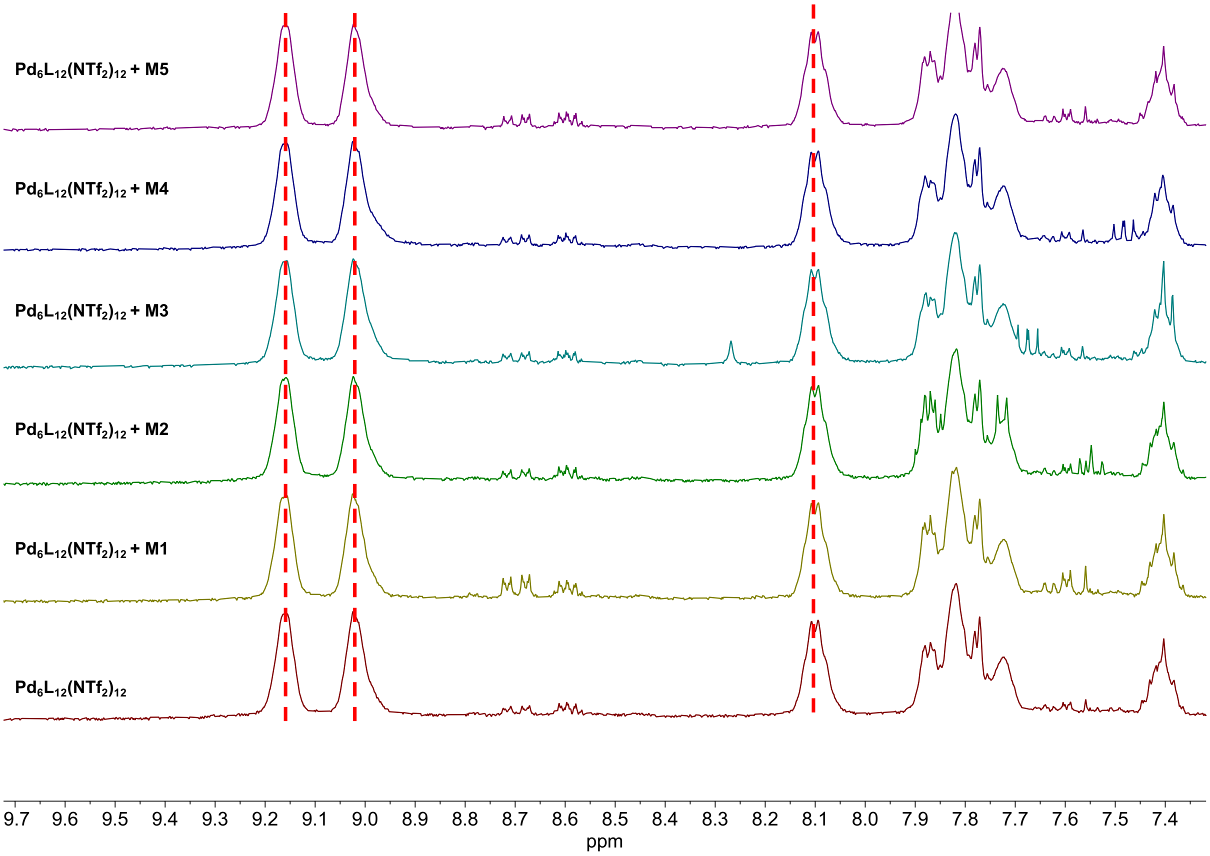

Figure S103. Stacked ^{1}H NMR for the $Pd_6L_{12}(NTf_2)_{12}$ mixed with guests **M1-M5** showing non-binding (d_3-MeCN, 400 MHz, 298 K).

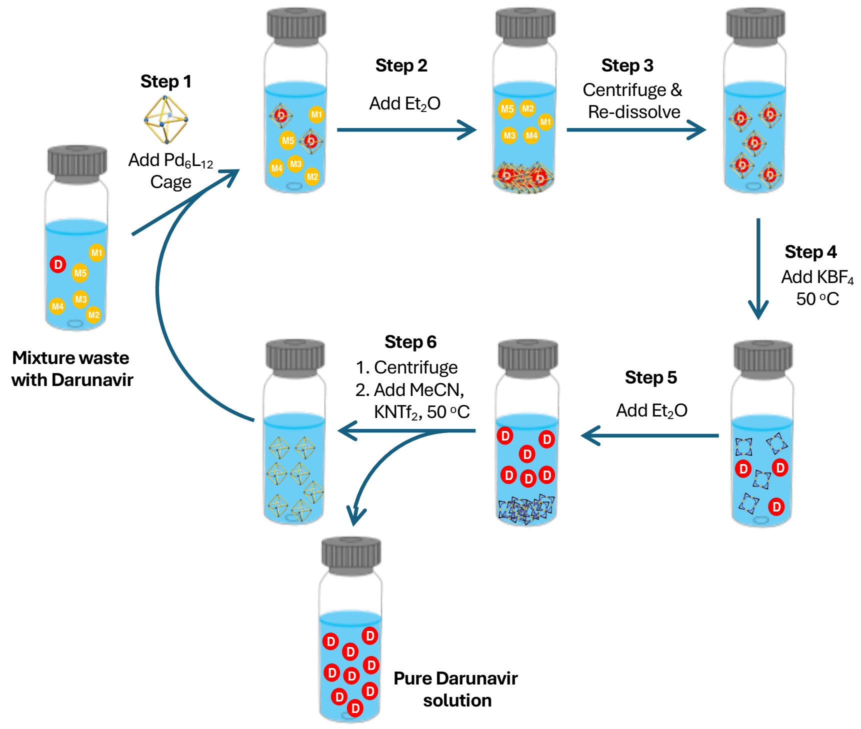

Figure S104. Schematic illustration of the $Pd_6L_{12}(NTf_2)_{12}$-to-$Pd_4L_8(BF_4)_8$ transformation strategy used for selective extraction and purification of Darunavir (**D**) from a mixture of six structurally differentiated pharmaceutical molecules (**D** and **M1**–**M5**).

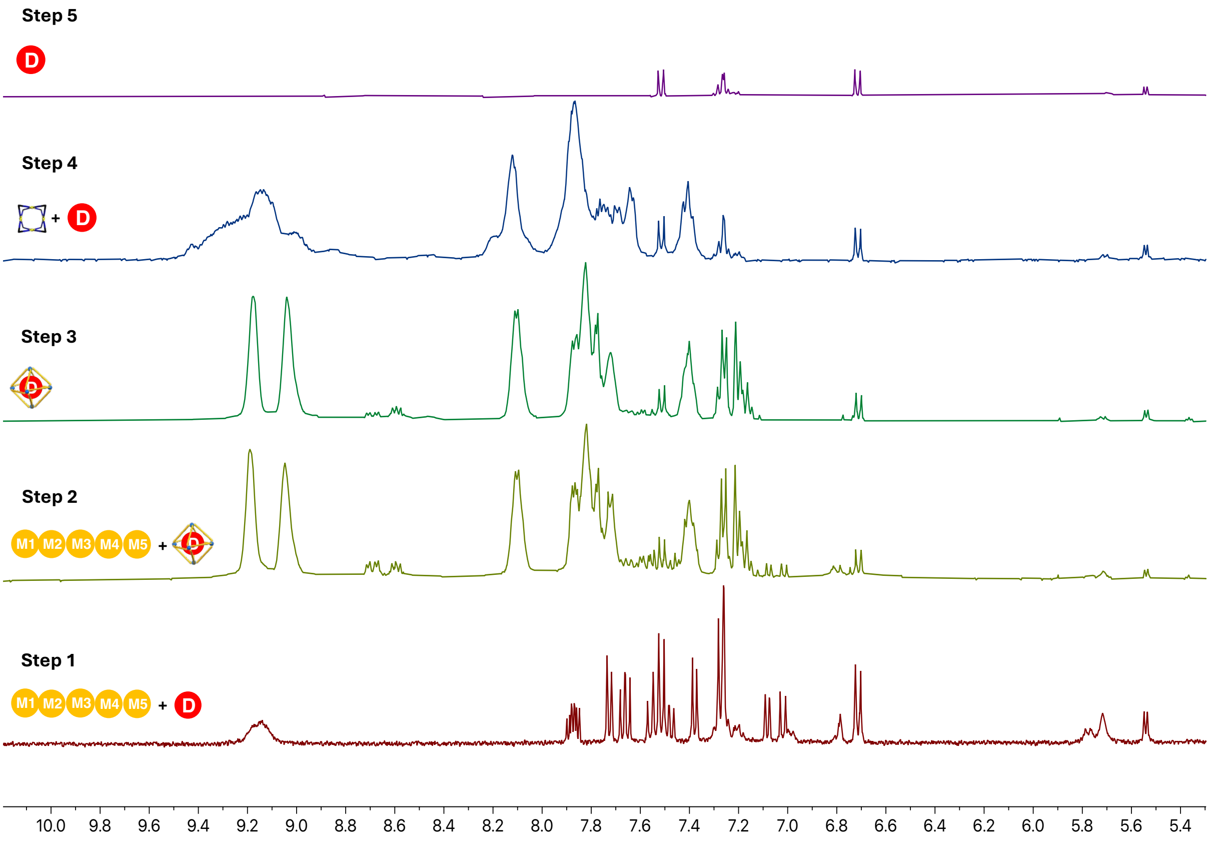

Figure S105. Stacked ^{1}H NMR spectra track for each step in the Darunavir purification cycle (d_3-MeCN, 400 MHz, 298 K).

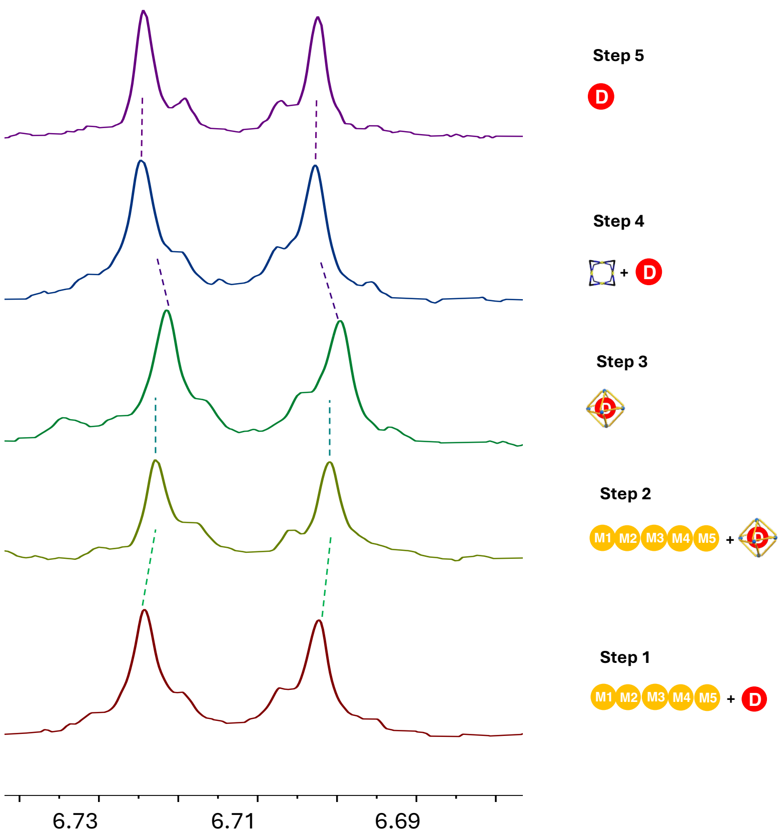

Figure S106. Expanded view of part of the aromatic region (6.68-6.74 ppm), showing host-guest binding-induced shifts upon Cage $Pd_6L_{12}(NTf_2)_{12}$ binding (high-field shift) and release after Cage $Pd_4L_8(BF_4)_8$ formation (low-field shift) (d_3-MeCN, 400 MHz, 298 K).

ICP-MS Analysis and Internal Standard:

To better quantify the separation efficiency of Darunavir, we employed an external standard method, using mesitylene to determine the concentration of Darunavir before and after the purification cycle. Integration results revealed excellent separation performance, with a recovery efficiency exceeding 95%, indicating negligible loss of the drug during the capture-and-release process.

To further eliminate concerns regarding potential heavy metal contamination in the final product, we conducted ICP-MS analysis on the Darunavir purified through our capture-and-release protocol and directly compared it with a commercial reference sample.

The test procedure first add 300 uL trace metal grade nitric acid (FisherScientific) to each sample. And then digested in an over set to 70 °C for 60 min. After cooling, 700 uL 18.2 m-ohm ultrapure water was added inside the micro-centrifuge tube for a

dilution up to 1mL. After vortexing, take 500 uL and diluted up to 10mL to have a duplicate sample. A set of Pd calibration standards were made in the ranging from 0.01 – 1000ppb in 2% trace metal grade nitric acid, and made a 5ppm quality control check standard. The ICP-MS(Perkin Elmer NexION 5000) was then analysed in no gas mode with a Perkin Elmer S23 Autosampler connected to a MiraMist Nebulizer connected to a baffled cyclonic spray chamber. 50ppb Indium internal standard was used to account for any matrix effects. Masses monitored were 106Pd and 115In.

Peer to the results, the residual palladium content in our purified product was below 1 ppb (0.102/0.079 ppb in duplicate measurements), closely matching the levels found in the commercial sample (0.131/0.061 ppb). These values are several orders of magnitude lower than the safety thresholds recommended by relevant pharmaceutical guidelines, confirming that the precipitation step efficiently removes the metal–organic host from the final product.

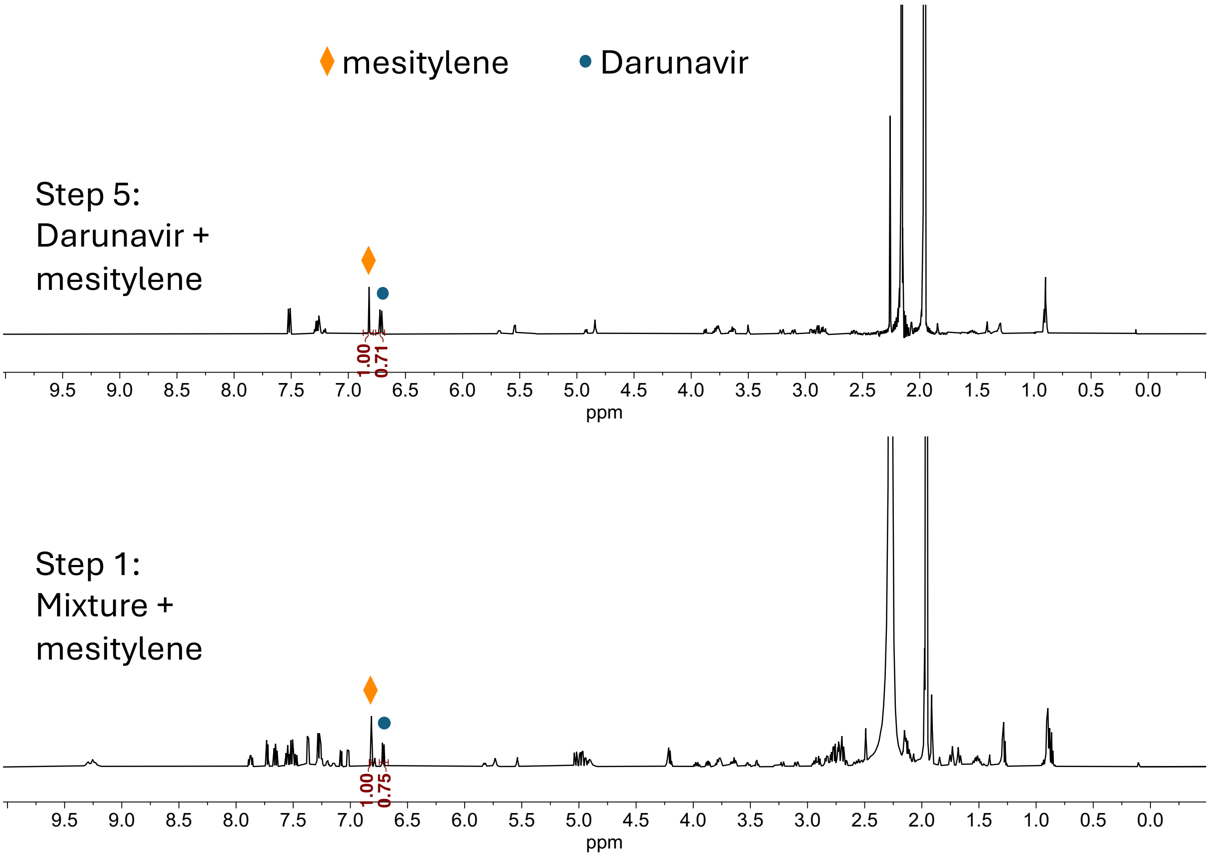

Figure S107. 1H NMR integration ratio between external standard (mesitylene) and the Darunavir before and after cage purification (*d*3-MeCN, 400 MHz, 298 K).

7. X-Ray Crystallography

Data were collected by the National Crystallography Service using a Rigaku FR-E+ High Flux Diffractometer equipped with Mo-Kα radiation (0.71073 Å) with ω at 100(2) K.[2] Data integration and reduction were undertaken with CrysAlisPro version 1.171.43.125a.[3] An analytical numeric absorption correction using a multifaceted crystal model was applied to the data using CrysAlisPro version 1.171.43.125a. Subsequent computations were carried out using the WinGX-32 graphical user interface.[4] The structure was solved by direct methods using SHELXT[5] then refined and extended with SHELXL.[6] In general, non-hydrogen atoms with occupancies greater than 0.5 were refined anisotropically. Carbon-bound hydrogen atoms were included in idealised positions and refined using a riding model. Disorder was modelled using standard crystallographic methods including constraints, restraints and rigid bodies where necessary.

The crystals of $[Pd_4L_8]\cdot 8OTf\cdot 6H_2O$ were grown by diffusion of cyclohexane into a nitrobenzene solution of the complex. The crystals employed were weakly diffracting and few reflections at greater than 1 Å resolution were observed despite collection using a high intensity rotating anode source. The diffraction was broad and the quality of the integration is less than ideal hence the values of the R1, wR and wR2 factors are larger than for typical small molecule structures. Nevertheless, the quality of the data is far more than sufficient to confirm the structure of the Pd_4L_8 assembly. The structure displayed a high degree of crystallographic symmetry with only 1/16 of the Pd_4L_8 assembly (1/4 of a palladium atom and half of a ligand) in the asymmetric unit.

The ligand was disordered end-for-end around a special position with symmetry enforced equal occupancy for each orientation. The two orientations share the same position of the pyridyl ring and only the central portion of the ligand was modelled as disordered. Taking this disorder together with the solution results, the structure can be considered to be a solid solution of different cage diastereomers which are able to crystallise together. Due to the limited resolution and the observed ligand disorder the GRADE program[7] was employed, using the GRADE Web Server,[8] to generate a full set of bond distance and angle restraints (DFIX, DANG, FLAT) for the organic ligand. Thermal parameter restraints (SIMU, RIGU) were applied to all atoms except for palladium to facilitate anisotropic refinement.

The triflate anions within the structure show evidence of substantial disorder. Two lattice sites were located, both heavily disordered around special positions. These anions were either modelled as rigid groups or with extensive DFIX and DANG restraints. The occupancies of the located anions were freely refined which resulted in a discrepancy of 3.61 anions per Pd_4L_8 assembly (or ca. 0.23 per asymmetric unit). Disordered anions were modelled with isotropic thermal parameters due to their low occupancy. The hydrogen atoms of water molecules (located on or close to special positions) could not be located in the electron density map (and are likely disordered) and were therefore not included in the model. This results in CheckCIF alerts for apparent isolated oxygen atoms and short inter D...A contacts as one of the water

molecules located on a 4-fold axis may undergo hydrogen bonding with a triflate anion (itself heavily disordered around a special position).

The remaining anions (included as triflate in the formula) and solvent within the lattice were significantly disordered and despite numerous attempts at modelling, including with rigid bodies no satisfactory model for the electron-density associated with them could be found. Consequently the SQUEEZE[9] function of PLATON[10] was employed to remove the contribution of the electron density associated with these remaining anions and further highly disordered solvent, which gave a potential solvent accessible void of 7877 Å^3 per unit cell (a total of approximately 1347 electrons). The diffuse solvent molecules could not be assigned to cyclohexane into a nitrobenzene and were also not included in the formula. Consequently, the molecular weight and density given above are underestimated.

CheckCIF gives one A and ten B level alerts. Nine of the B level alerts result from the water molecule for which hydrogen atoms were not modelled. The remaining alerts result from the limited resolution of the data (low sine(theta_max)/wavelength) and poor diffraction properties of the crystals (high wR2 value).

Crystallographic data have been deposited with the CCDC (2499075).

$[Pd_4L_8]\cdot 8OTf\cdot 6H_2O$ [+ solvent]

Formula $C_{168}H_{108}F_{24}N_{16}O_{38}Pd_4S_8$, *M* 4096.78, Tetragonal, space group I4/mmm (#139), *a* 25.3920(4), *b* 25.3920(4), *c* 23.7644(12) Å, *V* 15322.2(9) Å^3, D_c 0.888 g cm^{-3}, *Z* 2, crystal size 0.270 by 0.170 by 0.080 mm, colour colourless, habit block, temperature 100(2) Kelvin, λ(MoKα) 0.71073 Å, μ(MoKα) 0.346 mm^{-1}, T(Analytical)$_{min,max}$ 0.877, 0.955, $2\theta_{max}$ 41.64, *hkl* range -25 25, -25 25, -23 23, *N* 95279, N_{ind} 2278(R_{merge} 0.0494), N_{obs} 1594(I > 2σ(I)), N_{var} 201, residuals* $R1(F)$ 0.1354, $wR2(F^2)$ 0.3669, GoF(all) 1.005, $\Delta\rho_{min,max}$ -0.784, 1.374 e$^-$ Å^{-3}.

*$R1 = \Sigma||F_o| - |F_c||/\Sigma|F_o|$ for $F_o > 2\sigma(F_o)$; $wR2 = (\Sigma w(F_o^2 - F_c^2)^2/\Sigma(wF_c^2)^2)^{1/2}$ all reflections

$w=1/[\sigma^2(F_o^2)+(0.1200P)^2+280.0000P]$ where $P=(F_o^2+2F_c^2)/3$

8. DFT calculation

All Density Functional Theory (DFT) calculations were performed using the ORCA program package (Version 5.0).[11-12] To accurately describe the energetics and geometries of the large supramolecular palladium assemblies driven by non-covalent interactions, the composite r2SCAN-3c method was employed.[13] The r2SCAN-3c method is based on the meta-GGA r2SCAN functional[14] and utilizes a modified triple-zeta basis set (mTZ2P) tailored for this specific functional. Crucially, this composite method explicitly includes the D4 London dispersion correction[15-16] to account for long-range non-covalent interactions, as well as the geometrical counterpoise (gCP) correction[17] to mitigate basis set superposition error (BSSE), both of which are critical for the accurate modelling of large encapsulated host-guest systems.

To explore the conformational space of the isomeric mixtures, representative isomers for both the octahedral (Oh) and hexagonal macrocyclic (MC) topologies were constructed using Cgbind[18] and optimized under same condition. The isomers were selected to sample distinct ligand arrangements (e.g., unidirectional vs. random head-to-tail orientations) to verify the energetic preference of the octahedral geometry regardless of local ligand conformation.

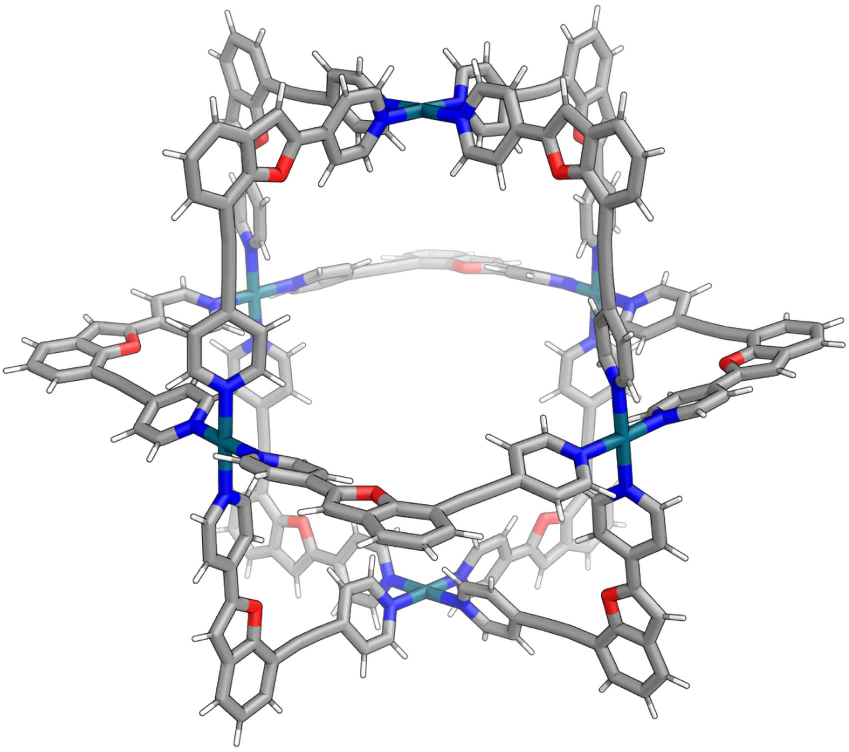

Figure S108. DFT optimized Pd_6L_{12} cage in an Octahedral-Unidirectional format, considering an arbitrarily chosen single isomer.

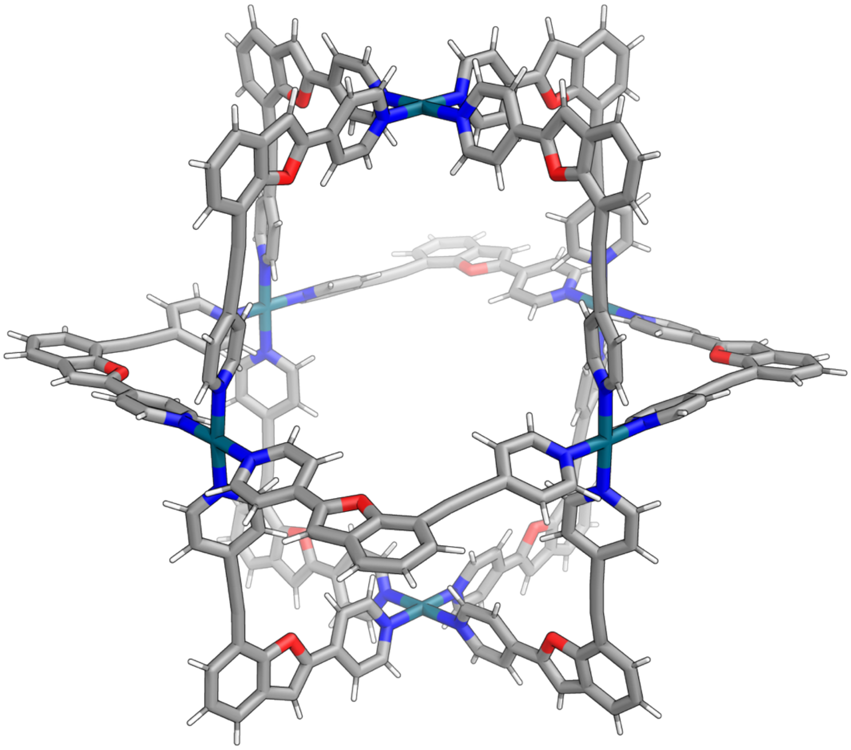

Figure S109. DFT optimized Pd_6L_{12} cage in an Octahedral-Clustered format, considering an arbitrarily chosen single isomer.

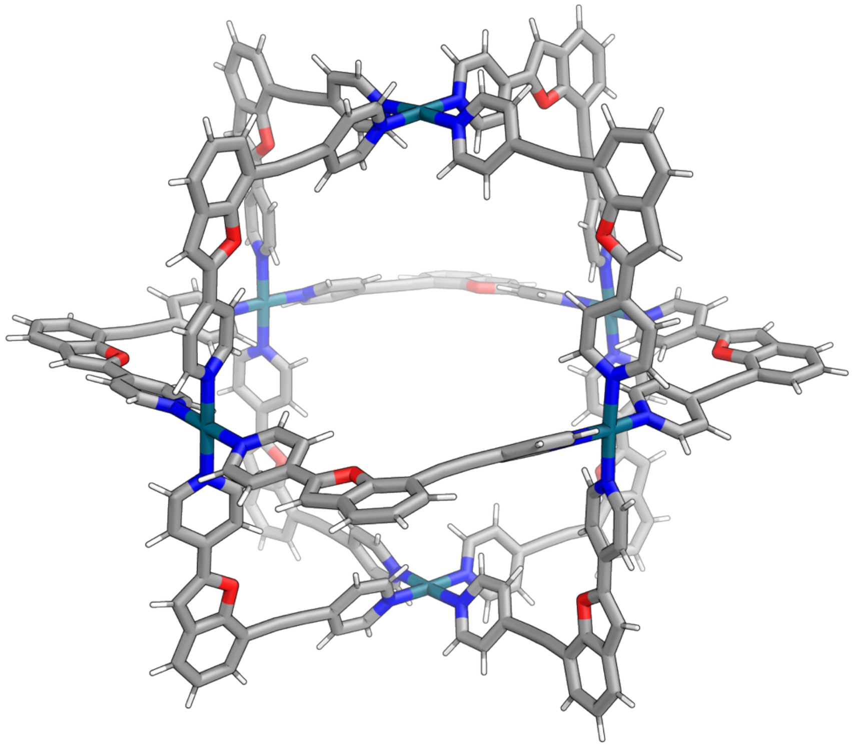

Figure S110. DFT optimized Pd_6L_{12} cage in an Octahedral-Random format, considering an arbitrarily chosen single isomer.

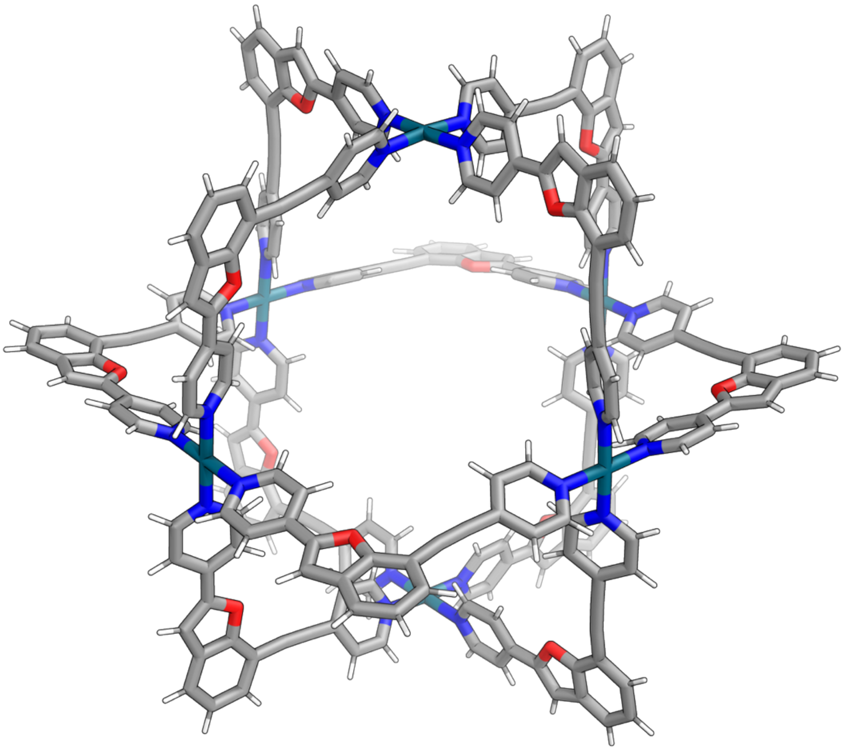

Figure S111. DFT optimized Pd_6L_{12} cage in an Octahedral-Random format, considering an arbitrarily chosen single isomer.

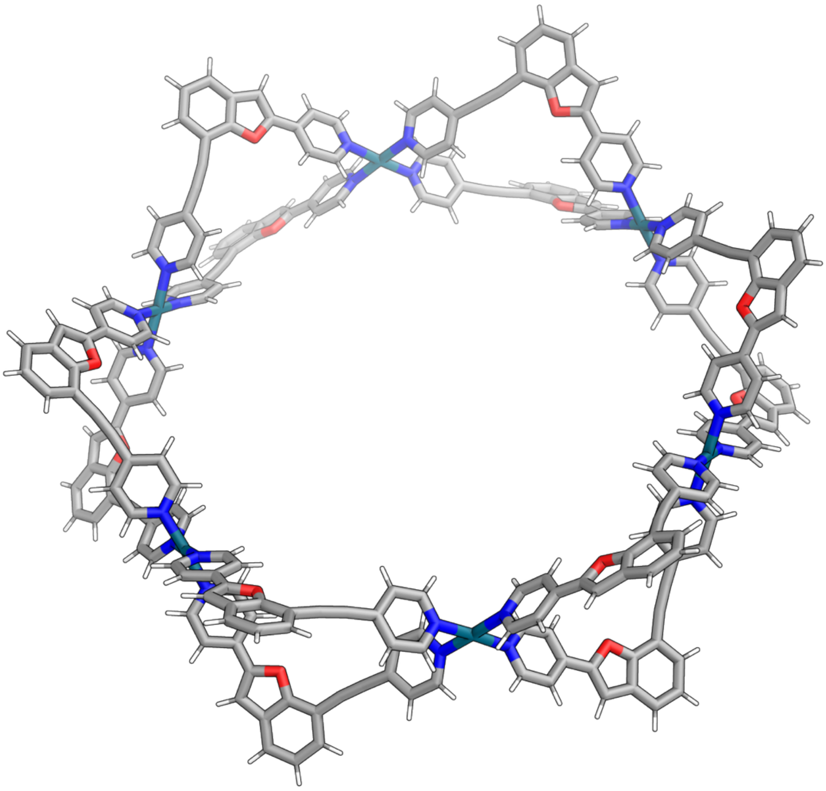

Figure S112. DFT optimized Pd_6L_{12} cage in a Marco Cyclic-Unidirectional format, analogous to the crystal structure of Pd_4L_8, considering an arbitrarily chosen single isomer.

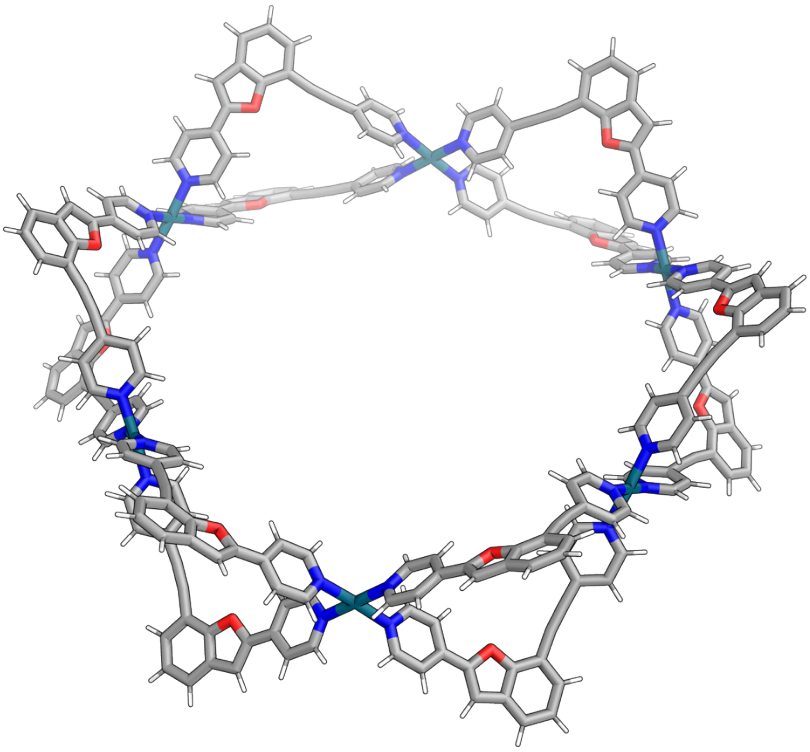

Figure S113. DFT optimized Pd_6L_{12} cage in a Macrocyclic-Clustered format, analogous to the crystal structure of Pd_4L_8, considering an arbitrarily chosen single isomer.

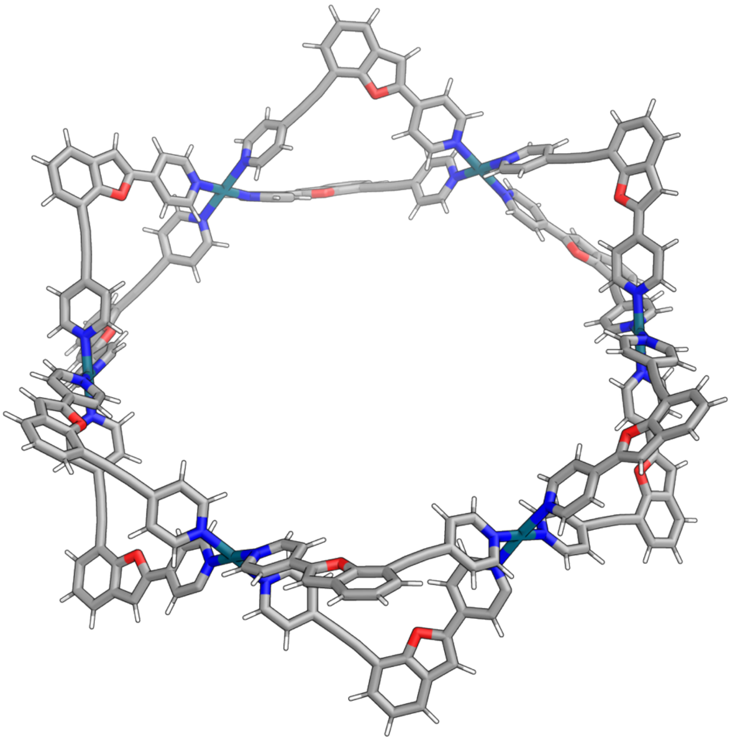

Figure S114. DFT optimized Pd_6L_{12} cage in a Macrocyclic-Face-Differentiated format, analogous to the crystal structure of Pd_4L_8, considering an arbitrarily chosen single isomer.

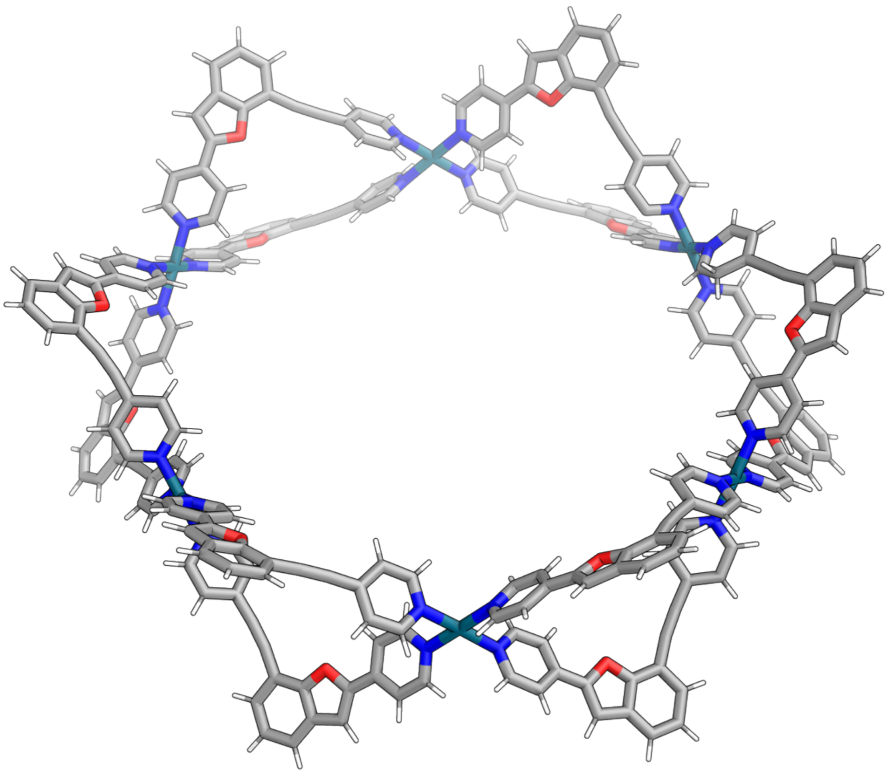

Figure S115. DFT optimized Pd_6L_{12} cage in a Macrocyclic-Random format, analogous to the crystal structure of Pd_4L_8, considering an arbitrarily chosen single isomer.

Table S1. Single point energy comparisons of Pd_6L_{12} cage in different geometries

	Pd_6L_{12} isomers	Energy (Hartree)
Oh isomers	Oh-1-Unidirectional	-12209.61568
	Oh-2-Clustered	-12209.61098
	Oh-3-random1	-12209.61185
	Oh-4-random2	-12209.61382
MC isomers	MC-1-Unidirectional	-12209.57236
	MC-2- Clustered	-12209.47699
	MC-3- Face-Differentiated	-12209.45893
	MC-4- random	-12209.38692

To further explore the binding interaction between Darunavir and the Pd_6L_{12} cage using DFT, we performed geometry optimizations on the Pd_6L_{12} – Darunavir complex using the same computational methodology. A representative cage isomer with randomly arranged ligands was selected to reflect the low-symmetry configurations typically observed. One Darunavir molecule was placed inside the cage prior to optimization. The results indicate a good size complementarity between the Pd_6L_{12} cage and the Darunavir molecule, without excessive steric crowding. The space-filling

model further highlights the close packing of Darunavir against the inner cage walls, supporting a strong host-guest interaction between the cage and Darunavir.

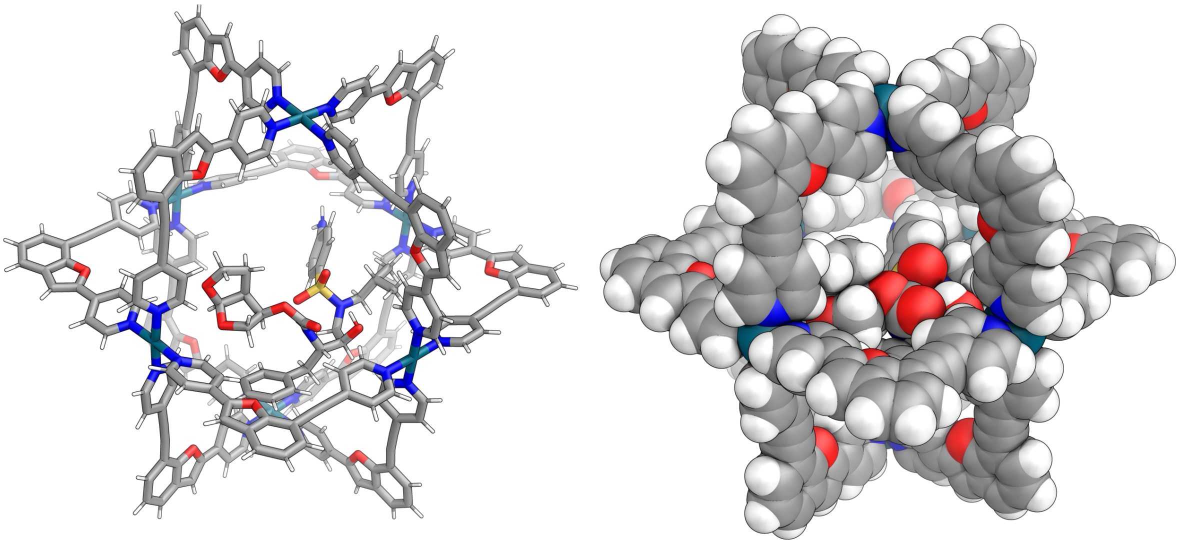

Figure S116. DFT optimized Pd_6L_{12} cage – Darunavir complex structure shows as ball and stick model (left) and space filling model (right).

Stability of Pd_6L_{12} to addition of external Pyridine:

On analysing our computational models, we saw a level of deviation from idealized 90° bond angles expected in square planar metals (88 - 92°). To assess the effect on stability of the deviations from 90° bond angles found in our computational models, we performed a titration of cage against pyridine. Here, we found a reduced level of stability as compared to previously reported cages, with 40 equivalents of pyridine sufficient to degrade half of the cage. As such, this indicates the octahedral structure may sit in a shallower potential energy well, do to strain induced in the system, providing a potential explanation for the observed stimuli responsive behaviour.

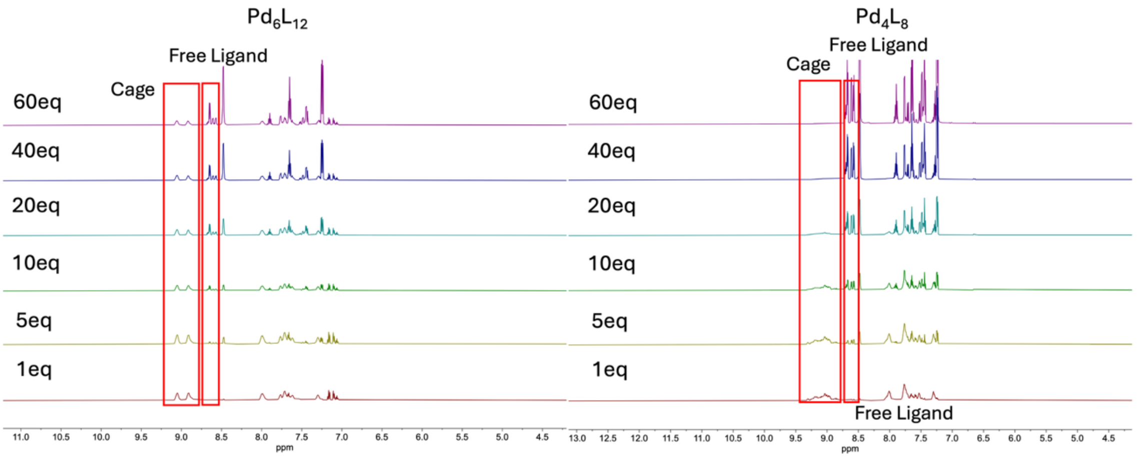

Figure S117. Stacked ^{1}H NMR for the $Pd_6L_{12}(NTf_2)_{12}$ and $Pd_4L_8(BF_4)_4$ with a titration against pyridine (d_3-MeCN, 600 MHz, 298 K).

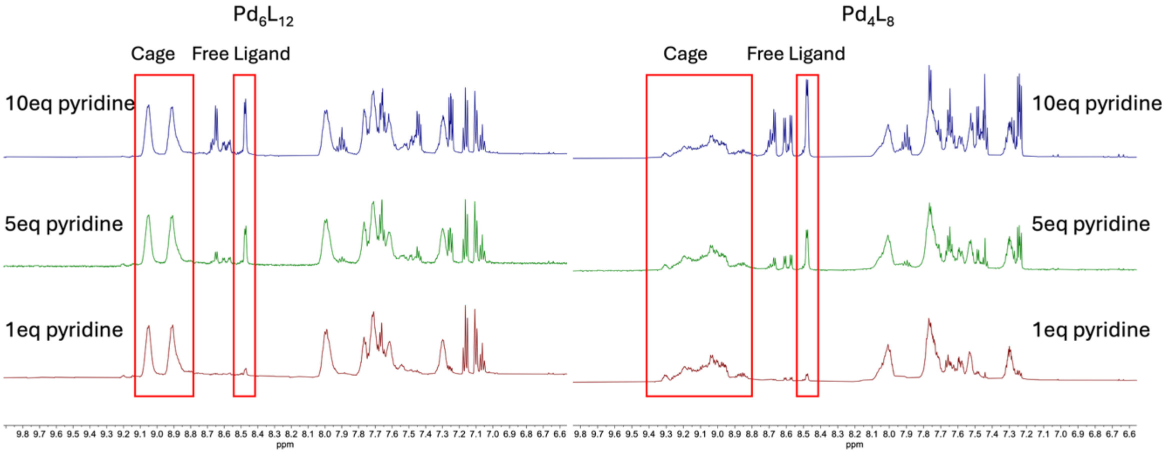

Figure S118. Stacked ^{1}H NMR for the $Pd_6L_{12}(NTf_2)_{12}$ and $Pd_4L_8(BF_4)_4$ with a titration against pyridine (d_3-MeCN, 600 MHz, 298 K). Focused view, showing clear increase in free ligand.

9. References

[1] K. Suzuki, M. Kawano, S. Sato, M. Fujita, Endohedral peptide lining of a self-assembled molecular sphere to generate chirality-confined hollows *Journal of the American Chemical Society* **2007**, *129*, 10652-10653.

[2] S. J. Coles, D. R. Allan, C. M. Beavers, S. J. Teat, S. J. Holgate, C. A. Tovee, in *21st Century Challenges in Chemical Crystallography I: History and Technical Developments*, Springer, **2020**, pp. 69-140.

[3] R. O. Diffraction, Oxford, UK Search PubMed, **2015**.

[4] L. Farrugia, WinGX and ORTEP for Windows: an update *J. Appl. Crystallogr.* **2012**, *45*, 849-854.

[5] G. Sheldrick, SHELXT - Integrated space-group and crystal-structure determination *Acta. Cryst.* **2015**, *A71*, 3-8.

[6] G. M. Sheldrick, Crystal structure refinement with SHELXL *Acta. Cryst.* **2015**, *C71*, 3-8.

[7] G. Bricogne, E. Blanc, M. Brandle, C. Flensburg, P. Keller, W. Paciorek, P. Roversi, A. Sharff, O. S. Smart, C. Vonrhein, T. O. Womack, *BUSTER*, 2.11.2 ed., Global Phasing Ltd., Cambridge, United Kingdom, **2011**.

[8] O. S. Smart, T. O. Womack, *Grade Web Server*, Global Phasing Ltd., **2014**.

[9] P. van der Sluis, A. L. Spek, BYPASS: an effective method for the refinement of crystal structures containing disordered solvent regions *Acta Cryst.* **1990**, *A46*, 194-201.

[10] A. L. Spek, *PLATON: A Multipurpose Crystallographic Tool*, Utrecht University, Utrecht, The Netherlands, **2008**.

[11] F. Neese, F. Wennmohs, U. Becker, C. Riplinger, The ORCA quantum chemistry program package *The Journal of Chemical Physics* **2020**, *152*.

[12] F. Neese, The ORCA program system *Wiley Interdisciplinary Reviews: Computational Molecular Science* **2012**, *2*, 73-78.

[13] S. Grimme, A. Hansen, S. Ehlert, J.-M. Mewes, r2SCAN-3c: A “Swiss army knife” composite electronic-structure method *The Journal of Chemical Physics* **2021**, *154*.

[14] J. W. Furness, A. D. Kaplan, J. Ning, J. P. Perdew, J. Sun, Accurate and numerically efficient r2SCAN meta-generalized gradient approximation *The Journal of Physical Chemistry Letters* **2020**, *11*, 8208-8215.

[15] E. Caldeweyher, S. Ehlert, A. Hansen, H. Neugebauer, S. Spicher, C. Bannwarth, S. Grimme, A generally applicable atomic-charge dependent London dispersion correction *The Journal of Chemical Physics* **2019**, *150*.

[16] E. Caldeweyher, C. Bannwarth, S. Grimme, Extension of the D3 dispersion coefficient model *The Journal of Chemical Physics* **2017**, *147*.

[17] H. Kruse, S. Grimme, A geometrical correction for the inter-and intra-molecular basis set superposition error in Hartree-Fock and density functional theory calculations for large systems *The Journal of Chemical Physics* **2012**, *136*.

[18] T. A. Young, R. Gheorghe, F. Duarte, cgbind: A python module and web app for automated metallocage construction and host–guest characterization *Journal of Chemical Information and Modeling* **2020**, *60*, 3546-3557.
